# Robust optimal reinsurance and ESG investment under rating-innovation ambiguity and ESG rating uncertainty

**DOI:** 10.1371/journal.pone.0354616

**Published:** 2026-11-02

**Authors:** Jing Fu, Guiling Zhu, Guoyong Zhou

**Affiliations:** 1 School of Mathematics and Statistics, Zhaotong University, Zhaotong, Yunnan, China; 2 School of Mathematics and Statistics, Leshan Normal University, Leshan, Sichuan, China; Roma Tre University: Universita degli Studi Roma Tre, ITALY

## Abstract

ESG ratings are noisy measurements and may also be exposed to systematic credibility concerns. We study an ambiguity-averse insurer that chooses proportional reinsurance and green and brown stock positions while learning a hidden green-quality factor from stock returns and an ESG rating signal. Signal volatility represents ordinary rating uncertainty. A worst-case distortion of the rating innovation represents ambiguity about the observation model, whereas a separate out-of-model drift $g_{true}$ is used only in stress tests for systematic rating bias. This distinction prevents robust-control ambiguity from being interpreted as observed greenwashing itself. We derive the reference Kalman–Bucy filter and the separated robust control problem. Shared stock-return shocks produce a mixed wealth-belief derivative and a filtering-hedging demand. Under CARA utility, the value function is exponential-quadratic, stock feedback follows a Riccati-type system, and retention is projected onto q∈[0,1]. The verification result is conditional on explicit admissibility and integrability conditions. Primary numerical experiments with illustrative parameters identify the model mechanisms but do not constitute empirical calibration. Independent numerical checks add Monte Carlo intervals, static and insurance-only benchmarks, original-scale plots, and a 40-design parameter-uncertainty analysis. The learning-strategy intervals overlap substantially, and rating-innovation ambiguity effects are small in CE and warning probability. Tail-risk changes are more directionally consistent across the tested parameter region, but their magnitudes remain small. The numerical evidence therefore supports mechanism existence and conditional downside sensitivity, not universal or quantitatively calibrated economic relevance.

## 1 Introduction

Climate-related information and sustainable-investment preferences have become part of the way investors assess expected returns, risk premia, and portfolio demand. In equilibrium models with green and brown assets, environmental preferences and climate-risk hedging motives can lower the expected returns of green assets, while shocks to climate concerns may generate realized outperformance over some periods [1,2]. Related work shows that ESG scores can enter portfolio choice both as information about fundamentals and as a preference attribute, and that carbon risk and climate-risk perceptions are priced or managed by institutional investors [3–6]. For insurance companies, these issues arise in a particularly concrete form. Insurers are long-horizon investors, but their investment decisions are tied to underwriting risk and reinsurance demand. Green and brown asset allocation therefore cannot be separated cleanly from the insurer’s surplus management problem. Evidence on green bonds and crisis-period ESG performance also suggests that ESG information may matter for capital allocation and resilience, although the magnitude and interpretation of these effects depend on the context [7–9].

A central difficulty is that ESG ratings are not frictionless measurements of corporate sustainability. A growing literature documents substantial disagreement across rating agencies and shows that the divergence is related to differences in scope, measurement, weighting schemes, disclosure, and provider-specific effects [10–12]. Rating disagreement has also been linked to stock returns, portfolio choice, and broader ESG uncertainty [13,14]. Recent work continues to study the determinants and portfolio consequences of rating disagreement, including market and institutional factors and multi-provider portfolio construction [15–17]. These findings motivate treating ESG ratings as noisy signals rather than as direct observations of a firm’s true green quality. In a dynamic investment problem, this distinction matters because the investor must learn from the signal while recognizing that the signal itself may be imperfect.

Noise is not the only concern. The greenwashing literature emphasizes that firms may selectively disclose environmental information or communicate in a way that creates an overly favorable impression of environmental performance [18,19]. Selective disclosure can reflect institutional pressures, scrutiny, reporting incentives, and strategic interaction with auditors or activists [20,21]. This literature motivates, but is not mathematically identical to, the robust-control device used here. In the analytical model, $\phi_{4}$ is a worst-case drift distortion of the rating innovation and therefore represents ambiguity about the observation model. It is not an observed or estimated act of greenwashing. In the numerical stress test, $g_{true}$ is instead an exogenous systematic bias in the data-generating rating process. We reserve “rating-innovation ambiguity” for $\phi_{4}$ and “systematic rating-bias misspecification” for $g_{true}$. Ordinary rating noise remains distinct from both and is governed by $\sigma_{Y}$.

Robust control provides a tractable language for this distinction. The decision maker evaluates controls under a reference model but allows statistically nearby alternatives to distort the dynamics, with the penalty governing the degree of ambiguity aversion [22,23]. Related applications show how robustness changes experimentation, portfolio demand, and asset pricing when the model is misspecified [24]. Recent ESG portfolio models introduce ambiguity into sustainable investment decisions and show that ambiguity can reduce demand for green assets or alter green–brown allocation [25,26]. The present paper is close in spirit to that literature, but the decision problem is different. The investor is an insurer, the hidden state is learned through several observation channels, and the control includes proportional reinsurance as well as green and brown stock positions.

The paper also relates to the actuarial literature on reinsurance-investment problems under partial information, learning, and ambiguity aversion. Partial-information reinsurance-investment models use filtering to transform unobserved-state problems into separated control problems, while robust reinsurance models study how ambiguity changes contract demand, investment, and retention [27,28]. Other recent contributions analyze robust reinsurance-investment problems with biased premiums, competitive and cooperative insurers, or stochastic differential games [29–31]. Insurance-related green finance has also been studied from the perspective of carbon policy and insurer balance-sheet effects [32]. The filtering, separation, Girsanov, and pointwise Hamiltonian steps are classical in stochastic control under partial observation and controlled Markov dynamics [33,34]; recent treatments of Kalman–Bucy filtering emphasize the stability and structure of linear-Gaussian systems [35]. Our contribution is not a new filtering theorem. It is the model-specific coupling of stock-return learning, ESG-information ambiguity, constrained reinsurance, and insurer wealth, together with the mixed wealth-belief term and its policy consequences.

The resulting gap is specific. The ESG rating uncertainty literature usually focuses on asset pricing or portfolio choice for general investors, rather than on insurers that jointly manage underwriting and investment risk. The greenwashing literature mainly studies disclosure and firm behavior, rather than dynamic robust control. Robust ESG portfolio models usually abstract from proportional reinsurance. Conversely, reinsurance-investment models usually do not distinguish rating noise, observation-model ambiguity, and systematic rating bias, nor do they treat both green and brown stock returns as learning signals. If the same stock-return shocks affect wealth and beliefs, market risk and information updating are intentionally coupled. This coupling means the framework does not fully disentangle financial risk from information-quality uncertainty in $\phi_{1}$ and $\phi_{2}$; only the rating channel $\phi_{4}$ is information-only.

This paper makes four model-specific contributions. First, it formulates an insurer problem with joint learning from green returns, orthogonalized brown returns, and an ESG rating while reinsurance and both stock positions are chosen simultaneously. Second, it separates rating noise $\sigma_{Y}$, rating-innovation ambiguity $\phi_{4}$, and systematic rating-bias misspecification $g_{true}$. Third, shared market innovations generate the mixed term $a(u{)}^{⊤}\ell_{t}V_{xz}$ and a filtering-hedging component in stock demand. Fourth, the practical constraint $q_{t}\in [0,1]$ is incorporated directly into the HJBI and conditional verification result. The standard filtering and pointwise optimization arguments are retained only to establish notation and validity; their detailed proofs are collected in Appendix A. Numerical experiments use illustrative rather than calibrated parameters and are interpreted as mechanism and stress evidence.

The remainder of the paper is organized as follows. Section 2 provides preliminaries and distinguishes standard tools from model-specific contributions. Section 3 formulates the hidden-state, observation, insurance, and ambiguity components. Sections 4 and 5 derive the separated HJBI, controls, coefficient system, and verification result. Section 6 reports the numerical experiments, and Section 7 concludes. Technical proofs are collected in Appendix A.

## 2 Preliminaries and contribution map

This section provides a compact guide for readers who are less familiar with partial-information robust control. Three standard tools are used. First, a Kalman–Bucy filter replaces the unobserved green-quality factor by its conditional mean and variance under the physical reference measure. Second, Girsanov drift distortions define nearby models for robust evaluation. Third, the Hamilton–Jacobi–Bellman–Isaacs (HJBI) equation optimizes the insurer’s controls against those distortions. These tools are classical; the detailed standard arguments are moved to Appendix A.

The model-specific element is the way the four innovation channels enter wealth and beliefs. Box 1 summarizes this structure without altering the numbering of the manuscript’s numerical tables. Because the first two innovations are both return shocks and learning signals, their distortions cannot be labeled as purely financial or purely informational. This is an intentional implication of joint learning, not a complete separation of market and information-quality uncertainty.

**Box 1. Economic interpretation of the four robust-control distortions. The display distinguishes mathematical role from economic motivation.**

**Table:** 

Component	Innovation channel	Wealth effect	Belief effect
$\phi_{1}$	Green-stock return	Direct	Direct
$\phi_{2}$	Orthogonalized brown return	Direct	Direct
$\phi_{3}$	Underwriting shock	Direct	None
$\phi_{4}$	ESG-rating innovation	None	Direct

*Interpretation:* $\phi_{1}$ and $\phi_{2}$ combine market and information-model ambiguity because returns update beliefs. $\phi_{3}$ represents underwriting ambiguity. $\phi_{4}$ represents rating-innovation ambiguity. The separate stress parameter $g_{true}$ is an actual bias in the simulated rating process and is not a robust-control distortion.

Within this structure, the new analytical content is the mixed wealth-belief derivative, its filtering-hedging demand, the constrained retention rule inside the robust HJBI, and the conditional verification of the semi-closed-form candidate. The Kalman–Bucy formula, measure change, and unconstrained quadratic completion are standard ingredients specialized to this model.

## 3 Model formulation

### 3.1 Hidden green quality and joint observations

Fix a finite horizon *T* > 0. Let


$$\begin{array}{c} (\Omega ,ℱ,F,\mathbb{P}),\;F=\{{ℱ}_{t}{\}}_{0\leq t\leq T}, \end{array}$$ (3.1)

be a filtered probability space satisfying the usual conditions. Throughout, ℙ is the physical reference probability measure, not a risk-neutral pricing measure. The hidden true green quality $Z=\{Z_{t}{\}}_{0\leq t\leq T}$ follows the Ornstein–Uhlenbeck process


$$\begin{array}{c} \;dZ_{t}=\alpha_{Z}(\bar{Z}-Z_{t})\;dt+\sigma_{Z}\;dW_{0}(t),\;Z_{0}\sim N(m_{0}^{Z},P_{0}), \end{array}$$ (3.2)

where $\alpha_{Z}>0$, $\sigma_{Z}>0$, *P*_0_ > 0, and $\bar{Z},m_{0}^{Z}\in \mathbb{R}$. The Brownian motion *W*_0_ drives the latent green-quality factor and is independent of the observation and underwriting noises introduced below.

The latent-state specification captures the fact that the insurer does not observe the firm’s true green quality directly. Modeling this hidden quality as a mean-reverting Gaussian factor keeps the system within the linear-Gaussian partial-observation framework, for which finite-dimensional Kalman–Bucy filtering is available [33,35]. In the same spirit, ESG ratings are treated as noisy signals rather than error-free observations of sustainability, consistent with the documented divergence and uncertainty in ESG ratings [10–12,14]. Stock returns may also contain information about green quality, so the model allows green returns, orthogonalized brown returns, and the ESG rating signal to form a joint observation system. Thus $Y_{t}$ should be read as a cumulative ESG-rating information flow rather than a perfectly observed sustainability score.

Let $W_{1},W_{2},W_{3},W_{4}$ be independent Brownian motions under ℙ, and independent of *W*_0_. The roles are fixed throughout: *W*_1_ drives the green-return innovation, *W*_2_ the orthogonalized brown-return innovation, *W*_3_ underwriting risk, and *W*_4_ the ESG-rating innovation. The green and brown stock excess returns are


$$\begin{array}{cc} \;d{\bar{R}}_{t}^{g} & =(\mu_{g}^{0}+\beta_{g}Z_{t})\;dt+\sigma_{g}\;dW_{1}(t), \end{array}$$ (3.3)

$$\begin{array}{cc} \;d{\bar{R}}_{t}^{b} & =(\mu_{b}^{0}-\beta_{b}Z_{t})\;dt+\sigma_{b}\{\rho \;dW_{1}(t)+s\;dW_{2}(t)\},\;s=\sqrt{1-\rho^{2}}, \end{array}$$ (3.4)

where $\sigma_{g},\sigma_{b}>0$, ρ∈(−1,1), and $\beta_{g},\beta_{b}>0$. The ESG rating signal is


$$\begin{array}{c} \;dY_{t}=(Z_{t}+\delta_{0})\;dt+\sigma_{Y}\;dW_{4}(t),\;\sigma_{Y}>0,\;\delta_{0}\geq 0. \end{array}$$ (3.5)

The rating noise parameter $\sigma_{Y}$ measures rating informativeness, while $\delta_{0}$ is the average bias already incorporated in the reference model. Thus $\sigma_{Y}$ represents imprecision, $\delta_{0}$ a known reference offset, $\phi_{4}$ ambiguity about the rating-innovation drift, and $g_{true}$ an omitted systematic bias used only in the misspecification experiment. None of these quantities alone is direct evidence of firm-level greenwashing. Rating-disagreement studies motivate $\sigma_{Y}$ [10–12,14]; greenwashing and selective-disclosure studies motivate examining rating credibility and systematic bias [18–21].

Because the green and brown stock return noises are correlated, it is convenient to orthogonalize the brown return observation:


$$\begin{array}{c} \;d{\bar{R}}_{t}^{⟂}=\;d{\bar{R}}_{t}^{b}-\rho \frac{\sigma_{b}}{\sigma_{g}}\;d{\bar{R}}_{t}^{g}. \end{array}$$ (3.6)

Then


$$\begin{array}{c} \;d{\bar{R}}_{t}^{⟂}=(\mu_{⟂}^{0}+\beta_{⟂}Z_{t})\;dt+\sigma_{b}s\;\;dW_{2}(t), \end{array}$$ (3.7)

where


$$\begin{array}{c} \mu_{⟂}^{0}=\mu_{b}^{0}-\rho \frac{\sigma_{b}}{\sigma_{g}}\mu_{g}^{0},\;\beta_{⟂}=-\beta_{b}-\rho \frac{\sigma_{b}}{\sigma_{g}}\beta_{g}. \end{array}$$ (3.8)

The three-dimensional observable vector is denoted by


$$\begin{array}{c} Y_{t}=({\bar{R}}_{t}^{g},{\bar{R}}_{t}^{⟂},Y_{t}{)}^{⊤}. \end{array}$$ (3.9)

Using $\left({\bar{R}}^{g},{\bar{R}}^{⟂},Y\right)$ is equivalent to using the original observations $\left({\bar{R}}^{g},{\bar{R}}^{b},Y\right)$, because (3.6) is an invertible linear transformation once ${\bar{R}}^{g}$ is observed. The orthogonalized system only makes the Kalman–Bucy formula diagonal in the observation noise.

The market-rating observation filtration is


$$\begin{array}{c} F^{Y}=\{{ℱ}_{t}^{Y}{\}}_{0\leq t\leq T},\;{ℱ}_{t}^{Y}=\sigma \{{\bar{R}}_{s}^{g},{\bar{R}}_{s}^{⟂},Y_{s}:0\leq s\leq t{\}}^{\mathbb{P}\text{-aug}}. \end{array}$$ (3.10)

The insurer’s control filtration $F^{O}$ contains $F^{Y}$ and the internally observed wealth and underwriting records. Since the underwriting noise is independent of $Z_{t}$, enlarging $F^{Y}$ by underwriting information does not change the conditional law of $Z_{t}$. The reference conditional mean and conditional variance of $Z_{t}$ are


$$\begin{array}{c} {\hat{Z}}_{t}={𝔼}_{\mathbb{P}}[Z_{t}|{ℱ}_{t}^{Y}],\;P_{t}={𝔼}_{\mathbb{P}}[(Z_{t}-{\hat{Z}}_{t}{)}^{2}|{ℱ}_{t}^{Y}]. \end{array}$$ (3.11)

### 3.2 Kalman–Bucy filter with stock-return learning

Define the normalized innovation processes


$$\begin{array}{cc} \;d{\tilde{W}}_{1}(t) & =\frac{\;d{\bar{R}}_{t}^{g}-(\mu_{g}^{0}+\beta_{g}{\hat{Z}}_{t})\;dt}{\sigma_{g}}, \end{array}$$ (3.12)

$$\begin{array}{cc} \;d{\tilde{W}}_{2}(t) & =\frac{\;d{\bar{R}}_{t}^{⟂}-(\mu_{⟂}^{0}+\beta_{⟂}{\hat{Z}}_{t})\;dt}{\sigma_{b}s}, \end{array}$$ (3.13)

$$\begin{array}{cc} \;d{\tilde{W}}_{4}(t) & =\frac{\;dY_{t}-({\hat{Z}}_{t}+\delta_{0})\;dt}{\sigma_{Y}}. \end{array}$$ (3.14)

The information intensity of the joint observation system is


$$\begin{array}{c} \chi =\frac{\beta_{g}^{2}}{\sigma_{g}^{2}}+\frac{\beta_{⟂}^{2}}{\sigma_{b}^{2}s^{2}}+\frac{1}{\sigma_{Y}^{2}}. \end{array}$$ (3.15)

Since the state equation and the orthogonalized observation equations form a scalar linear-Gaussian state-observation system, the following filter is a direct application of the classical Kalman–Bucy filtering theorem; see, for example, Bensoussan [33] and Bishop and Del Moral [35].

**Lemma 3.1** (Joint Kalman–Bucy filter). *The process* $\left({\tilde{W}}_{1},{\tilde{W}}_{2},{\tilde{W}}_{4}\right)$ *is a three-dimensional* $F^{Y}$*-Brownian motion under* ℙ*. The conditional mean satisfies*


$$\begin{array}{cc} \;d{\hat{Z}}_{t} & =\alpha_{Z}(\bar{Z}-{\hat{Z}}_{t})\;dt+P_{t}\left(\frac{\beta_{g}}{\sigma_{g}}\;d{\tilde{W}}_{1}(t)+\frac{\beta_{⟂}}{\sigma_{b}s}\;d{\tilde{W}}_{2}(t)+\frac{1}{\sigma_{Y}}\;d{\tilde{W}}_{4}(t)\right), \end{array}$$ (3.16)

$$\begin{array}{cc} {\dot{P}}_{t} & =\sigma_{Z}^{2}-2\alpha_{Z}P_{t}-\chi P_{t}^{2},\;P_{0}>0. \end{array}$$ (3.17)

*Moreover,* $P_{t}$ *is deterministic, strictly positive, bounded, and unique on [0,T].*

*Proof.* The derivation is provided in Appendix A, subsection “Joint Kalman–Bucy filter.”

### 3.3 Financial market, insurance risk, and wealth

The risk-free asset satisfies


$$\begin{array}{c} \;dS_{0}(t)=rS_{0}(t)\;dt. \end{array}$$ (3.18)

Under the separated observation filtration, the stock prices have the semimartingale decompositions


$$\begin{array}{cc} \frac{\;dS_{g}(t)}{S_{g}(t)} & =(r+\mu_{g}({\hat{Z}}_{t}))\;dt+\sigma_{g}\;d{\tilde{W}}_{1}(t), \end{array}$$ (3.19)

$$\begin{array}{cc} \frac{\;dS_{b}(t)}{S_{b}(t)} & =(r+\mu_{b}({\hat{Z}}_{t}))\;dt+\sigma_{b}\{\rho \;d{\tilde{W}}_{1}(t)+s\;d{\tilde{W}}_{2}(t)\}, \end{array}$$ (3.20)

where


$$\begin{array}{c} \mu_{g}(z)=\mu_{g}^{0}+\beta_{g}z,\;\mu_{b}(z)=\mu_{b}^{0}-\beta_{b}z. \end{array}$$ (3.21)

The diffusion approximation with proportional reinsurance is standard in continuous-time insurance models with reinsurance and investment, including partial-information and robust variants [27–31]. The retention level $q_{t}$ controls the retained underwriting volatility and the reinsurance premium cost, while the investment controls determine exposures to green and brown stock risks. Accordingly, the insurance surplus after proportional reinsurance is


$$\begin{array}{c} \;dR_{t}^{q}=c[\eta -\vartheta (1-q_{t})]\;dt+bq_{t}\;dW_{3}(t),\;c>0,\;b>0,\;\vartheta \geq \eta >0. \end{array}$$ (22)

Throughout the paper, $X_{t}$ denotes discounted wealth. The insurance premium scale *c*, the safety loadings, and the underwriting diffusion coefficient *b* are understood in discounted units; equivalently, deterministic discount factors have been absorbed into these parameters.

Let $u_{t}=(\pi_{g}(t),\pi_{b}(t),q_{t}{)}^{⊤}$, where $\pi_{g},\pi_{b}$ are discounted investment amounts in the green and brown stocks and $q_{t}$ is the retention level. Define


$$\begin{array}{c} {\tilde{W}}_{t}=({\tilde{W}}_{1}(t),{\tilde{W}}_{2}(t),W_{3}(t),{\tilde{W}}_{4}(t){)}^{⊤}, \end{array}$$ (3.23)

$$\begin{array}{c} m(z)=(\begin{array}{c} \mu_{g}^{0}+\beta_{g}z\\ \mu_{b}^{0}-\beta_{b}z\\ c\vartheta \end{array}),\;\Sigma =(\begin{array}{ccc} \sigma_{g} & \rho \sigma_{b} & 0\\ 0 & s\sigma_{b} & 0\\ 0 & 0 & b\\ 0 & 0 & 0 \end{array}),\;a(u)=\Sigma u. \end{array}$$ (3.24)

The discounted wealth process is


$$\begin{array}{c} \;dX_{t}=\{m({\hat{Z}}_{t}{)}^{⊤}u_{t}+c(\eta -\vartheta )\}\;dt+a(u_{t}{)}^{⊤}\;d{\tilde{W}}_{t}. \end{array}$$ (3.25)

The use of discounted wealth is only a normalization; it does not change the control tradeoff between underwriting retention and risky investment.

The filter equation (3.16) can be written as


$$\begin{array}{c} \;d{\hat{Z}}_{t}=\alpha_{Z}(\bar{Z}-{\hat{Z}}_{t})\;dt+\ell_{t}^{⊤}\;d{\tilde{W}}_{t}, \end{array}$$ (3.26)

where


$$\begin{array}{c} \ell_{t}={\left(P_{t}\frac{\beta_{g}}{\sigma_{g}},P_{t}\frac{\beta_{⟂}}{\sigma_{b}s},0,P_{t}\frac{1}{\sigma_{Y}}\right)}^{⊤}. \end{array}$$ (3.27)

The crucial point is that both $X_{t}$ and ${\hat{Z}}_{t}$ are driven by the stock-return innovations ${\tilde{W}}_{1},{\tilde{W}}_{2}$. Therefore the HJBI equation must contain the mixed derivative $a(u{)}^{⊤}\ell_{t}V_{xz}$. This feature is specific to the present joint-learning formulation: market innovations are simultaneously payoff shocks and information shocks. It is precisely this feature that later generates the mixed derivative term in the separated HJBI equation.

*Remark 3.2* (Underwriting noise and observation). The Brownian motion *W*_3_ is the underwriting risk innovation in the wealth process. It is independent of $Z_{t}$, its driver W_0_, and the market-rating observation noises, and therefore it carries no information about hidden green quality. The Kalman–Bucy filter is consequently determined only by $\left({\bar{R}}^{g},{\bar{R}}^{⟂},Y\right)$. In the separated Brownian vector $\tilde{W}$, *W*_3_ is retained only to generate the underwriting martingale term in wealth. If $q_{t}=0$, the insurer need not recover *W*_3_ from the surplus path for filtering purposes; the belief dynamics remain unchanged.

### 3.4 Robust ambiguity and admissibility

To represent model ambiguity, we use the standard robust-control device of evaluating nearby models through drift distortions of Brownian innovations, penalized by a quadratic relative-entropy-type term [22,23].

A model distortion is a progressively measurable process


$$\begin{array}{c} \phi_{t}=(\phi_{1}(t),\phi_{2}(t),\phi_{3}(t),\phi_{4}(t){)}^{⊤}. \end{array}$$ (3.28)

The distorted measure ${\mathbb{Q}}^{\phi }$ is defined by


$$\begin{array}{c} \frac{\;d{\mathbb{Q}}^{\phi }}{\;d\mathbb{P}}{|}_{{ℱ}_{T}^{O}}=E_{T}\left(-\int_{0}^{\cdot }\phi_{s}^{⊤}\;d{\tilde{W}}_{s}\right), \end{array}$$ (3.29)

when the stochastic exponential is a true martingale. Under ${\mathbb{Q}}^{\phi }$,


$$\begin{array}{c} \;d{\tilde{W}}_{i}^{\phi }(t)=\;d{\tilde{W}}_{i}(t)+\phi_{i}(t)\;dt,\;i=1,2,3,4, \end{array}$$ (3.30)

are Brownian motions. The components have different propagation paths. Distortions $\phi_{1}$ and $\phi_{2}$ alter stock-return innovations, so they affect both contemporaneous wealth and belief updating. The underwriting distortion $\phi_{3}$ affects wealth but not beliefs. The rating distortion $\phi_{4}$ affects beliefs but has no direct wealth-diffusion loading because the fourth row of Σ is zero. Accordingly, $\phi_{1}$ and $\phi_{2}$ combine financial and informational ambiguity; the framework does not fully disentangle these two roles. We call $\phi_{4}$ rating-innovation ambiguity. Greenwashing and selective-disclosure studies motivate the credibility concern [18,20,21], while robust control supplies the mathematical drift-distortion device [22,23].

The separated robust dynamics are


$$\begin{array}{cc} \;dX_{t} & =\{m({\hat{Z}}_{t}{)}^{⊤}u_{t}+c(\eta -\vartheta )-a(u_{t}{)}^{⊤}\phi_{t}\}\;dt+a(u_{t}{)}^{⊤}\;d{\tilde{W}}_{t}^{\phi }, \end{array}$$ (3.31)

$$\begin{array}{cc} \;d{\hat{Z}}_{t} & =\{\alpha_{Z}(\bar{Z}-{\hat{Z}}_{t})-\ell_{t}^{⊤}\phi_{t}\}\;dt+\ell_{t}^{⊤}\;d{\tilde{W}}_{t}^{\phi }. \end{array}$$ (3.32)

*Remark 3.3* (Reference filter and robust distortion). The belief state ${\hat{Z}}_{t}$ is the reference filter, namely ${\hat{Z}}_{t}={𝔼}_{\mathbb{P}}[Z_{t}|{ℱ}_{t}^{Y}]$. Under a distorted measure ${\mathbb{Q}}^{\phi }$, this paper does not redefine the belief state as ${𝔼}_{{\mathbb{Q}}^{\phi }}[Z_{t}|{ℱ}_{t}^{Y}]$. The decision maker filters under the approximating model and evaluates robustness through penalized drift distortions of the separated innovations [22,23,33,34]. Thus $\phi_{4}$ is observation-model ambiguity in the ESG-rating channel, while $\phi_{1}$ and $\phi_{2}$ affect both wealth risk and belief dynamics. This convention and its limits are stated once here and used throughout.

Let


$$\begin{array}{c} U(x)=-e^{-\gamma x},\;\gamma >0, \end{array}$$ (3.33)

and


$$\begin{array}{c} \Gamma =diag(\kappa_{1},\kappa_{2},\kappa_{3},\kappa_{4}),\;\kappa_{i}>0. \end{array}$$ (3.34)

The value-dependent penalty is


$$\begin{array}{c} -\frac{1}{2}V(t,x,z)\phi^{⊤}\Gamma \phi . \end{array}$$ (3.35)

Since *V* < 0, this term makes the inner minimization strictly convex in ϕ. The value-dependent penalty preserves the exponential-quadratic structure under CARA utility and yields a tractable pointwise minimization over distortions [22,23].

The admissible controls are not defined through affine Markov feedbacks. For an initial state (*t*,*x*,*z*), let $A_{0}(t,x,z)$ be the base class of observation-filtration progressively measurable controls


$$\begin{array}{c} u_{s}=(\pi_{g}(s),\pi_{b}(s),q_{s}{)}^{⊤},\;t\leq s\leq T, \end{array}$$ (3.36)

such that $q_{s}\in [0,1]$ a.s. for all *s* and


$$\begin{array}{c} 𝔼\int_{t}^{T}\left(\pi_{g}(s{)}^{2}+\pi_{b}(s{)}^{2}+q_{s}^{2}\right)\;ds<\infty . \end{array}$$ (3.37)

For a given control $u\in A_{0}(t,x,z)$, let $\Theta_{0}(t,x,z;u)$ be the base class of progressively measurable distortions $\phi =(\phi_{1},\phi_{2},\phi_{3},\phi_{4}{)}^{⊤}$ for which the stochastic exponential


$$\begin{array}{c} E_{s}\left(-\int_{t}^{\cdot }\phi_{r}^{⊤}\;d{\tilde{W}}_{r}\right),\;t\leq s\leq T, \end{array}$$ (3.38)

is a true martingale, the separated state equation (3.31)–(3.32) has a unique strong solution under ${\mathbb{Q}}^{\phi }$, and


$$\begin{array}{c} {𝔼}^{{\mathbb{Q}}^{\phi }}\int_{t}^{T}\phi_{s}^{⊤}\Gamma \phi_{s}\;ds<\infty . \end{array}$$ (3.39)

Because the stock controls are unconstrained and the value function has exponential growth, admissibility cannot be reduced to square-integrability alone. The true-martingale, finite-distortion, and uniform-integrability requirements are imposed to make the Girsanov change of measure and verification argument well defined [33,34].

**Assumption 3.4** (Admissibility and integrability). *For the verification argument associated with a smooth candidate V, the admissible distortion class* $\Theta^{V}(t,x,z;u)\subseteq \Theta_{0}(t,x,z;u)$ *consists of distortions satisfying the following explicit requirements.*

1. *The Girsanov density (3.38) is a true martingale, and the finite-distortion condition (3.39) holds.*
2. *The terminal value and value-dependent running penalty are integrable under* ${\mathbb{Q}}^{\phi }$:

$$\begin{array}{c} {𝔼}^{{\mathbb{Q}}^{\phi }}\left[|U(X_{T})|+\int_{t}^{T}|V(s,X_{s},{\hat{Z}}_{s})|\;\phi_{s}^{⊤}\Gamma \phi_{s}\;ds\right]<\infty . \end{array}$$ (3.40)

3. *For every stopping time* τ≤T*, the family*
  $$\begin{array}{c} \left\{V(\tau ,X_{\tau },{\hat{Z}}_{\tau })+\int_{t}^{\tau }\left(-\frac{1}{2}V(s,X_{s},{\hat{Z}}_{s})\phi_{s}^{⊤}\Gamma \phi_{s}\right)\;ds:\tau \leq T\right\} \end{array}$$ (3.41)
  * is uniformly integrable under* ${\mathbb{Q}}^{\phi }$*. The stochastic integrals generated by Ito’s formula are local martingales whose localized expectations vanish in the limit.*

*For a control* $u\in A_{0}(t,x,z)$*, let* $\phi^{u}$ *denote the pointwise Hamiltonian minimizer from Proposition 4.2, when evaluated along u. The verification domain is*


$$\begin{array}{c} A^{V}(t,x,z)=\{u\in A_{0}(t,x,z):\phi^{u}\in \Theta^{V}(t,x,z;u)\}. \end{array}$$ (3.42)

*Thus admissibility is imposed through well-posedness, true-martingale, finite-distortion, and class-D requirements; it is not an affine Markov restriction.*

The robust objective over the verification domain is


$$\begin{array}{cc} J(t,x,z;u,\phi ) & ={𝔼}_{t,x,z}^{{\mathbb{Q}}^{\phi }}[U(X_{T})+\int_{t}^{T}\left(-\frac{1}{2}V(s,X_{s},{\hat{Z}}_{s})\phi_{s}^{⊤}\Gamma \phi_{s}\right)\;ds], \end{array}$$ (3.43)

$$\begin{array}{cc} V(t,x,z) & =\sup_{u\in A^{V}(t,x,z)}\inf_{\phi \in \Theta^{V}(t,x,z;u)}J(t,x,z;u,\phi ). \end{array}$$ (3.44)

The affine Markov class used later to obtain explicit formulas is a sufficient subclass, not the definition of admissibility. For a fixed $L_{u}<\infty$, define


$$\begin{array}{c} {𝒰}_{aff}(L_{u})=\left\{\begin{array}{c} u(t,z)=u_{0}(t)+U_{1}(t)z,\\ u_{0}\in C([0,T];{\mathbb{R}}^{3}),\;U_{1}\in C([0,T];{\mathbb{R}}^{3\times 1}),\\ \sup_{0\leq t\leq T}(\|u_{0}(t)\|+\|U_{1}(t)\|)\leq L_{u} \end{array}\right\}. \end{array}$$ (3.45)

Here


$$\begin{array}{c} u_{0}(t)=(\begin{array}{c} u_{g,0}(t)\\ u_{b,0}(t)\\ u_{q,0}(t) \end{array}),\;U_{1}(t)=(\begin{array}{c} u_{g,1}(t)\\ u_{b,1}(t)\\ u_{q,1}(t) \end{array}). \end{array}$$ (3.46)

The associated integrable affine distortion class is


$$\begin{array}{c} \Theta_{aff}^{int}=\left\{\begin{array}{c} \phi (t,z)=p(t)+R(t)z,\\ p\in C([0,T];{\mathbb{R}}^{4}),\;R\in C([0,T];{\mathbb{R}}^{4\times 1}),\\ p,R\text{ are bounded, and Condition (I) in Lemma 5.7 holds} \end{array}\right\}. \end{array}$$ (3.47)

The superscript “int” indicates integrable affine distortions. In the verification argument, Condition (I) is checked relative to the affine feedback paired with ϕ. It implies


$$\begin{array}{c} {𝔼}_{\mathbb{P}}\exp\left(\frac{1}{2}\int_{0}^{T}\|\phi_{t}{\|}^{2}\;dt\right)<\infty . \end{array}$$ (3.48)

The bound $L_{u}$ is used only to give a directly checkable sufficient condition for the explicit affine candidate. It is not part of the main admissible-control definition.

## 4 Separated HJBI equation

### 4.1 Separation principle

**Proposition 4.1** (Reference-filter separation under joint observations). *Under the reference model* ℙ*, the market-rating observations* $\left({\bar{R}}^{g},{\bar{R}}^{b},Y\right)$ *generate the finite-dimensional Kalman–Bucy filter* ${\hat{Z}}_{t}={𝔼}_{\mathbb{P}}[Z_{t}|{ℱ}_{t}^{Y}]$*. The robust control problem in this paper is formulated on the reference-filter state* $\left(X_{t},{\hat{Z}}_{t}\right)$*, with distorted separated dynamics (3.31)–(3.32). The filter is generated by the reference innovations* $\left({\tilde{W}}_{1},{\tilde{W}}_{2},{\tilde{W}}_{4}\right)$*; the underwriting shock W*_*3*_ *enters only as the martingale shock in wealth and does not update the belief about* $Z_{t}$*.*

*Proof.* The derivation is provided in Appendix A, subsection “Reference-filter separation under joint observations.”

### 4.2 HJBI equation and generator

Let $V\in C^{1,2,2}([0,T)\times {\mathbb{R}}^{2})$. For fixed *u* and ϕ, the generator of $\left(X_{t},{\hat{Z}}_{t}\right)$ under ${\mathbb{Q}}^{\phi }$ is


$$\begin{array}{cc} L^{u,\phi }V & =[m(z{)}^{⊤}u+c(\eta -\vartheta )-a(u{)}^{⊤}\phi ]V_{x}+[\alpha_{Z}(\bar{Z}-z)-\ell_{t}^{⊤}\phi ]V_{z}\\ & \;+\frac{1}{2}a(u{)}^{⊤}a(u)V_{xx}+a(u{)}^{⊤}\ell_{t}V_{xz}+\frac{1}{2}\ell_{t}^{⊤}\ell_{t}V_{zz}. \end{array}$$ (4.1)

The mixed term $a(u{)}^{⊤}\ell_{t}V_{xz}$ is present because stock-return innovations affect both wealth and the belief state. The pointwise HJBI equation with constrained proportional retention is


$$\begin{array}{c} 0=\sup_{\pi_{g},\pi_{b}\in \mathbb{R},\;q\in [0,1]}\inf_{\phi \in {\mathbb{R}}^{4}}\left\{V_{t}+L^{u,\phi }V-\frac{1}{2}V\phi^{⊤}\Gamma \phi \right\},\;V(T,x,z)=-e^{-\gamma x}. \end{array}$$ (4.2)

The stock positions remain unconstrained in the main model, while q∈[0,1] is imposed directly in the Hamiltonian and in the verification result.

### 4.3 Worst-case distortion

**Proposition 4.2** (Pointwise worst-case distortion). *Assume V < 0 and*


$$\begin{array}{c} V(t,x,z)=-\exp[-\gamma x+F(t,z)]. \end{array}$$ (4.3)

*For a fixed control u, the inner minimizer in (4.2) is unique and is given by*


$$\begin{array}{c} \phi_{i}^{*}(t,z,u)=\frac{\gamma a_{i}(u)-\ell_{i,t}F_{z}(t,z)}{\kappa_{i}},\;i=1,2,3,4. \end{array}$$ (4.4)

*In particular,*


$$\begin{array}{c} \phi_{3}^{*}=\frac{\gamma bq}{\kappa_{3}},\;\phi_{4}^{*}=-\frac{\ell_{4,t}F_{z}(t,z)}{\kappa_{4}}. \end{array}$$ (4.5)

*Proof.* The derivation is provided in Appendix A, subsection “Pointwise worst-case distortion.”

## 5 Semi-closed-form stock strategy, constrained retention, and value function

### 5.1 Transformed HJBI

Set


$$\begin{array}{c} V(t,x,z)=-\exp[-\gamma x+F(t,z)],\;F(T,z)=0. \end{array}$$ (5.1)

In addition to (A.6),


$$\begin{array}{c} V_{xx}=\gamma^{2}V,\;V_{zz}=V(F_{zz}+F_{z}^{2}). \end{array}$$ (5.2)

Define


$$\begin{array}{cc} D_{\kappa } & =diag\left(1+\frac{1}{\kappa_{1}},1+\frac{1}{\kappa_{2}},1+\frac{1}{\kappa_{3}},1+\frac{1}{\kappa_{4}}\right), \end{array}$$ (5.4)

$$\begin{array}{cc} M & =\Sigma^{⊤}D_{\kappa }\Sigma ,\;\Lambda_{t}=\Sigma^{⊤}D_{\kappa }\ell_{t}, \end{array}$$ (5.4)

$$\begin{array}{cc} \nu_{t} & =\ell_{t}^{⊤}D_{\kappa }\ell_{t},\;\zeta_{t}=\ell_{t}^{⊤}\ell_{t}. \end{array}$$ (5.5)

Let $\Sigma_{\pi }$ be the first two columns of Σ, and define


$$\begin{array}{c} M_{\pi }=\Sigma_{\pi }^{⊤}D_{\kappa }\Sigma_{\pi },\;\Lambda_{\pi ,t}=\Sigma_{\pi }^{⊤}D_{\kappa }\ell_{t}. \end{array}$$ (5.6)

Set


$$\begin{array}{c} d_{3}=1+\frac{1}{\kappa_{3}},\;q^{unc}=\frac{c\vartheta }{\gamma d_{3}b^{2}},\;q^{*}=\Pi_{\left[0,1\right]}(q^{unc}), \end{array}$$ (5.7)

and


$$\begin{array}{c} G_{q}^{*}=\min_{0\leq q\leq 1}\left\{\frac{1}{2}\gamma^{2}d_{3}b^{2}q^{2}-\gamma c\vartheta q\right\}. \end{array}$$ (5.8)

Equivalently,


$$\begin{array}{c} G_{q}^{*}=\left\{ \begin{array}{cc} 0, & q^{unc}\leq 0,\\ {}[0.25em]-\frac{c^{2}\vartheta^{2}}{2d_{3}b^{2}}, & 0<q^{unc}<1,\\ {}[0.75em]\frac{1}{2}\gamma^{2}d_{3}b^{2}-\gamma c\vartheta , & q^{unc}\geq 1. \end{array}\right. \end{array}$$ (5.9)

**Proposition 5.1** (Reduced PDE and constrained retention). *The transformed HJBI for F is*


$$\begin{array}{cc} 0 & =F_{t}-\gamma c(\eta -\vartheta )+\alpha_{Z}(\bar{Z}-z)F_{z}+\frac{1}{2}\zeta_{t}F_{zz}+\frac{1}{2}\nu_{t}F_{z}^{2}+G_{q}^{*}\\ & \;-\frac{1}{2}\{m_{\pi }(z)+F_{z}\Lambda_{\pi ,t}{\}}^{⊤}M_{\pi }^{-1}\{m_{\pi }(z)+F_{z}\Lambda_{\pi ,t}\}. \end{array}$$ (5.10)

*The stock feedback is*


$$\begin{array}{c} \pi^{*}(t,z)=\frac{1}{\gamma }M_{\pi }^{-1}\{m_{\pi }(z)+F_{z}(t,z)\Lambda_{\pi ,t}\}, \end{array}$$ (5.11)

*where* $m_{\pi }(z)=(\mu_{g}^{0}+\beta_{g}z,\mu_{b}^{0}-\beta_{b}z{)}^{⊤}$*. Equivalently,*


$$\begin{array}{c} \pi^{*}(t,z)=\frac{1}{\gamma }M_{\pi }^{-1}m_{\pi }(z)+\frac{1}{\gamma }M_{\pi }^{-1}\Lambda_{\pi ,t}F_{z}(t,z). \end{array}$$ (5.12)

*The first term is the myopic investment demand. The second term is the filtering hedging demand generated by learning hidden green quality from stock returns. The proportional retention is the constrained optimizer* $q^{*}$ *in (5.7).*


$$\begin{array}{c} u^{*}(t,z)=(\pi_{g}^{*}(t,z),\pi_{b}^{*}(t,z),q^{*}{)}^{⊤}. \end{array}$$ (5.13)

*Proof.* The derivation is provided in Appendix A, subsection “Reduced HJBI equation and constrained retention.”

**Lemma 5.2 (Positive definiteness and projection inequality).** *The matrices M and* $M_{\pi }$ *are positive definite. Moreover,*


$$\begin{array}{c} \Lambda_{t}^{⊤}M^{-1}\Lambda_{t}\leq \nu_{t},\;0\leq t\leq T. \end{array}$$ (5.14)

*and*


$$\begin{array}{c} \Lambda_{\pi ,t}^{⊤}M_{\pi }^{-1}\Lambda_{\pi ,t}\leq \nu_{t},\;0\leq t\leq T. \end{array}$$ (5.15)

*Proof.* The derivation is provided in Appendix A, subsection “Positive definiteness and projection inequality.”

### 5.2 Riccati-type system

The PDE (5.10) preserves quadratic dependence on the filtered state. Let


$$\begin{array}{c} F(t,z)=A_{2}(t)z^{2}+B_{1}(t)z+C(t),\;A_{2}(T)=B_{1}(T)=C(T)=0. \end{array}$$ (5.16)

Write the stock drift vector as


$$\begin{array}{c} m_{\pi }(z)=m_{\pi ,0}+m_{\pi ,1}z,\;m_{\pi ,0}=(\begin{array}{c} \mu_{g}^{0}\\ \mu_{b}^{0} \end{array}),\;m_{\pi ,1}=(\begin{array}{c} \beta_{g}\\ -\beta_{b} \end{array}). \end{array}$$ (5.17)

Since $F_{z}=2A_{2}z+B_{1}$, define


$$\begin{array}{c} n_{0}(t)=m_{\pi ,0}+B_{1}(t)\Lambda_{\pi ,t},\;n_{1}(t)=m_{\pi ,1}+2A_{2}(t)\Lambda_{\pi ,t}. \end{array}$$ (5.18)

Then


$$\begin{array}{c} m_{\pi }(z)+F_{z}(t,z)\Lambda_{\pi ,t}=n_{0}(t)+n_{1}(t)z, \end{array}$$ (5.19)

which is affine in *z*. Therefore the PDE contains only powers *z*^2^,*z*,1.

**Proposition 5.3** (Coefficient ODEs). *The coefficients in (5.16) solve*


$$\begin{array}{cc} 0 & ={\dot{A}}_{2}-2\alpha_{Z}A_{2}+2\nu_{t}A_{2}^{2}-\frac{1}{2}n_{1}(t{)}^{⊤}M_{\pi }^{-1}n_{1}(t), \end{array}$$ (5.20)

$$\begin{array}{cc} 0 & ={\dot{B}}_{1}+\alpha_{Z}(2\bar{Z}A_{2}-B_{1})+2\nu_{t}A_{2}B_{1}-n_{1}(t{)}^{⊤}M_{\pi }^{-1}n_{0}(t), \end{array}$$ (5.21)

$$\begin{array}{cc} 0 & =\dot{C}-\gamma c(\eta -\vartheta )+\alpha_{Z}\bar{Z}B_{1}+\zeta_{t}A_{2}+\frac{1}{2}\nu_{t}B_{1}^{2}-\frac{1}{2}n_{0}(t{)}^{⊤}M_{\pi }^{-1}n_{0}(t)+G_{q}^{*}, \end{array}$$ (5.22)

*with terminal conditions* $A_{2}(T)=B_{1}(T)=C(T)=0$.

*Proof.* The derivation is provided in Appendix A, subsection “Coefficient ODE system.”

**Proposition 5.4** (Global solvability of the coefficient system). *Under the standing parameter assumptions*


$$\begin{array}{c} \alpha_{Z},\sigma_{Z},\sigma_{g},\sigma_{b},\sigma_{Y},b,c,\eta ,\vartheta ,\gamma ,\kappa_{i}>0,\;\rho \in (-1,1), \end{array}$$

*the system (5.20)–(5.22) has a unique finite solution on [0,T].*

*Proof.* The derivation is provided in Appendix A, subsection “Global solvability of the coefficient system.”

**Proposition 5.5** (Affine closure of feedbacks and distortions). *Let* $u\in {𝒰}_{aff}(L_{u})$*, so* $u(t,z)=u_{0}(t)+U_{1}(t)z$*. The pointwise worst-case distortion induced by u,*


$$\begin{array}{c} \phi_{i}^{u}(t,z)=\frac{\gamma a_{i}(u(t,z))-\ell_{i,t}F_{z}(t,z)}{\kappa_{i}},\;i=1,2,3,4, \end{array}$$ (5.23)

*is affine in z:*


$$\begin{array}{c} \phi^{u}(t,z)=p^{u}(t)+R^{u}(t)z, \end{array}$$ (5.24)

*with deterministic bounded* $p^{u}:[0,T]\to {\mathbb{R}}^{4}$ *and* $R^{u}:[0,T]\to {\mathbb{R}}^{4\times 1}$*. Moreover, there are finite constants* $C_{p}(L_{u})$ *and* $C_{R}(L_{u})$*, depending only on* $L_{u},\gamma ,\kappa_{i},\Sigma ,\sup_{t}\|\ell_{t}\|,\sup_{t}|A_{2}(t)|,\sup_{t}|B_{1}(t)|$*, such that*


$$\begin{array}{c} \sup_{t}\|p^{u}(t)\|\leq C_{p}(L_{u}),\;\sup_{t}\|R^{u}(t)\|\leq C_{R}(L_{u}). \end{array}$$ (5.25)

*The candidate* $u^{*}$ *in (5.13) belongs to* ${𝒰}_{aff}(L_{u}^{*})$ *for some finite* $L_{u}^{*}$, with the additional property $q^{*}\in [0,1]$*, and* $\phi^{*}=\phi^{u^{*}}$ *belongs to* $\Theta_{aff}^{int}$ *whenever Condition (I) in Lemma 5.7 holds.*

*Proof.* The derivation is provided in Appendix A, subsection “Affine closure of feedback controls and distortions.”

### 5.3 Constrained retention and investment constraints

**Proposition 5.6** (Retention with interval constraint). *If only the proportional reinsurance control is constrained by* q∈[0,1] *and the investment controls remain unconstrained, then the optimal retention is*


$$\begin{array}{c} q_{\left[0,1\right]}^{*}=\min\{1,\max\{0,q^{unc}\}\},\;q^{unc}=\frac{c\vartheta }{\gamma d_{3}b^{2}},\;d_{3}=1+\frac{1}{\kappa_{3}}. \end{array}$$ (5.26)

Proof. The derivation is provided in Appendix A, subsection “Retention under the interval constraint.”

If investment constraints or leverage constraints are also imposed, and the admissible set $C_{\pi }\subset {\mathbb{R}}^{2}$ is closed and convex, the stock feedback becomes the $M_{\pi }$-metric projection


$$\begin{array}{c} \pi_{C_{\pi }}^{*}(t,z)=\text{arg}\min_{\pi \in C_{\pi }}\left[\frac{1}{2}\gamma^{2}\pi^{⊤}M_{\pi }\pi -\gamma \{m_{\pi }(z)+F_{z}(t,z)\Lambda_{\pi ,t}{\}}^{⊤}\pi \right]. \end{array}$$ (5.27)

This projection is an additional portfolio constraint and is not used in the benchmark model. The interval-constrained retention rule (5.26) is already incorporated into the main HJBI, reduced PDE, coefficient system, and verification theorem.

### 5.4 Verification theorem

For the verification result, define the Hamiltonian


$$\begin{array}{c} H(t,x,z,u,\phi )=V_{t}+L^{u,\phi }V-\frac{1}{2}V\phi^{⊤}\Gamma \phi . \end{array}$$ (5.28)

For $u\in {𝒰}_{aff}(L_{u})$, let $\phi^{u}$ be the pointwise minimizer (5.23). The feedback $u^{*}$ is given by (5.13), and $\phi^{*}=\phi^{u^{*}}$.

**Lemma 5.7** (Exponential integrability of affine distortions). *Fix a bounded affine feedback* $u\in {𝒰}_{aff}(L_{u})$*, and let* ϕ(t,z)=p(t)+R(t)z *be affine with bounded continuous* $p:[0,T]\to {\mathbb{R}}^{4}$ *and* $R:[0,T]\to {\mathbb{R}}^{4\times 1}$*. Define*


$$\begin{array}{c} L_{p}=\sup_{0\leq s\leq T}\|p(s)\|,\;L_{R}=\sup_{0\leq s\leq T}\|R(s)\|,\;L_{\ell }=\sup_{0\leq s\leq T}\|\ell_{s}\|. \end{array}$$ (5.29)

*Let*


$$\begin{array}{c} v_{T}^{\mathbb{P}}=\frac{L_{\ell }^{2}}{2\alpha_{Z}}(1-e^{-2\alpha_{Z}T}). \end{array}$$ (5.30)

*Condition (I) consists of the following two inequalities:*


$$\begin{array}{cc} 2L_{R}^{2}Tv_{T}^{\mathbb{P}} & <1, \end{array}$$ (5.31)

$$\begin{array}{cc} 2{\bar{\theta }}_{\phi }(T+1)v_{T}^{\phi } & <1. \end{array}$$ (5.32)

*Here*


$$\begin{array}{c} L_{b}^{\phi }=\sup_{0\leq s\leq T}|-\alpha_{Z}-\ell_{s}^{⊤}R(s)|,\;v_{T}^{\phi }=\left\{ \begin{array}{cc} L_{\ell }^{2}(e^{2L_{b}^{\phi }T}-1)/(2L_{b}^{\phi }), & L_{b}^{\phi }>0,\\ L_{\ell }^{2}T, & L_{b}^{\phi }=0, \end{array}\right. \end{array}$$ (5.33)

*and* ${\bar{\theta }}_{\phi }<\infty$ *is constructed from the bounds of* $A_{2},B_{1},C,u_{0},U_{1},p,R,\Sigma ,m_{0},m_{1},\gamma ,\Gamma$*, as explained in the proof, so that for every stopping time* τ≤T*, the absolute stopped payoff, running penalty, and stochastic-integral quadratic variations in the verification proof are dominated by*


$$\begin{array}{c} K_{\phi }\exp\left\{{\bar{\theta }}_{\phi }\left(1+{\hat{Z}}_{\tau }^{2}+\int_{t}^{T}{\hat{Z}}_{s}^{2}\;ds\right)\right\} \end{array}$$ (5.34)

*for some finite* $K_{\phi }$*. Under Condition (I), the Girsanov density is a true* ℙ*-martingale, and the stopped local martingales in the verification proof are uniformly integrable under the corresponding* ${\mathbb{Q}}^{\phi }$.

*Proof.* The derivation is provided in Appendix A, subsection “Exponential integrability of affine distortions.”

**Theorem 5.8** (Conditional verification over a general admissible control-distortion class). *Let* $V\in C^{1,2,2}([0,T)\times {\mathbb{R}}^{2})\cap C([0,T]\times {\mathbb{R}}^{2})$ *satisfy the terminal condition V(T,x,z) = U(x), Assumption 3.4, and the HJBI* equation (4.2)*. Suppose there exists a feedback* $u^{*}\in A^{V}(t,x,z)$*, with* $q^{*}\in [0,1]$*, and a distortion* $\phi^{*}\in \Theta^{V}(t,x,z;u^{*})$*, such that*


$$\begin{array}{c} H(t,x,z,u^{*},\phi^{*})=0, \end{array}$$ (5.35)

*and the saddle inequalities hold pointwise:*


$$\begin{array}{cc} H(t,x,z,u,\phi^{u}) & \leq 0,\;u\in A^{V}(t,x,z), \end{array}$$ (5.36)

$$\begin{array}{cc} H(t,x,z,u^{*},\phi ) & \geq 0,\;\phi \in \Theta^{V}(t,x,z;u^{*}). \end{array}$$ (5.37)

*Here* $\phi^{u}$ *is the pointwise minimizer of the Hamiltonian induced by u, and* $u\in A^{V}(t,x,z)$ *means precisely that this minimizer is admissible in the sense of Assumption 3.4. Then*


$$\begin{array}{c} V(t,x,z)=\sup_{u\in A^{V}(t,x,z)}\inf_{\phi \in \Theta^{V}(t,x,z;u)}J(t,x,z;u,\phi )=\inf_{\phi \in \Theta^{V}(t,x,z;u^{*})}J(t,x,z;u^{*},\phi ), \end{array}$$ (5.38)

*and* $\left(u^{*},\phi^{*}\right)$ *is a saddle point over the explicit admissible control-distortion class. The result is conditional on smoothness, attainability of the pointwise saddle, and the stated integrability assumptions; it is not an unrestricted optimality theorem over arbitrary progressively measurable controls.*

*Proof.* The derivation is provided in Appendix A, subsection “Conditional verification theorem.”

**Proposition 5.9** (Checkable sufficient admissibility of the semi-closed-form candidate). *Consider the feedback* $u^{*}$ *in (5.13) and the induced distortion* $\phi^{*}$ *in (4.4). Suppose Condition (I) in Lemma 5.7 holds for the affine stock feedback and its induced distortion. Then* $u^{*}\in A^{V}(t,x,z)$ *and* $\phi^{*}\in \Theta^{V}(t,x,z;u^{*})$*.*

*Proof.* The derivation is provided in Appendix A, subsection “Checkable sufficient admissibility conditions.”

**Remark 5.10** (Affine class as a checkable sufficient subclass). The bounded affine class ${𝒰}_{aff}(L_{u})$ and the affine distortion class $\Theta_{aff}^{int}$ are not the main optimization domain. They are retained as a convenient way to check the exponential-integrability requirements in Theorem 5.8. Lemma 5.7 gives explicit sufficient conditions, such as the Novikov-side bound $2C_{R}(L_{u}{)}^{2}Tv_{T}^{\mathbb{P}}<1$, under which affine candidates and their induced distortions belong to the explicit admissible classes. Affinity is used to construct and verify the semi-closed-form feedback; it is not imposed as the definition of admissibility.

### 5.5 Special cases

**Corollary 5.11 (Model reductions).** *The joint-filtering model admits the following benchmark cases.*

*Proof.* The derivation is provided in Appendix A, subsection “Model reductions and limiting cases.”

### 5.6 Economic implications

Stock returns are not merely investment opportunities in this model; they are market-based signals about hidden green quality. Green stock returns, orthogonalized brown stock returns, and ESG ratings jointly determine the belief ${\hat{Z}}_{t}$. The ESG rating is therefore only one component of learning, and its noise level $\sigma_{Y}$ controls rating informativeness rather than total informativeness.

The stock strategy decomposes as


$$\begin{array}{c} \pi^{*}(t,z)=\frac{1}{\gamma }M_{\pi }^{-1}m_{\pi }(z)+\frac{1}{\gamma }M_{\pi }^{-1}\Lambda_{\pi ,t}F_{z}(t,z). \end{array}$$ (5.39)

The first component is the myopic demand from expected excess returns. The second component is the filtering hedging demand. It appears because the same stock-return shocks affect both current wealth and future beliefs about green quality. The constrained retention $q^{*}=\Pi_{\left[0,1\right]}(q^{unc})$ is determined separately by the scalar underwriting term.

The rating-innovation ambiguity parameter $\kappa_{4}$ does not directly enter the wealth risk matrix *M*, because the fourth row of Σ is zero. Its influence follows the indirect channel


$$\begin{array}{c} \kappa_{4}⟶D_{\kappa }⟶\nu_{t}⟶(A_{2},B_{1},C)⟶F_{z}⟶\pi^{*}. \end{array}$$ (5.40)

Thus rating-innovation ambiguity changes the value of information and the belief sensitivity of the value function, even though ESG rating noise does not directly add wealth volatility. By contrast, $\kappa_{1}$ and $\kappa_{2}$ have a double role: they enter $M_{\pi }$, changing myopic risky exposure, and they enter $\Lambda_{\pi ,t}$, changing the learning-related hedging demand.

The parameter $\sigma_{Y}$ controls only the ESG rating information channel. If $\sigma_{Y}\to \infty$, the rating signal becomes uninformative, but learning may persist through stock returns whenever $\beta_{g}$ or $\beta_{⟂}$ is nonzero. Conversely, if stock-return learning is absent, the model reduces to rating-only learning and the hedging demand disappears.

For reinsurance, the unconstrained retention is


$$\begin{array}{c} q^{unc}=\frac{c\vartheta }{\gamma d_{3}b^{2}},\;d_{3}=1+\frac{1}{\kappa_{3}}. \end{array}$$ (5.41)

Higher risk aversion γ and higher underwriting volatility *b* reduce retention. A smaller $\kappa_{3}$ means stronger underwriting ambiguity, increases *d*_3_, and lowers retention. A larger reinsurance loading ϑ raises the marginal drift term cϑq in the present specification, so it raises the unconstrained retention; this comparative static follows from the chosen surplus drift and should be interpreted within that modeling convention. If the projected rule $q_{\left[0,1\right]}^{*}$ is used, these comparative statics apply only in the interior region; at the boundaries *q* = 0 or *q* = 1, the response can become flat.

This joint observation mechanism is the key theoretical distinction from ordinary robust ESG investment models and from standard partial-information reinsurance-investment models. It captures the fact that market prices and ratings jointly transmit information about hidden green quality, while rating-innovation ambiguity affects the credibility assigned to the rating channel.

## 6 Numerical analysis

### 6.1 Numerical implementation and benchmark design

This section reports the numerical experiments used to examine the model mechanisms, the materiality frontier, and insurance-capital risk. The script solves the deterministic ODEs, runs Monte Carlo experiments with common random numbers, computes paired standard errors. An independently reproduces the weak-rating comparison, adds bootstrap and Wilson uncertainty intervals, evaluates two transparent operational benchmarks, and performs a global parameter-uncertainty analysis. The parameters in both implementations are illustrative scenario inputs rather than calibrated estimates.

The numerical procedure follows the separated problem. We solve the filter variance Riccati equation


$$\begin{array}{c} {\dot{P}}_{t}=\sigma_{Z}^{2}-2\alpha_{Z}P_{t}-\chi P_{t}^{2}, \end{array}$$ (6.1)

then solve the backward Riccati-type system for $A_{2}(t),B_{1}(t),C(t)$. The stock feedback and constrained retention are evaluated from


$$\begin{array}{cc} \pi^{*}(t,z) & =\frac{1}{\gamma }M_{\pi }^{-1}\{m_{\pi }(z)+F_{z}(t,z)\Lambda_{\pi ,t}\},\\ F_{z}(t,z) & =2A_{2}(t)z+B_{1}(t),\\ q^{*} & =\Pi_{\left[0,1\right]}\left(\frac{c\vartheta }{\gamma d_{3}b^{2}}\right), \end{array}$$ (6.2)

and the stock rule is decomposed into myopic demand and filtering-hedging demand. All strategy comparisons use the same Brownian increments, so differences in CE, capital-warning probability, and other risk metrics are evaluated with paired Monte Carlo errors.

Reference CE is computed from terminal wealth under the reference model. The drift-distorted ${CE}_{wc}^{dd}$ is computed by simulating the wealth equation under the pointwise distorted drift induced by $\phi^{*}$, using the same Brownian paths. For insurance-capital risk, VaR_5_ denotes the 5% quantile of terminal wealth, and ES_5_ denotes the mean terminal wealth conditional on being below that quantile. Thus lower VaR_5_ or ES_5_ means worse downside performance; these are wealth-based rather than loss-based definitions. We also report $P(X_{T}<X_{0})$, the running capital-warning probability $P(\min_{t}X_{t}<L)$, and the solvency-adjusted CE $SACE=CE-\lambda_{warn}P(\min_{t}X_{t}<L)$ as a supplementary risk-adjusted statistic. Table 1 reports the benchmark inputs.

**Table 1:** Benchmark parameters for the primary numerical experiments.

Symbol	Description	Value	Symbol	Description	Value
*T*	Horizon	1.000	$\sigma_{g}$	Green volatility	0.220
*N*	Trading steps	252.000	$\sigma_{b}$	Brown volatility	0.250
$n_{path}$	Monte Carlo paths	5.000e + 04	ρ	Correlation	0.350
*x*_0_	Initial wealth	10.000	$\sigma_{Y}$	Rating noise	0.450
*z*_0_	Initial quality	0.200	$\delta_{0}$	Reference rating bias	0.080
${\hat{z}}_{0}$	Initial filter	0.200	*c*	Premium scale	1.000
*P*_0_	Initial variance	0.250	η	Insurer loading	0.200
*r*	Risk-free rate	0.020	ϑ	Reinsurer loading	0.350
$\alpha_{Z}$	Mean reversion	1.200	*b*	Underwriting volatility	0.500
$\bar{Z}$	Long-run quality	0.300	γ	Risk aversion	0.800
$\sigma_{Z}$	Quality volatility	0.250	$\kappa_{1}$	Financial ambiguity 1	4.000
$\mu_{g}^{0}$	Green intercept	0.055	$\kappa_{2}$	Financial ambiguity 2	4.000
$\mu_{b}^{0}$	Brown intercept	0.065	$\kappa_{3}$	Underwriting ambiguity	3.000
$\beta_{g}$	Green loading	0.035	$\kappa_{4}$	Rating-innovation ambiguity	2.500
$\beta_{b}$	Brown loading	0.030	*L*	Warning threshold	9.500

Values are illustrative rather than calibrated estimates.

The input values are chosen to place the model in an economically interpretable, numerically stable one-year insurance-investment scenario. The horizon is one year and the numerical time grid has 252 steps, corresponding to a daily discretization; the primary comparisons use 50,000 paths. Asset volatilities and risk premia are set to moderate annualized values, while the green-return loading $\beta_{g}$, orthogonal loading $\beta_{⟂}$, rating noise $\sigma_{Y}$, prior variance *P*_0_, mean reversion $\alpha_{Z}$, and latent volatility $\sigma_{Z}$ are selected so that both market prices and the ESG rating contain nondegenerate information about $Z_{t}$. The insurance parameters place the retention rule at or near an economically relevant constrained region, and a separate interior-retention scenario isolates its comparative statics. Ambiguity penalties are chosen to span weak, moderate, and stress cases while the ESS screen identifies numerically unreliable likelihood reweighting. These choices are motivated by the mechanisms to be displayed, not by a fit to an insurer or ESG-rating dataset. Consequently, no real-world dataset is analyzed and no empirical “data section” is required; all reported observations are simulated under the stated model.

The independent replication uses 12,000 paths and 300 nonparametric bootstrap resamples for CE, VaR_5_, and ES_5_, together with Wilson intervals for the capital-warning probability. Its global check uses a reproducible 40-point Latin hypercube and 2,500 paired paths per design. It varies $\sigma_{Y},\alpha_{Z},P_{0},\sigma_{Z},\beta_{g},\beta_{⟂},\kappa_{4}$, and γ over the documented illustrative ranges. These across-design ranges describe sensitivity within that hyper-rectangle; they are not sampling intervals for a calibrated population.

### 6.2 Benchmark validation and filtering-hedging demand

Fig 1 checks the deterministic ODE layer. The filter variance is solved forward, while the value-function coefficients are solved backward and interpolated to the Monte Carlo grid. Smooth paths for $P_{t},A_{2}(t),B_{1}(t)$, and *C*(*t*) provide a numerical stability check for the Kalman–Bucy and exponen*t*ial-quadratic Riccati systems.

**Fig 1:**
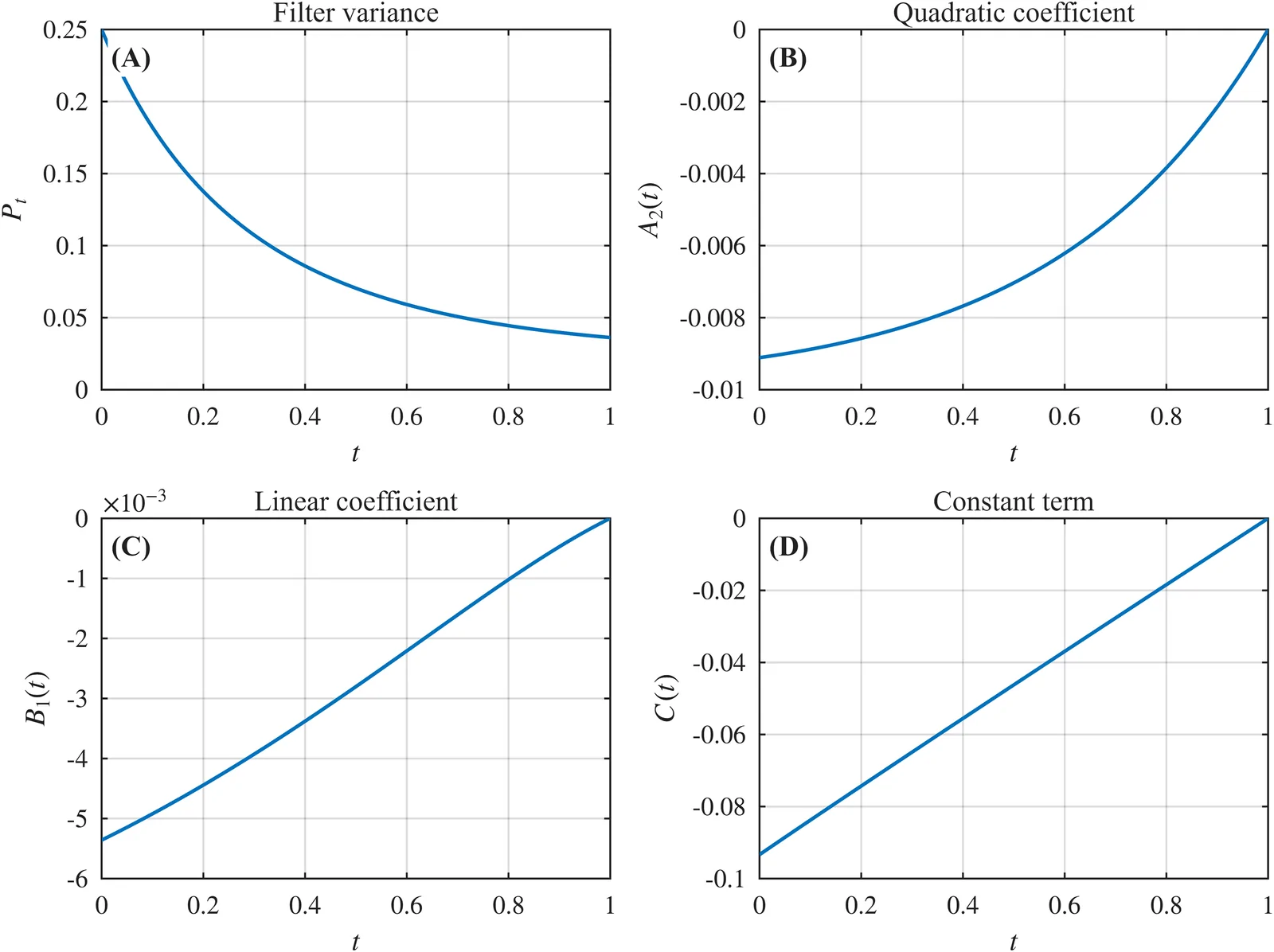
Filtering variance and Riccati coefficients under the benchmark calibration. The figure verifies the deterministic ODE layer used before Monte Carlo simulation.

Fig 2 isolates the mixed-derivative mechanism. The filtering-hedging demand is not introduced as a separate preference; it is generated by the fact that stock-return innovations affect both wealth and beliefs. Its magnitude is benchmark-dependent, but its presence confirms the channel represented by the $V_{xz}$ term in the separated HJBI equation.

**Fig 2:**
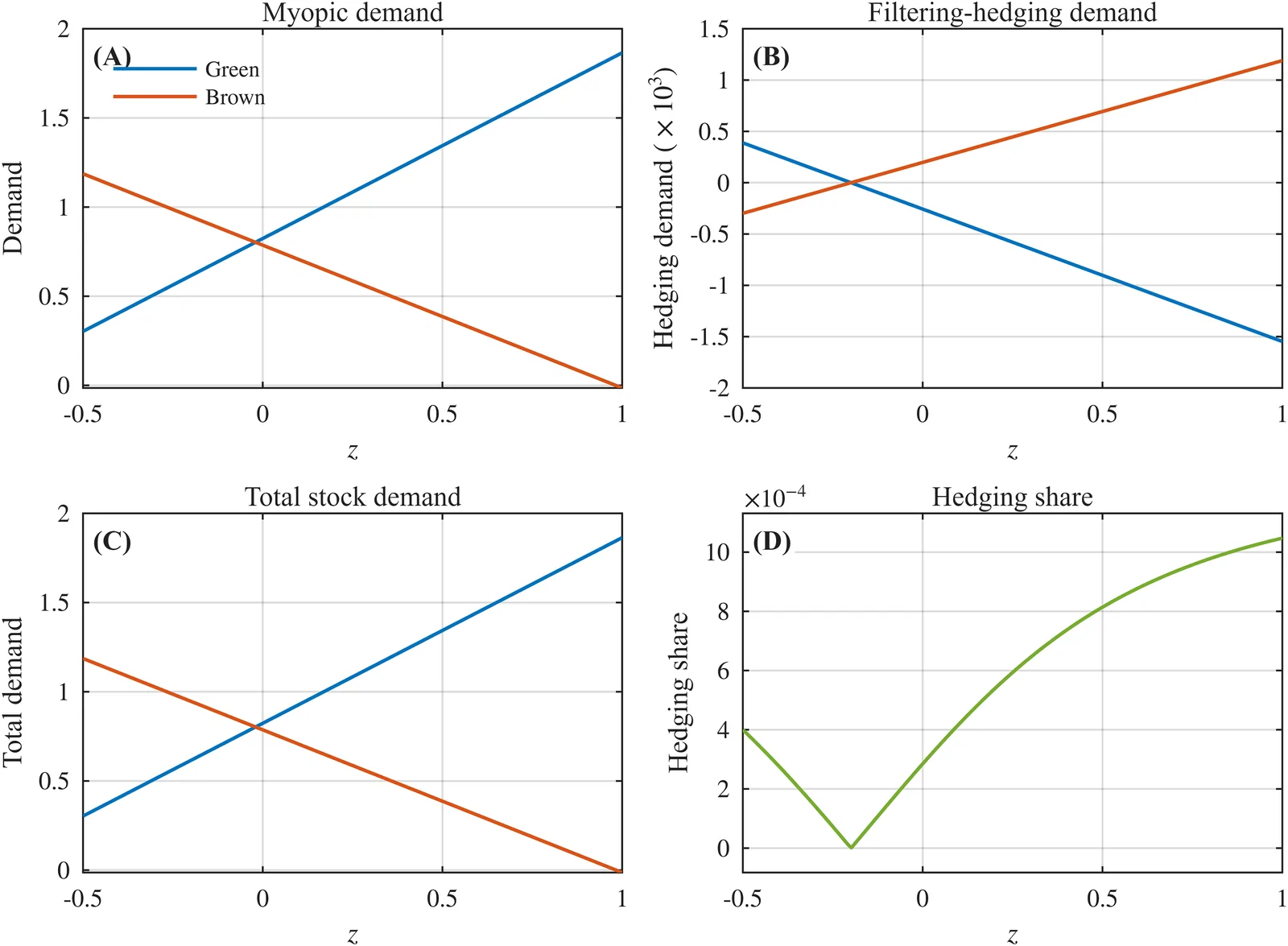
Myopic demand, filtering-hedging demand, total stock demand, and the hedging share at the benchmark midpoint. The hedging component comes from shared stock-return innovations and the mixed derivative $V_{xz}$.

### 6.3 Value of joint learning

The first experiment compares full information, joint learning, rating-only learning, and stock-return-only learning. Table 2 reports CE, drift-distorted CE, capital-warning probability, VaR_5_, ES_5_, terminal-loss probability, and filter MSE. The weak-rating scenario uses a higher rating-noise level and stronger market learning loadings to test whether joint learning improves belief accuracy rather than merely adding a redundant signal. Fig 3 displays the same comparison graphically, with the filter-MSE panel kept on a zero-based scale and the CE, VaR_5_, and ES_5_ panels zoomed only to make the ordering visible. The corresponding original-scale plots are supplied as S2 Fig in S1 File.

**Table 2:** Learning comparison summary.

Strategy	CE	${CE}_{wc}^{dd}$	Warn.	VaR_5_	ES_5_	Loss prob.	Filter MSE
Full	10.264	10.143	0.257	9.307	9.005	0.260	0
Joint	10.202	10.101	0.245	9.304	9.041	0.288	0.106
Rating	10.202	10.101	0.247	9.305	9.031	0.284	0.111
Stock	10.201	10.101	0.245	9.305	9.042	0.288	0.107

VaR_5_ and ES_5_ are wealth-based downside measures.

**Fig 3:**
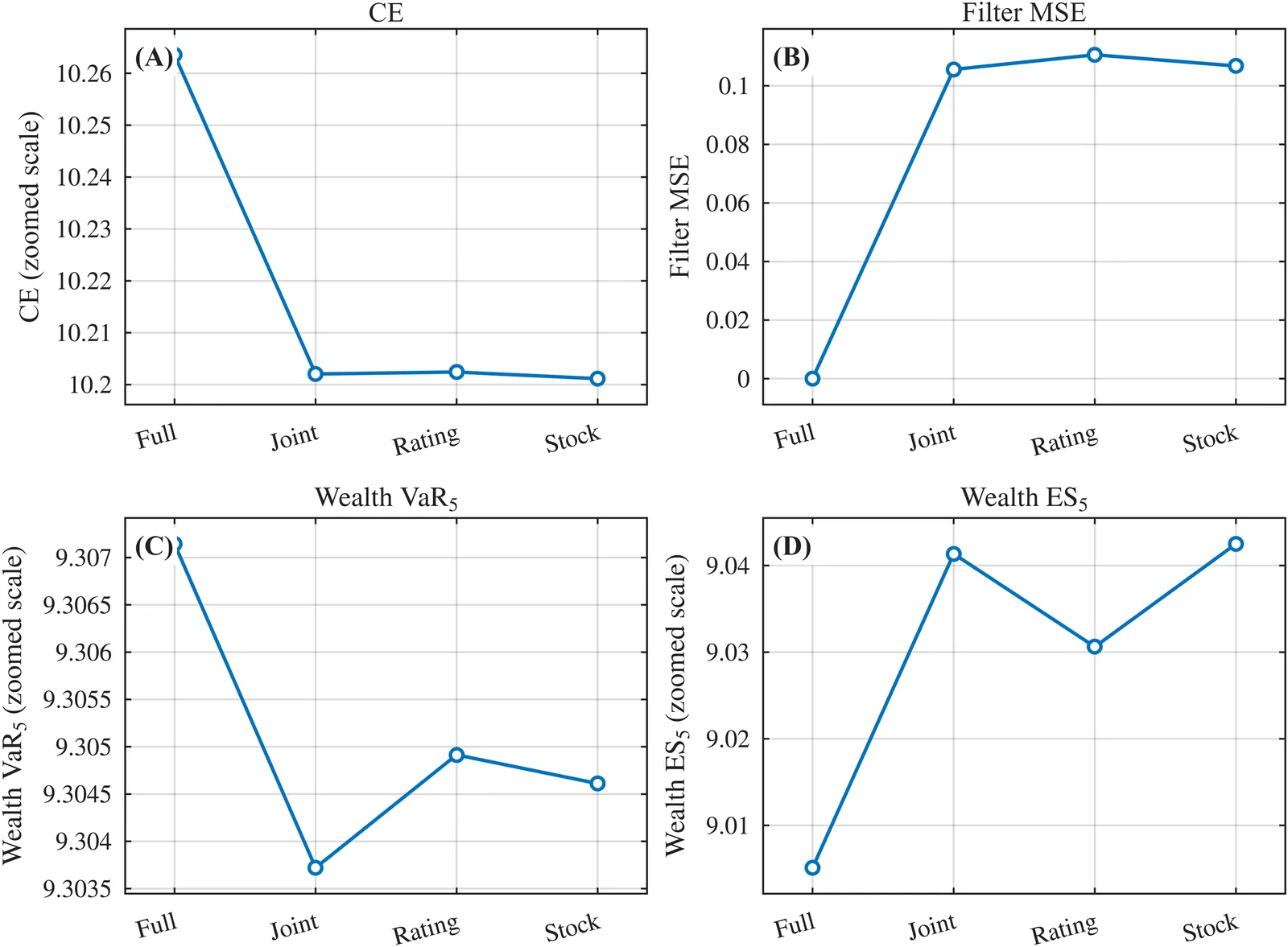
Learning-structure comparison. CE, VaR_5_, and ES_5_ use zoomed axes to show cross-strategy differences, while filter MSE is displayed on a zero-based scale. Because the absolute economic differences in CE, VaR_5_, and ES_5_ are small, the zoomed axes are used only to display the ordering across strategies; Table 2 should be used for magnitude interpretation.

These results should be read cautiously. Joint learning can improve belief accuracy when stock returns contain useful information about the hidden green quality, especially when the rating signal is weak. A lower filter MSE need not translate into a large benchmark CE gain, because the welfare effect also depends on risk aversion, the insurance surplus, and the capital threshold. In the independent replication (S1 Table in S1 File), the 95% intervals for the three partial-information strategies overlap substantially: joint-learning CE is 10.204 [10.192, 10.217], versus 10.205 [10.192, 10.217] for rating-only learning and 10.203 [10.192, 10.215] for stock-only learning. The experiment therefore supports the information-value mechanism without establishing statistical or universal welfare dominance.

### 6.4 Rating-innovation ambiguity channel decomposition

Rating-innovation ambiguity does not directly add a diffusion term to wealth. It affects policy through rating-innovation distortion, the value sensitivity $F_{z}$, and the filtering-hedging component of stock demand. Table 3 therefore reports an ESS-safe diagnostic decomposition: a joint robust/full-channel design, a frozen-$F_{z}$ design using the weak-ambiguity value sensitivity, a no-hedging policy that keeps only myopic demand, and a rating-only robust learning design. The no-hedging row is not an alternative optimum; it is a diagnostic policy used to isolate the mixed-derivative channel. Fig 4 plots these four designs so that the rating-distortion, value-sensitivity, hedging-share, and capital-warning channels can be compared panel by panel.

**Table 3:** Channel decomposition of rating-innovation ambiguity through rating distortion, value sensitivity, and filtering hedging.

Design	CE	Warn.	ES_5_	Avg. $\left|\phi_{4}^{*}\right|$	Avg. $F_{z}$	Hedge share	Δ Warn. (SE)	ESS
Joint/full	11.420	0.151	9.113	0.239	−0.274	0.216	0.004 (5.213e-04)	0.627
Frozen $F_{z}$	11.410	0.147	9.116	0.263	−0.301	0.241	0 (0)	0.576
No hedge	11.461	0.190	9.042	0.239	−0.274	0.216	0.043 (0.001)	0.588
Rating	11.571	0.187	9.174	0.336	−0.351	0	0.040 (0.002)	0.402

**Fig 4:**
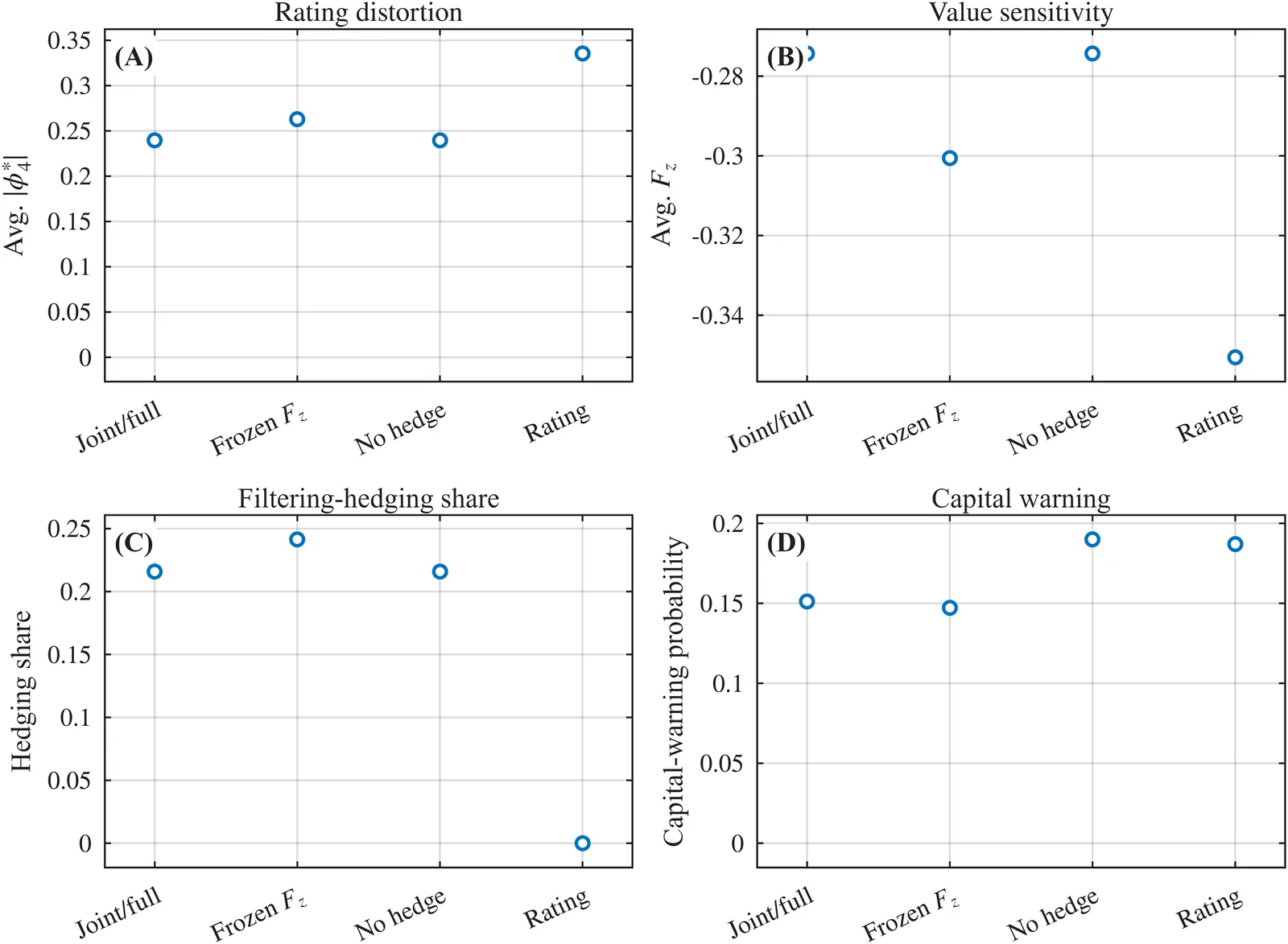
Rating-innovation ambiguity channel decomposition across diagnostic designs. The panels report rating distortion, value sensitivity, filtering-hedging share, and capital-warning probability for four categorical designs. Markers are not connected because the designs are diagnostic categories rather than points on a continuous parameter path.

This decomposition is the mechanism check behind the materiality analysis below. Differences across the rows indicate that rating-innovation ambiguity is transmitted through the value-sensitivity and filtering-hedging terms identified by the theory. The table reports ESS and clipping diagnostics so that the comparison is not based on degenerate likelihood weights.

### 6.5 ESS-screened rating-innovation ambiguity materiality frontier

The next experiment asks where rating-innovation ambiguity is numerically visible without relying on degenerate Girsanov weights. We vary $\kappa_{4}$ jointly with rating noise $\sigma_{Y}$ and, separately, with hidden-quality persistence $\alpha_{Z}$. Smaller $\kappa_{4}$ corresponds to stronger ambiguity about the rating innovation. Each grid point is compared with the same-scenario weak-ambiguity endpoint $\kappa_{4}=\infty$ using common random numbers. Cells with ESS ratio below 0.05 are treated as severe stress and are masked in the main figures; cells with ESS ratio between 0.05 and 0.10 are interpreted as moderate stress. Figs 5 and 6 report the two screened frontiers.

**Fig 5:**
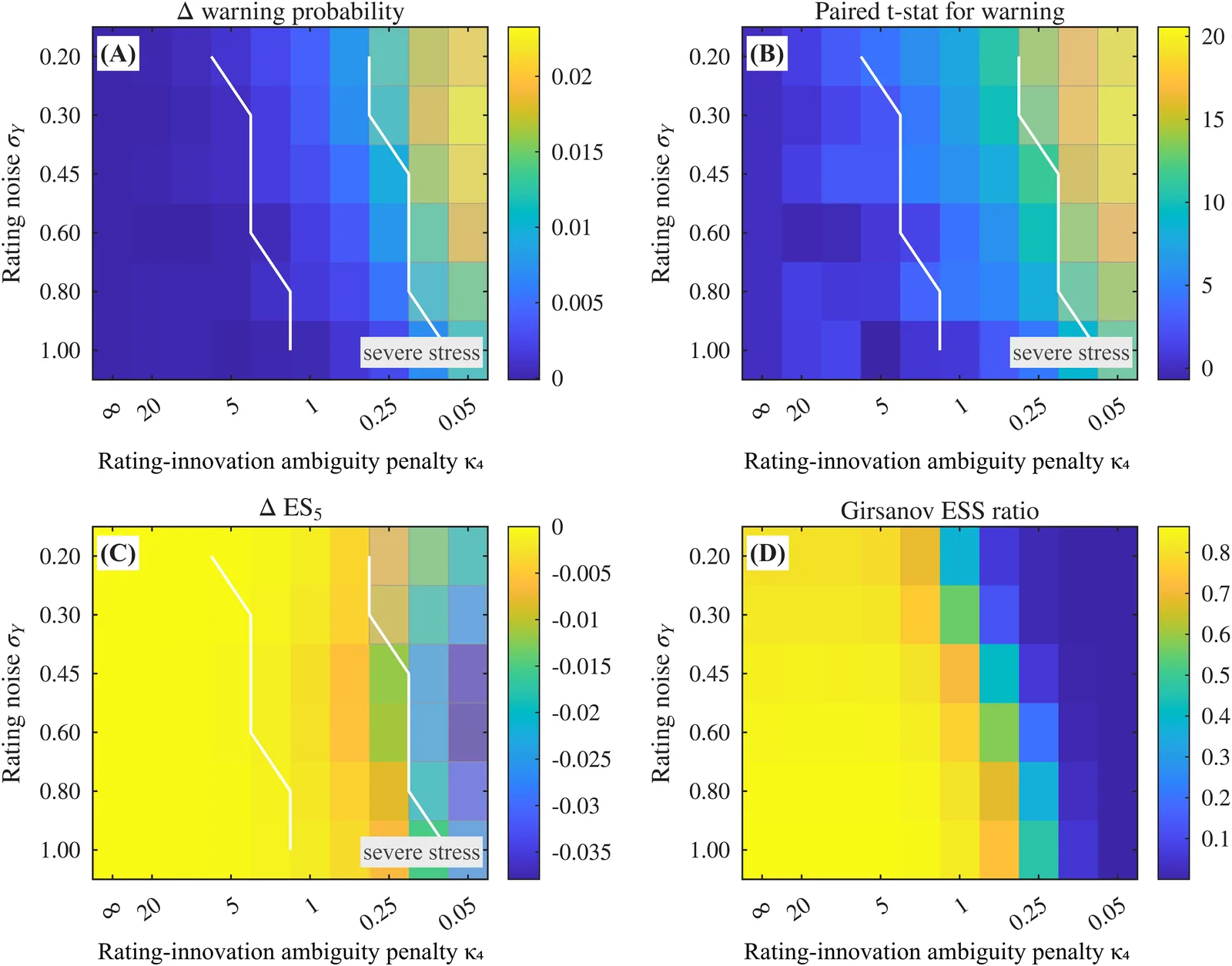
ESS-screened materiality frontier over rating-innovation ambiguity and ESG rating noise. Semi-transparent gray cells mark severe-stress cases with ESS ratio below 0.05; white contours mark ESS-screened materiality cells.

**Fig 6:**
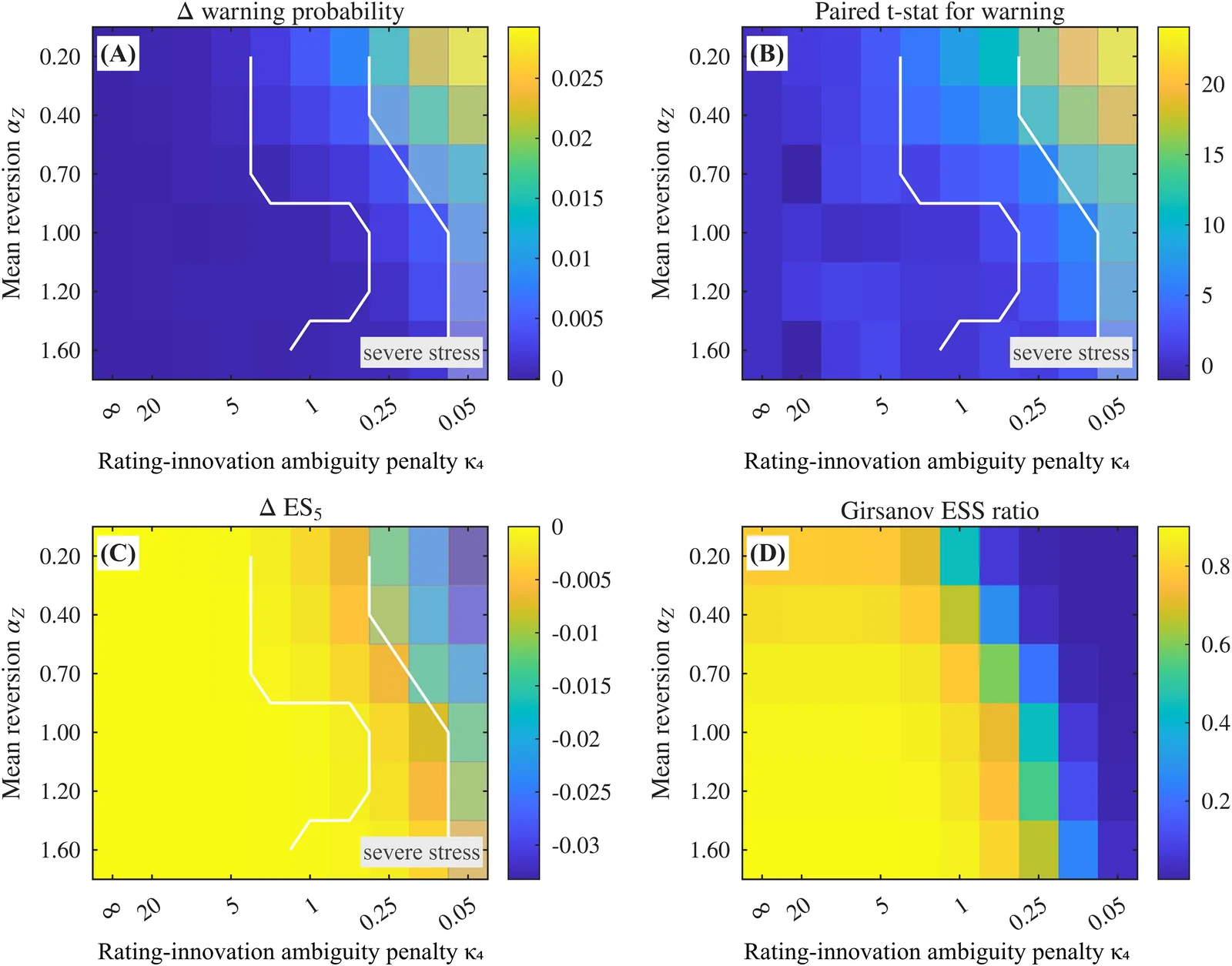
ESS-screened materiality frontier over rating-innovation ambiguity and hidden-quality persistence. Persistent hidden quality makes long-run learning and filtering-hedging demand more important, but low-ESS severe-stress cells are not used as main evidence.

We use an operational, simulation-specific definition of materiality. Let all changes compare a finite $\kappa_{4}$ cell with the matched $\kappa_{4}=\infty$ endpoint. A cell is classified as material only if ESS/n≥0.05 and at least one of the following conditions holds:


$$\begin{array}{cc} & \Delta p_{warn}>0.001\text{ and }|t_{warn}|\geq 2,\\ & \Delta {VaR}_{5}<-0.002,\;\Delta {ES}_{5}<-0.002,\\ & \Delta {CE}_{wc}^{dd}<-0.001\text{ and }t_{CE}\leq -2. \end{array}$$ (6.3)

The thresholds are transparent display rules for this illustrative wealth scale, not empirical welfare cutoffs. The warning-probability and CE rules additionally use paired-path *t*-screens because paired Monte Carlo errors are available for those grid outputs. The VaR_5_ and ES_5_ rules instead use economic-magnitude cutoffs; they are not presented as hypothesis tests, and bootstrap uncertainty for the principal quantile and tail comparisons is reported separately in S1 and S2 Tables in S1 File. This deliberate asymmetry should not be interpreted as evidence that every frontier tail-risk change is statistically distinguishable from zero. Alternative definitions can emphasize warning probability alone, tail risk alone, CE alone, relative changes, or a stricter ESS threshold. We therefore report the component metrics and the unmasked severe-stress cells separately, allowing readers to apply different criteria. Table 4 is the manuscript’s Table 4 and lists the ESS-screened cells under Eq (6.3). The conclusion is not that rating-innovation ambiguity produces large effects. It is more visible in selected downside metrics than in benchmark CE, and even those effects remain conditional on the parameter design.

**Table 4:** ESS-screened materiality-frontier summary.

Frontier	Row value	$\kappa_{4}$	Δ Warn.	Warn. *t*	Δ ES_5_	ESS	Class
sigmaY	0.200	5.000	0.001	4.218	−1.502e-04	0.789	reliable
sigmaY	0.200	2.000	0.003	5.926	−5.529e-04	0.690	reliable
sigmaY	0.200	1.000	0.004	7.576	−0.001	0.376	reliable
sigmaY	0.200	0.500	0.008	10.756	−0.004	0.063	moderate stress
sigmaY	0.300	2.000	0.002	4.507	−7.552e-04	0.757	reliable
sigmaY	0.300	1.000	0.004	6.966	−0.002	0.556	reliable
sigmaY	0.300	0.500	0.007	9.858	−0.004	0.126	reliable
sigmaY	0.450	2.000	0.002	4.991	−0.001	0.807	reliable
sigmaY	0.450	1.000	0.003	6.070	−0.003	0.710	reliable
sigmaY	0.450	0.500	0.005	7.989	−0.006	0.409	reliable
sigmaY	0.450	0.250	0.009	11.437	−0.012	0.058	moderate stress
sigmaY	0.600	2.000	4.667e-04	1.510	−0.001	0.831	reliable
sigmaY	0.600	1.000	0.002	4.270	−0.003	0.769	reliable
sigmaY	0.600	0.500	0.004	6.050	−0.005	0.579	reliable
sigmaY	0.600	0.250	0.008	9.893	−0.011	0.198	reliable
sigmaY	0.800	1.000	0.002	4.528	−0.002	0.808	reliable
sigmaY	0.800	0.500	0.003	5.214	−0.004	0.678	reliable
sigmaY	0.800	0.250	0.005	8.109	−0.008	0.362	reliable

Severe-stress cells are excluded from the main evidence.

### 6.6 Reinsurance interior-retention and insurance-capital risk

The benchmark can put the projected retention near the boundary, which makes the reinsurance channel visually less informative. We therefore add an interior-retention scenario with *b* = 0.75, γ=0.80, and $\kappa_{3}=3$. Since


$$\begin{array}{c} q^{unc}=\frac{c\vartheta }{\gamma (1+1/\kappa_{3})b^{2}}, \end{array}$$ (6.4)

the benchmark values *c* = 1 and ϑ=0.35 imply $q^{unc}\approx 0.583$, so the projected rule enters the interior of [0,1]. Table 5 varies $\kappa_{3}$ and reports $q^{*}$, CE, ${CE}_{wc}^{dd}$, warning probability, VaR_5_, and ES_5_. Fig 7 then shows how the interior retention rule is transmitted to the capital-warning, VaR_5_, and ES_5_ panels.

**Table 5:** Interior reinsurance-retention scenario with benchmark underwriting and ESG-information stress metrics.

$\kappa_{3}$	$q^{unc}$	$q^{*}$	CE	${CE}_{wc}^{dd}$	Warn.	VaR_5_	ES_5_
0.500	0.259	0.259	9.986	9.905	0.148	9.421	9.268
1.000	0.389	0.389	10.012	9.923	0.182	9.375	9.193
2.000	0.519	0.519	10.031	9.949	0.229	9.304	9.094
3.000	0.583	0.583	10.037	9.965	0.253	9.262	9.038
5.000	0.648	0.648	10.042	9.983	0.277	9.219	8.979
10.000	0.707	0.707	10.044	10.001	0.299	9.180	8.924
∞	0.778	0.778	10.046	10.025	0.324	9.130	8.855

**Fig 7:**
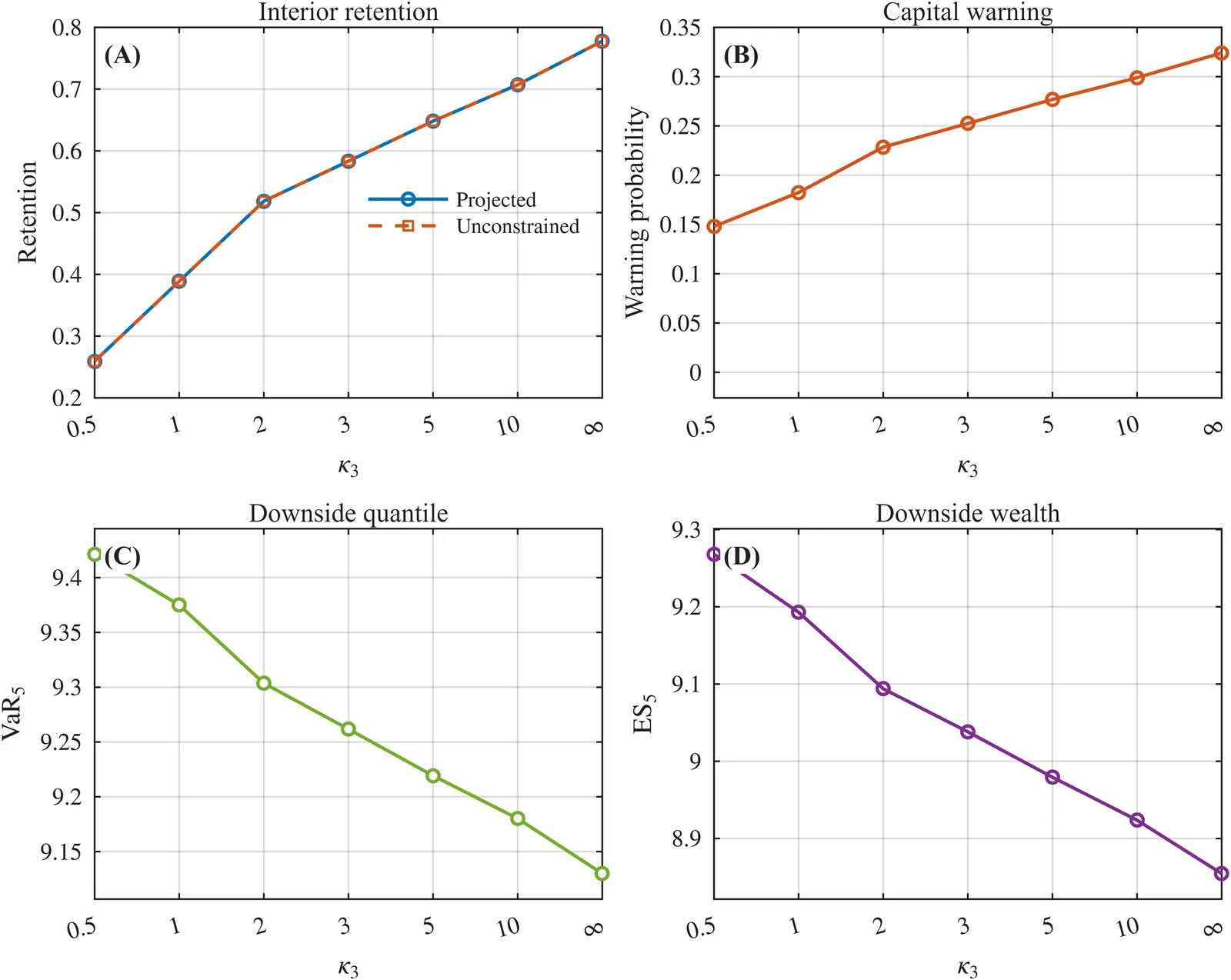
Interior-retention channel under underwriting ambiguity. Panel (A) reports the unconstrained and projected retention levels as functions of $\kappa_{3}$. Panels (B)–(D) report the model-implied capital-warning probability, wealth VaR_5_, and wealth ES_5_ under the same interior-retention scenario. All results are based on illustrative parameters and should be interpreted as mechanism evidence rather than calibrated empirical estimates.

The purpose of this interior-retention scenario is to isolate the underwriting-ambiguity channel in a region where the retention constraint is not binding. Underwriting ambiguity changes the retention rule through *d*_3_, and the resulting changes in retained underwriting exposure are reflected in the model-implied warning probability, VaR_5_, and ES_5_. These results should be interpreted as mechanism evidence under illustrative parameters rather than as calibrated empirical estimates.

A two-dimensional diagnostic surface over $\left(\kappa_{3},\kappa_{4}\right)$ is reported in S1 Fig in S1 File. In the present calibration, this surface indicates that the interior-retention capital-warning profile is mainly driven by underwriting ambiguity $\kappa_{3}$, while the marginal contribution of rating-innovation ambiguity $\kappa_{4}$ is comparatively weaker in this particular stress design.

### 6.7 Robust, nonrobust, and naive strategies under systematic rating-bias misspecification

The final experiment adds an out-of-model drift to the ESG rating signal:


$$\begin{array}{c} \;dY_{t}=(Z_{t}+\delta_{0}+g_{true})\;dt+\sigma_{Y}\;dW_{t}^{4}, \end{array}$$ (6.5)

while the strategies are computed under the reference model. Here $g_{true}$ is a fixed, out-of-model systematic bias in the data-generating rating drift. It is conceptually distinct from $\phi_{4}$, which is an endogenous worst-case distortion of the rating innovation inside the robust control problem, and from $\sigma_{Y}$, which is observation noise. The comparison includes robust joint learning, nonrobust joint learning with $\kappa_{i}=\infty$, rating-only robust learning, a naive ESG-rating strategy based on rolling rating increments, and a myopic-only robust diagnostic policy. Table 6 reports the selected scenarios, and Fig 8 plots the strategy profiles across the bias grid. Original-scale panels are supplied as S3 Fig in S1 File.

**Table 6:** Summary of robust, nonrobust, and naive strategies under selected systematic rating-bias scenarios.

$g_{true}$	Strategy	CE	Warn.	ES_5_	${\bar{\pi }}_{g}$
0	Robust	10.160	0.136	9.073	1.074
0	Nonrobust	10.163	0.161	9.000	1.342
0	Naive	10.219	0.192	8.911	1.276
0.100	Robust	10.160	0.136	9.073	1.092
0.100	Nonrobust	10.163	0.162	8.999	1.365
0.100	Naive	10.220	0.192	8.908	1.369
0.300	Robust	10.161	0.136	9.071	1.127
0.300	Nonrobust	10.164	0.163	8.996	1.409
0.300	Naive	10.223	0.192	8.902	1.554

**Fig 8:**
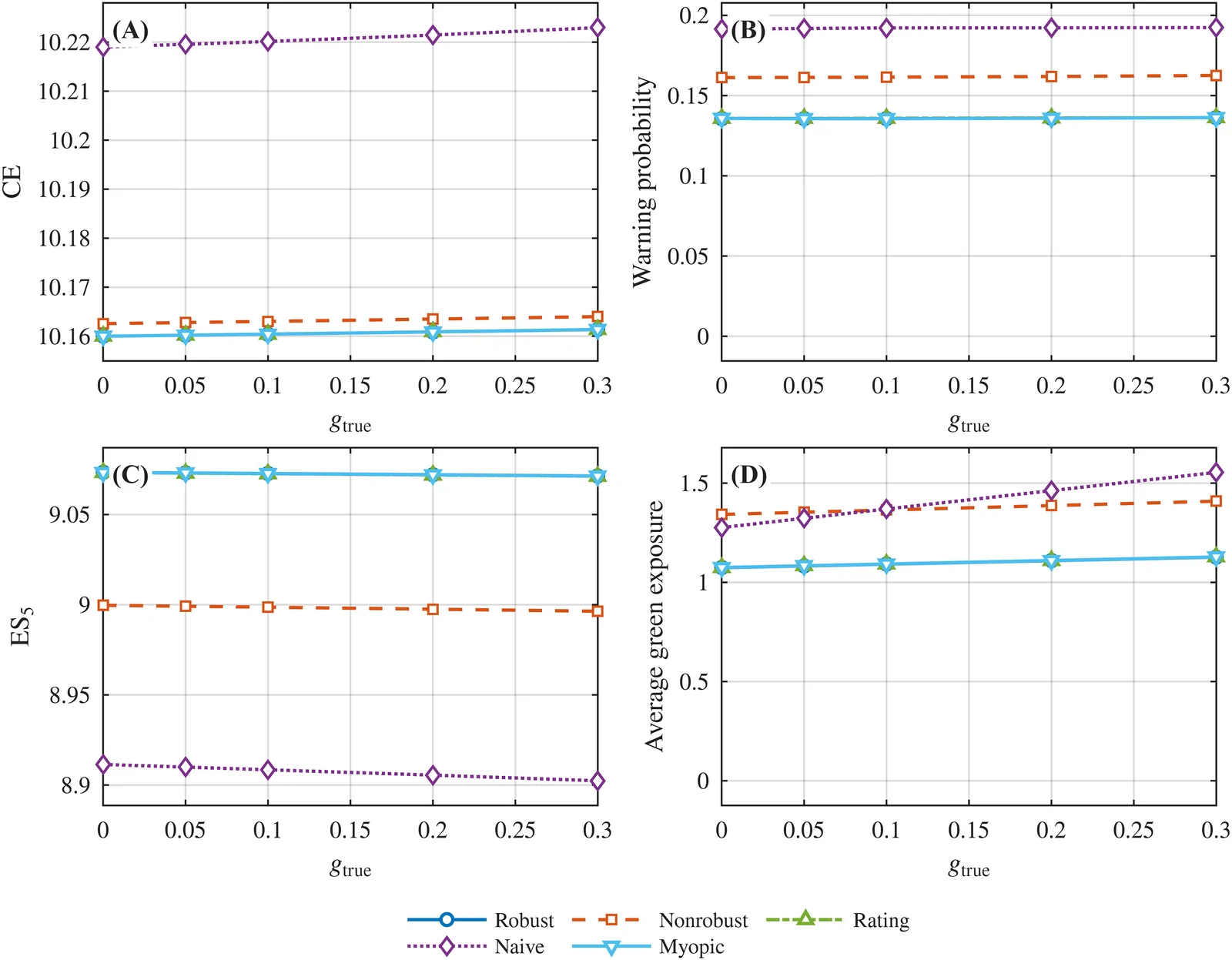
Strategy performance under systematic rating-bias misspecification. CE and ES_5_ use zoomed axes to show cross-strategy differences as $g_{true}$ increases. The fixed bias $g_{true}$ is not the endogenous robust-control distortion $\phi_{4}$.

The complete misspecification table is reported in the supporting files.

The robust strategy need not maximize reference CE in every scenario. Its intended role in this stress design is to moderate sensitivity to an unmodeled systematic rating bias and downside capital exposure. The naive rule can look attractive under reference CE when it takes aggressive positions, but the relevant insurance comparison includes warning probability, VaR_5_, ES_5_, dispersion, and exposure sensitivity. This experiment is descriptive stress testing and does not identify an empirical greenwashing process.

### 6.8 Independent uncertainty and operational-benchmark checks

The independent numerical workflow provides three checks requested for the revision. First, S1 Table in S1 File reports uncertainty intervals for the weak-rating comparison. The substantial overlap among partial-information strategies confirms that their economic differences are small on the benchmark wealth scale. Second, S2 Table in S1 File compares the dynamic robust rule with a policy that holds its initial robust allocation fixed and with an insurance-only policy. Dynamic robust and static CE are almost identical (10.161 and 10.161), whereas insurance-only has lower CE (10.097) but better VaR_5_ and ES_5_. Thus no benchmark uniformly dominates across reward and downside metrics, and the dynamic rule’s benefit is not a general empirical performance claim. Uncertainty intervals are reported for the principal cross-strategy comparisons in S1 and S2 Tables in S1 File; main-text Tables 3–7 are mechanism, frontier, sensitivity, or numerical-diagnostic summaries rather than a complete set of inferential tables.

**Table 7:** Girsanov effective-sample-size and clipping diagnostics for the main numerical evidence.

Experiment	Mean $E_{T}$	SD $E_{T}$	Q01	Q99	Min ESS	Max clip	Severe cells	Class
Benchmark	1.001	0.173	0.659	1.472	0.971	0	0	reliable
Channel	0.997	1.214	0.013	3.877	0.402	0	0	reliable
Frontier A main cells	0.999	4.264	0	8.528	0.052	0	13	moderate stress
Frontier B main cells	0.998	3.964	0	8.483	0.060	0	11	moderate stress
Interior	1.001	0.325	0.453	1.982	0.905	0	0	reliable
Misspecification	1.000	0.176	0.652	1.475	0.970	0	0	reliable

Third, S3 Table and S4 Fig in S1 File summarize the 40-point Latin-hypercube analysis. Relative to the paired $\kappa_{4}=\infty$ endpoint, the median CE and warning-probability changes are essentially zero; the 2.5–97.5% ranges are approximately $\left[-4.8\times 10^{-7},1.26\times 10^{-4}\right]$ for CE and $\left[-4.0\times 10^{-4},8.1\times 10^{-4}\right]$ for warning probability. ES_5_ changes are adverse in 97.5% of designs, but their absolute scale remains small (range about $\left[-7.31\times 10^{-4},-1.9\times 10^{-7}\right]$). The direction of the tail-risk mechanism is therefore more stable than its economic magnitude. These ranges describe the chosen scenario box and are neither confidence intervals for real insurers nor evidence of external calibration.

### 6.9 Numerical summary

Table 7 reports Girsanov effective-sample-size and clipping diagnostics. The effective sample size is


$$\begin{array}{c} ESS=\frac{(\sum_{i}E_{T}^{i}{)}^{2}}{\sum_{i}(E_{T}^{i}{)}^{2}}, \end{array}$$ (6.6)

reported as a ratio to the number of paths. The main channel, interior-retention, and misspecification comparisons use ESS-safe designs. In the frontier figures, cells with ESS ratio below 0.05 are masked as severe stress and excluded from the main materiality summary; cells between 0.05 and 0.10 are treated as moderate stress. Clipping frequencies are reported because any material clipping would indicate that numerical truncation, rather than the control model, may be driving part of the result.

The numerical conclusions are limited in scope. Benchmark CE effects remain small, and rating-innovation ambiguity is not presented as uniformly large. The main contribution of the numerical section is to identify a mechanism, an ESS-screened materiality region, and a model-implied downside-risk channel: rating-innovation distortion affects value sensitivity, filtering-hedging demand, and insurance-capital metrics more visibly than benchmark CE alone. The independent results reinforce the caution: partial-information intervals overlap, dynamic and static policies are similar in the tested benchmark, and global sensitivity shows a more consistent direction for ES_5_ than for CE or warning probability, but with small absolute changes. The robust strategy is not presented as uniformly CE-maximizing under the reference model; under the tested stress designs, it is consistent with lower systematic-rating-bias sensitivity and more moderate downside exposure. Finally, the interior-retention scenario illustrates how underwriting ambiguity can shape the model-implied downside capital-risk profile through the retention channel.

## 7 Conclusion

This paper studies a robust reinsurance-investment problem in which an insurer faces both underwriting risk and uncertainty about ESG information. The modeling premise is that the green quality relevant for investment is not directly observed. Instead, the insurer learns from green stock returns, brown stock returns, and an ESG rating signal. The rating signal is informative but noisy, and its innovation is subject to model ambiguity. The insurer therefore chooses proportional reinsurance and green/brown stock positions while guarding against worst-case distortions of the relevant innovations.

The analysis separates three objects that were previously conflated in the terminology: rating noise $\sigma_{Y}$, endogenous rating-innovation ambiguity $\phi_{4}$, and the fixed systematic rating-bias stress $g_{true}$. After orthogonalizing the observation system, the hidden green quality admits a Kalman–Bucy filter driven by stock-return and rating innovations. This filtering equation turns the partial-information problem into a completely observed robust control problem in the filtered state. A key feature is that stock-return innovations appear in both the wealth dynamics and the belief dynamics. As a result, the separated HJBI equation contains a mixed derivative term. This term produces a filtering hedging demand, which is distinct from the myopic investment demand generated by the current filtered estimate of green quality.

Under CARA utility and a value-dependent ambiguity penalty, the transformed HJBI equation admits an exponential-quadratic value function. The worst-case distortion is obtained pointwise from the Hamiltonian, the stock feedback is affine in the filtered state, and the proportional retention is projected onto q∈[0,1]. The coefficients of the value function solve a Riccati-type ODE system. The verification theorem is conditional on explicit admissibility and integrability assumptions, as is standard in exponential-utility robust stochastic control with unbounded state variables; the affine Markov class is used as a checkable sufficient subclass for the semi-closed-form candidate.

The numerical experiments are intended as mechanism and materiality checks under illustrative parameter values, not as a calibrated empirical exercise. The primary implementation suggests that joint learning can improve belief accuracy when the ESG rating signal is weak and stock returns carry information about the hidden quality. The ESS-screened materiality-frontier and channel-decomposition experiments indicate that rating-innovation ambiguity is transmitted through rating distortion, value sensitivity $F_{z}$, and filtering-hedging demand. Its benchmark CE effect is small and the channel is not uniformly large. The independent replication adds interval estimates, operational benchmarks, and global parameter uncertainty; it confirms overlapping partial-information outcomes and small absolute effects, with the most stable adverse direction appearing in ES_5_ within the tested scenario box. Low-ESS severe ambiguity cells are interpreted as stress illustrations rather than main evidence. The interior-retention scenario illustrates how underwriting ambiguity can shape model-implied downside capital risk. Finally, the misspecification experiment shows that robust feedback need not maximize reference CE or uniformly dominate a naive rule, but may moderate exposure to an unmodeled systematic rating bias in the tested scenarios.

Several limitations should be kept in view. The benchmark and stress parameters are illustrative rather than calibrated estimates. The drift-distorted ${CE}_{wc}^{dd}$ reported in the numerical section is an approximation to worst-case performance under the specified distortion, not a full empirical evaluation of model uncertainty. Rating-innovation ambiguity is a reduced-form robustness device, not a strategic model of corporate greenwashing or rating-agency behavior; the separate $g_{true}$ exercise is only a fixed-bias stress test. The verification result relies on smoothness, saddle-point attainability, and explicit integrability conditions; the affine sufficient subclass makes these conditions checkable for the candidate strategy but does not remove the need for such regularity assumptions.

These limitations point to several natural extensions. Empirical calibration could use multi-provider ESG rating disagreement, disclosure-related measures, or controversy data to estimate signal noise and systematic rating bias more directly. The portfolio side could be enriched with insurer-specific ESG mandates, regulatory capital constraints, stochastic interest rates, jump risks, or transaction costs. The learning model could also incorporate multiple rating agencies, allowing the insurer to update beliefs from heterogeneous and possibly correlated ESG signals. Such extensions would make the framework closer to institutional practice while preserving the basic distinctions developed here among noisy ESG information, innovation-level model ambiguity, and fixed systematic rating bias.

## Appendix

### A Technical proofs

The statements remain in the main text so that the model logic is visible, while the detailed derivations are collected here. The filtering, separation, Girsanov, and pointwise Hamiltonian arguments are standard adaptations to the present observation system. The coefficient system, constrained feedback, integrability conditions, and conditional verification specialize those tools to this reinsurance–investment model.

#### A.1 Joint Kalman–Bucy filter.

*Proof.* The proof records the specialization of the standard filter to the three-channel observation system used here. The orthogonalization of the brown return only diagonalizes the observation-noise covariance and does not change the original information set. First remove the deterministic intercepts from the observations. The observation equations can be written as


$$\begin{array}{c} \;dY_{t}=b_{0}\;dt+HZ_{t}\;dt+R^{1/2}\;dW_{t}, \end{array}$$ (A.1)

where


$$\begin{array}{c} b_{0}=(\mu_{g}^{0},\mu_{⟂}^{0},\delta_{0}{)}^{⊤},\;H=(\beta_{g},\beta_{⟂},1{)}^{⊤},\;R=diag(\sigma_{g}^{2},\sigma_{b}^{2}s^{2},\sigma_{Y}^{2}), \end{array}$$ (A.2)

and $W=(W_{1},W_{2},W_{4}{)}^{⊤}$. Together with the scalar state equation (3.2), this is a linear Gaussian state-observation system. Hence the conditional law of $Z_{t}$ given ${ℱ}_{t}^{Y}$ is Gaussian and is determined by $\left({\hat{Z}}_{t},P_{t}\right)$.

The innovation vector is the normalized unpredictable part of the observation:


$$\begin{array}{c} \;d{\tilde{W}}_{t}=R^{-1/2}(\;dY_{t}-(b_{0}+H{\hat{Z}}_{t})\;dt),\;\tilde{W}=({\tilde{W}}_{1},{\tilde{W}}_{2},{\tilde{W}}_{4}{)}^{⊤}. \end{array}$$ (A.3)

Its drift conditional on ${ℱ}_{t}^{Y}$ is zero because $𝔼[Z_{t}-{\hat{Z}}_{t}|{ℱ}_{t}^{Y}]=0$, and its conditional quadratic variation is the identity matrix because the observation noise covariance is *R*. By Levy’s characterization, $\tilde{W}$ is a three-dimensional $F^{Y}$-Brownian motion.

For a scalar state with observation matrix *H* and noise covariance *R*, the Kalman gain is


$$\begin{array}{c} K_{t}=P_{t}H^{⊤}R^{-1}=P_{t}\left(\frac{\beta_{g}}{\sigma_{g}^{2}},\frac{\beta_{⟂}}{\sigma_{b}^{2}s^{2}},\frac{1}{\sigma_{Y}^{2}}\right). \end{array}$$ (A.4)

Multiplying the observation residual by this gain and then expressing it through the normalized innovations gives exactly (3.16). The conditional variance loses precision at rate $2\alpha_{Z}P_{t}$ through mean reversion, gains intrinsic state variance $\sigma_{Z}^{2}$, and is reduced by the observation information $P_{t}H^{⊤}R^{-1}HP_{t}=\chi P_{t}^{2}$. This yields (3.17).

It remains to show positivity, boundedness, and uniqueness of $P_{t}$. The right-hand side $f(P)=\sigma_{Z}^{2}-2\alpha_{Z}P-\chi P^{2}$ is locally Lipschitz, so the solution is unique until explosion. Since $f(0)=\sigma_{Z}^{2}>0$, a solution starting from *P*_0_ > 0 cannot cross zero. Let $\bar{P}$ be the positive root of *f*(*P*) = 0:


$$\begin{array}{c} \bar{P}=\frac{-\alpha_{Z}+\sqrt{\alpha_{Z}^{2}+\chi \sigma_{Z}^{2}}}{\chi }>0. \end{array}$$ (A.5)

For $P>\max\{P_{0},\bar{P}\}$, *f*(*P*) < 0. Therefore $P_{t}\leq \max\{P_{0},\bar{P}\}$ on [0,*T*]. The solution is thus positive and bounded, so no finite-time explosion is possible.

#### A.2 Reference-filter separation under joint observations.

*Proof.* The transformation from $\left({\bar{R}}^{g},{\bar{R}}^{b},Y\right)$ to $\left({\bar{R}}^{g},{\bar{R}}^{⟂},Y\right)$ is invertible, so it preserves the market-rating information. Lemma 3.1 gives a finite-dimensional reference filter. Conditional on ${ℱ}_{t}^{Y}$, all conditional moments needed in the stock-return drifts are functions of ${\hat{Z}}_{t}$, because $\mu_{g}$ and $\mu_{b}$ are affine.

The optional projections of the unobservable drifts are therefore $\mu_{g}({\hat{Z}}_{t})$ and $\mu_{b}({\hat{Z}}_{t})$, which gives (3.19)–(3.20). The wealth equation and the filter equation are closed in $\left(X_{t},{\hat{Z}}_{t}\right)$ under the reference model.

After the reference filter has been constructed, the robust evaluation is imposed on the separated innovation dynamics. The adversary changes the drift of the separated innovations, while the state variable remains the reference-filter state $\left(X_{t},{\hat{Z}}_{t}\right)$. Thus (3.31)–(3.32) are the post-separation distorted dynamics used for robust evaluation; Proposition 4.1 itself is only a reference-model separation result and does not bind separation to affine distortions. This is a reference-filter-based separation. It does not assert that the insurer recomputes ${𝔼}_{{\mathbb{Q}}^{\phi }}[Z_{t}|{ℱ}_{t}^{Y}]$ under every distorted measure.

#### A.3 Pointwise worst-case distortion.

*Proof.* The derivatives of (4.3) are


$$\begin{array}{c} V_{x}=-\gamma V,\;V_{z}=VF_{z},\;V_{xz}=-\gamma VF_{z}. \end{array}$$ (A.6)

For fixed *u*, the $\phi_{i}$-dependent part of the Hamiltonian is


$$\begin{array}{c} -(a_{i}(u)V_{x}+\ell_{i,t}V_{z})\phi_{i}-\frac{1}{2}V\kappa_{i}\phi_{i}^{2}. \end{array}$$ (A.7)

Its second derivative is $-V\kappa_{i}>0$, so the minimization is strictly convex. The first-order condition is


$$\begin{array}{c} -(a_{i}(u)V_{x}+\ell_{i,t}V_{z})-V\kappa_{i}\phi_{i}=0. \end{array}$$ (A.8)

Substituting $V_{x}=-\gamma V$ and $V_{z}=VF_{z}$ yields


$$\begin{array}{c} V(\gamma a_{i}(u)-\ell_{i,t}F_{z})-V\kappa_{i}\phi_{i}=0, \end{array}$$ (A.9)

which proves (4.4). Since *a*_4_(*u*) = 0 and $\ell_{3,t}=0$, (4.5) follows. The terms $\phi_{1}^{*}$ and $\phi_{2}^{*}$ contain both the wealth-risk component $\gamma a_{i}(u)$ and the belief-sensitivity component $-\ell_{i,t}F_{z}$, because stock returns are simultaneously investment risks and learning signals.

#### A.4 Reduced HJBI equation and constrained retention.

*Proof.* Substitute (A.6)–(5.2) into the Hamiltonian and divide by *V* < 0. For fixed *u*, minimizing the original Hamiltonian in ϕ is equivalent, after division by the negative value *V*, to maximizing


$$\begin{array}{c} (\gamma a(u)-F_{z}\ell_{t}{)}^{⊤}\phi -\frac{1}{2}\phi^{⊤}\Gamma \phi . \end{array}$$ (A.10)

The maximizer is precisely $\phi^{*}$ in (4.4), and the maximized value is


$$\begin{array}{c} \frac{1}{2}(\gamma a(u)-F_{z}\ell_{t}{)}^{⊤}\Gamma^{-1}(\gamma a(u)-F_{z}\ell_{t}). \end{array}$$ (A.11)

Combining this term with the ordinary diffusion and mixed-derivative terms gives


$$\begin{array}{cc} 0 & =\inf_{\pi_{g},\pi_{b}\in \mathbb{R},\;q\in [0,1]}[F_{t}-\gamma c(\eta -\vartheta )-\gamma m(z{)}^{⊤}u+\alpha_{Z}(\bar{Z}-z)F_{z}+\frac{1}{2}\zeta_{t}F_{zz}\\ & \;+\frac{1}{2}\nu_{t}F_{z}^{2}+\frac{1}{2}\gamma^{2}u^{⊤}Mu-\gamma F_{z}\Lambda_{t}^{⊤}u]. \end{array}$$ (A.12)

The reversal from $\sup_{u}$ to $\inf_{u}$ occurs because *V* < 0. The terms depending on *u* are


$$\begin{array}{c} \frac{1}{2}\gamma^{2}u^{⊤}Mu-\gamma \{m(z)+F_{z}\Lambda_{t}{\}}^{⊤}u. \end{array}$$ (A.13)

Because $\ell_{3,t}=0$ and the underwriting column of Σ is orthogonal to the two stock columns, the *q*-part separates as (5.8). Completing the square in the two stock controls yields the minimizer (5.11) and the minimized stock value


$$\begin{array}{c} -\frac{1}{2}\{m_{\pi }(z)+F_{z}\Lambda_{\pi ,t}{\}}^{⊤}M_{\pi }^{-1}\{m_{\pi }(z)+F_{z}\Lambda_{\pi ,t}\}. \end{array}$$ (A.14)

Substituting this value and $G_{q}^{*}$ gives (5.10).

#### A.5 Positive definiteness and projection inequality.

*Proof.* Since every $\kappa_{i}>0$, $D_{\kappa }≻0$. The matrix Σ has full column rank because its first three rows form an upper triangular matrix with determinant $\sigma_{g}\sigma_{b}sb>0$. Hence, for any nonzero $y\in {\mathbb{R}}^{3}$,


$$\begin{array}{c} y^{⊤}My=(\Sigma y{)}^{⊤}D_{\kappa }(\Sigma y)>0. \end{array}$$ (A.15)

Thus M≻0. The first two columns of Σ are linearly independent because $\sigma_{g}>0$ and $s\sigma_{b}>0$, so the same argument gives $M_{\pi }≻0$.

Let $R=D_{\kappa }^{1/2}\Sigma$ and $h_{t}=D_{\kappa }^{1/2}\ell_{t}$. Then


$$\begin{array}{c} M=R^{⊤}R,\;\Lambda_{t}=R^{⊤}h_{t},\;\nu_{t}=\|h_{t}{\|}^{2}. \end{array}$$ (A.16)

Because *R* has full column rank,


$$\begin{array}{c} \Lambda_{t}^{⊤}M^{-1}\Lambda_{t}=h_{t}^{⊤}R(R^{⊤}R{)}^{-1}R^{⊤}h_{t}. \end{array}$$ (A.17)

The matrix $R(R^{⊤}R{)}^{-1}R^{⊤}$ is the orthogonal projection onto S=Range(R). Therefore the left-hand side is the squared norm of the projection of $h_{t}$ onto S, which is no larger than $\|h_{t}{\|}^{2}=\nu_{t}$. The proof of (5.15) is identical with $R_{\pi }=D_{\kappa }^{1/2}\Sigma_{\pi }$.

#### A.6 Coefficient ODE system.

*Proof.* For (5.16),

$$\begin{array}{c} F_{t}={\dot{A}}_{2}z^{2}+{\dot{B}}_{1}z+\dot{C},\;F_{z}=2A_{2}z+B_{1},\;F_{zz}=2A_{2}. \end{array}$$ (A.18)

The mean-reversion term expands as


$$\begin{array}{cc} \alpha_{Z}(\bar{Z}-z)F_{z} & =\alpha_{Z}(\bar{Z}-z)(2A_{2}z+B_{1})\\ & =-2\alpha_{Z}A_{2}z^{2}+\alpha_{Z}(2\bar{Z}A_{2}-B_{1})z+\alpha_{Z}\bar{Z}B_{1}. \end{array}$$ (A.19)

The filter-variance terms expand as


$$\begin{array}{c} \frac{1}{2}\zeta_{t}F_{zz}=\zeta_{t}A_{2}, \end{array}$$ (A.20)

and


$$\begin{array}{c} \frac{1}{2}\nu_{t}F_{z}^{2}=2\nu_{t}A_{2}^{2}z^{2}+2\nu_{t}A_{2}B_{1}z+\frac{1}{2}\nu_{t}B_{1}^{2}. \end{array}$$ (A.21)

By (5.19),


$$\begin{array}{cc} \frac{1}{2}\{m_{\pi }(z)+F_{z}\Lambda_{\pi ,t}{\}}^{⊤}M_{\pi }^{-1}\{m_{\pi }(z)+F_{z}\Lambda_{\pi ,t}\} & =\frac{1}{2}n_{1}^{⊤}M_{\pi }^{-1}n_{1}z^{2}\\ & \;+n_{1}^{⊤}M_{\pi }^{-1}n_{0}z+\frac{1}{2}n_{0}^{⊤}M_{\pi }^{-1}n_{0}. \end{array}$$ (A.22)

Collecting the coefficients of *z*^2^, *z*, and 1 in (5.10) gives (5.20)–(5.22).

#### A.7 Global solvability of the coefficient system.

*Proof.* Lemma 3.1 implies that $P_{t}$, hence $\ell_{t},\Lambda_{\pi ,t},\nu_{t},\zeta_{t}$, are bounded deterministic functions. Local existence and uniqueness follow because the right-hand sides of (5.20)–(5.22) are locally Lipschitz in $\left(A_{2},B_{1},C\right)$.

It remains to rule out finite-time explosion. Let $G_{\pi }=M_{\pi }^{-1}$. By Lemma 5.2,


$$\begin{array}{c} \Lambda_{\pi ,t}^{⊤}G_{\pi }\Lambda_{\pi ,t}\leq \nu_{t},\;0\leq t\leq T. \end{array}$$ (A.23)

Write the *A*_2_-equation in forward time τ=T−t, and set


$$\begin{array}{c} A(\tau )=A_{2}(T-\tau ),\;t=T-\tau . \end{array}$$ (A.24)

Expanding $n_{1}^{⊤}G_{\pi }n_{1}$ in (5.20) gives


$$\begin{array}{c} n_{1}^{⊤}G_{\pi }n_{1}=m_{\pi ,1}^{⊤}G_{\pi }m_{\pi ,1}+4A_{2}\Lambda_{\pi ,t}^{⊤}G_{\pi }m_{\pi ,1}+4A_{2}^{2}\Lambda_{\pi ,t}^{⊤}G_{\pi }\Lambda_{\pi ,t}. \end{array}$$ (A.25)

Since $\;dA/\;d\tau =-{\dot{A}}_{2}(T-\tau )$, the forward equation is


$$\begin{array}{c} \frac{\;dA}{\;d\tau }=2(\nu_{t}-\Lambda_{\pi ,t}^{⊤}G_{\pi }\Lambda_{\pi ,t})A^{2}-2(\alpha_{Z}+\Lambda_{\pi ,t}^{⊤}G_{\pi }m_{\pi ,1})A-\frac{1}{2}m_{\pi ,1}^{⊤}G_{\pi }m_{\pi ,1}. \end{array}$$ (A.26)

The coefficient of $A^{2}$ is nonnegative by (A.23). Since $A(0)=A_{2}(T)=0$, and the right-hand side of (A.26) at A=0 equals $-\frac{1}{2}m_{\pi ,1}^{⊤}G_{\pi }m_{\pi ,1}\leq 0$, the solution cannot cross from the nonpositive half-line into the positive half-line. Indeed, if the first crossing time existed, continuity would give A=0 at that time while the vector field points weakly toward the nonpositive side. Hence A(τ)≤0 on [0,*T*].

Put Y(τ)=−A(τ)≥0. Define


$$\begin{array}{c} K_{1}=2\sup_{0\leq t\leq T}\left|\alpha_{Z}+\Lambda_{\pi ,t}^{⊤}G_{\pi }m_{\pi ,1}\right|,\;K_{2}=\frac{1}{2}m_{\pi ,1}^{⊤}G_{\pi }m_{\pi ,1}. \end{array}$$ (A.27)

Then $K_{1},K_{2}<\infty$, and (A.26) gives


$$\begin{array}{c} \frac{\;dY}{\;d\tau }=-2(\nu_{t}-\Lambda_{\pi ,t}^{⊤}G_{\pi }\Lambda_{\pi ,t})Y^{2}-2(\alpha_{Z}+\Lambda_{\pi ,t}^{⊤}G_{\pi }m_{\pi ,1})Y+\frac{1}{2}m_{\pi ,1}^{⊤}G_{\pi }m_{\pi ,1}\leq K_{1}Y+K_{2}. \end{array}$$ (A.28)

Gronwall’s inequality gives


$$\begin{array}{c} 0\leq Y(\tau )\leq \left\{ \begin{array}{cc} \frac{K_{2}}{K_{1}}(e^{K_{1}\tau }-1), & K_{1}>0,\\ K_{2}\tau , & K_{1}=0, \end{array}\right.\;0\leq \tau \leq T. \end{array}$$ (A.29)

Thus *A*_2_ is bounded on [0,*T*].

Given bounded *A*_2_, the equation for *B*_1_ is linear in *B*_1_. To see this explicitly,


$$\begin{array}{cc} n_{1}^{⊤}G_{\pi }n_{0} & =(m_{\pi ,1}+2A_{2}\Lambda_{\pi ,t}{)}^{⊤}G_{\pi }(m_{\pi ,0}+B_{1}\Lambda_{\pi ,t})\\ & =(m_{\pi ,1}+2A_{2}\Lambda_{\pi ,t}{)}^{⊤}G_{\pi }m_{\pi ,0}+B_{1}(m_{\pi ,1}+2A_{2}\Lambda_{\pi ,t}{)}^{⊤}G_{\pi }\Lambda_{\pi ,t}. \end{array}$$ (A.30)

There is no $B_{1}^{2}$ term. Hence (5.21) is a scalar linear ODE for *B*_1_ with bounded deterministic coefficients, and Gronwall’s inequality gives a unique bounded solution on [0,*T*]. Finally, (5.22) is an integral equation for *C*. Its right-hand side contains $B_{1}^{2}$, but *B*_1_ is already bounded, so the integrand is bounded and continuous. Therefore *C* is finite and unique on the whole horizon.

#### A.8 Affine closure of feedback controls and distortions.

*Proof.* Since $u(t,z)=u_{0}(t)+U_{1}(t)z$ and a(u)=Σu, the exposure vector satisfies


$$\begin{array}{c} a(u(t,z))=\Sigma u_{0}(t)+\Sigma U_{1}(t)z, \end{array}$$ (A.31)

which is affine in *z*. By (5.16),


$$\begin{array}{c} F_{z}(t,z)=2A_{2}(t)z+B_{1}(t), \end{array}$$ (A.32)

which is also affine. Substituting these two affine functions into (5.23) gives


$$\begin{array}{cc} p_{i}^{u}(t) & =\frac{\gamma (\Sigma u_{0}(t){)}_{i}-\ell_{i,t}B_{1}(t)}{\kappa_{i}}, \end{array}$$ (A.33)

$$\begin{array}{cc} R_{i}^{u}(t) & =\frac{\gamma (\Sigma U_{1}(t){)}_{i}-2\ell_{i,t}A_{2}(t)}{\kappa_{i}}, \end{array}$$ (A.34)

for i=1,...,4. The functions $p^{u},R^{u}$ are deterministic and bounded because $u_{0},U_{1}$ are bounded, $P_{t}$ and $\ell_{t}$ are bounded, and Proposition 5.4 gives bounded $A_{2},B_{1}$. More explicitly, if


$$\begin{array}{c} \kappa_{\min}=\min_{1\leq i\leq 4}\kappa_{i},\;L_{\ell }=\sup_{t}\|\ell_{t}\|,\;L_{A}=\sup_{t}|A_{2}(t)|,\;L_{B}=\sup_{t}|B_{1}(t)|, \end{array}$$ (A.35)

then one may take


$$\begin{array}{c} C_{p}(L_{u})=\frac{\gamma \|\Sigma \|L_{u}+L_{\ell }L_{B}}{\kappa_{\min}},\;C_{R}(L_{u})=\frac{\gamma \|\Sigma \|L_{u}+2L_{\ell }L_{A}}{\kappa_{\min}}, \end{array}$$ (A.36)

up to the harmless equivalence of finite-dimensional vector norms.

For the candidate control, (5.11) and (5.19) give


$$\begin{array}{c} \pi^{*}(t,z)=\frac{1}{\gamma }M_{\pi }^{-1}n_{0}(t)+\frac{1}{\gamma }M_{\pi }^{-1}n_{1}(t)z,\;q^{*}=\Pi_{\left[0,1\right]}(q^{unc}). \end{array}$$ (A.37)

The functions $n_{0},n_{1}$ are bounded because $A_{2},B_{1},\Lambda_{\pi ,t},m_{\pi ,0},m_{\pi ,1}$ are bounded and $M_{\pi }^{-1}$ is constant. Thus $u^{*}\in {𝒰}_{aff}(L_{u}^{*})$ with


$$\begin{array}{c} L_{u}^{*}=\sup_{0\leq t\leq T}\left(\left\|(\begin{array}{c} \gamma^{-1}M_{\pi }^{-1}n_{0}(t)\\ q^{*} \end{array})\right\|+\left\|(\begin{array}{c} \gamma^{-1}M_{\pi }^{-1}n_{1}(t)\\ 0 \end{array})\right\|\right)<\infty . \end{array}$$ (A.38)

Applying the first part of the proof to $u^{*}$ shows that $\phi^{*}$ is affine. Its membership in $\Theta_{aff}^{int}$ is then exactly the integrability requirement verified in Lemma 5.7.

#### A.9 Retention under the interval constraint.

*Proof.* The third column of Σ is $(0,0,b,0{)}^{⊤}$, and $\ell_{3,t}=0$. Therefore the third component of $\Lambda_{t}=\Sigma^{⊤}D_{\kappa }\ell_{t}$ is zero, and the *q*-part of the minimization in (A.12) is independent of $\left(\pi_{g},\pi_{b}\right)$:


$$\begin{array}{c} \min_{0\leq q\leq 1}\left\{\frac{1}{2}\gamma^{2}d_{3}b^{2}q^{2}-\gamma c\vartheta q\right\}. \end{array}$$ (A.39)

This is a strictly convex quadratic. Its unconstrained minimizer solves $\gamma^{2}d_{3}b^{2}q-\gamma c\vartheta =0$, giving $q^{unc}$. The minimizer over the interval [0,1] is the Euclidean projection of this scalar minimizer onto the interval, which is exactly (5.26).

#### A.10 Exponential integrability of affine distortions.

*Proof.* The proof has two steps.

First, prove Novikov under the reference measure ℙ. Under ℙ, the reference belief state satisfies


$$\begin{array}{c} \;d{\hat{Z}}_{s}=\alpha_{Z}(\bar{Z}-{\hat{Z}}_{s})\;ds+\ell_{s}^{⊤}\;d{\tilde{W}}_{s}. \end{array}$$ (A.40)

Because $\ell_{s}$ is deterministic and bounded, ${\hat{Z}}_{s}$ is Gaussian. Its variance $v_{s}^{\mathbb{P}}$, conditional on ${\hat{Z}}_{t}=z$, satisfies


$$\begin{array}{c} \frac{\;dv_{s}^{\mathbb{P}}}{\;ds}=-2\alpha_{Z}v_{s}^{\mathbb{P}}+\|\ell_{s}{\|}^{2}\;v_{t}^{\mathbb{P}}=0. \end{array}$$ (A.41)

Solving this scalar inequality with $\|\ell_{s}\|\leq L_{\ell }$ gives


$$\begin{array}{c} v_{s}^{\mathbb{P}}\leq \frac{L_{\ell }^{2}}{2\alpha_{Z}}\left(1-e^{-2\alpha_{Z}(s-t)}\right)\leq v_{T}^{\mathbb{P}}. \end{array}$$ (A.42)

For a Gaussian random variable *G*, $𝔼e^{\lambda G^{2}}<\infty$ whenever 2λVar(G)<1. Jensen’s inequality therefore yields


$$\begin{array}{c} {𝔼}^{\mathbb{P}}\exp\left(\theta \int_{t}^{T}{\hat{Z}}_{s}^{2}\;ds\right)\leq \frac{1}{T-t}\int_{t}^{T}{𝔼}^{\mathbb{P}}\exp\left(\theta (T-t){\hat{Z}}_{s}^{2}\right)\;ds<\infty \end{array}$$ (A.43)

whenever $2\theta Tv_{T}^{\mathbb{P}}<1$. Since


$$\begin{array}{c} \|\phi (s,{\hat{Z}}_{s}){\|}^{2}\leq 2L_{p}^{2}+2L_{R}^{2}{\hat{Z}}_{s}^{2}, \end{array}$$ (A.44)

condition (5.31) implies


$$\begin{array}{c} {𝔼}^{\mathbb{P}}\exp\left(\frac{1}{2}\int_{0}^{T}\|\phi (s,{\hat{Z}}_{s}){\|}^{2}\;ds\right)<\infty . \end{array}$$ (A.45)

Thus Novikov’s condition holds under ℙ, and ${\mathbb{Q}}^{\phi }$ is well defined.

We next record how ${\bar{\theta }}_{\phi }$ can be constructed. Let


$$\begin{array}{c} L_{A}=\sup_{t}|A_{2}(t)|,\;L_{B}=\sup_{t}|B_{1}(t)|,\;L_{C}=\sup_{t}|C(t)|,\;L_{m}=\|m_{0}\|+\|m_{1}\|,\;L_{\Sigma }=\|\Sigma \|. \end{array}$$ (A.46)

For $F(t,z)=A_{2}(t)z^{2}+B_{1}(t)z+C(t)$,


$$\begin{array}{c} |F(t,z)|\leq C_{F}(1+z^{2}),\;C_{F}=L_{A}+L_{B}+L_{C}. \end{array}$$ (A.47)

For $u\in {𝒰}_{aff}(L_{u})$ and ϕ(t,z)=p(t)+R(t)z,


$$\begin{array}{c} \|u(t,z)\|\leq L_{u}(1+|z|),\;\|\phi (t,z)\|\leq C_{\phi }(1+|z|),\;C_{\phi }=L_{p}+L_{R}. \end{array}$$ (A.48)

Since $m(z)=m_{0}+m_{1}z$ and a(u)=Σu, there are finite constants $C_{X}$ and $C_{\Sigma }$, depending only on $L_{u},C_{\phi },L_{m},L_{\Sigma },c,\eta ,\vartheta$, such that


$$\begin{array}{cc} \left|m(z{)}^{⊤}u(t,z)-a(u(t,z){)}^{⊤}\phi (t,z)+c(\eta -\vartheta )\right| & \leq C_{X}(1+z^{2}), \end{array}$$ (A.49)

$$\begin{array}{cc} \left\|a(u(t,z))\right\| & \leq C_{\Sigma }(1+|z|). \end{array}$$ (A.50)

Equivalently, under ${\mathbb{Q}}^{\phi }$,


$$\begin{array}{cc} \;dX_{s} & =\mu_{X}(s,{\hat{Z}}_{s})\;ds+\sigma_{X}(s,{\hat{Z}}_{s}{)}^{⊤}\;d{\tilde{W}}_{s}^{\phi }, \end{array}$$ (A.51)

$$\begin{array}{cc} \mu_{X}(s,z) & =m(z{)}^{⊤}u(s,z)+c(\eta -\vartheta )-a(u(s,z){)}^{⊤}\phi (s,z), \end{array}$$ (A.52)

$$\begin{array}{cc} \sigma_{X}(s,z) & =a(u(s,z)), \end{array}$$ (A.53)

and (A.49)–(A.50) read


$$\begin{array}{c} |\mu_{X}(s,z)|\leq C_{X}(1+z^{2}),\;\|\sigma_{X}(s,z)\|\leq C_{\Sigma }(1+|z|). \end{array}$$ (A.54)

For any stopping time τ≤T,


$$\begin{array}{c} X_{\tau }=x+\int_{t}^{\tau }\mu_{X}(s,{\hat{Z}}_{s})\;ds+M_{\tau }^{X},\;M_{\tau }^{X}=\int_{t}^{\tau }\sigma_{X}(s,{\hat{Z}}_{s}{)}^{⊤}\;d{\tilde{W}}_{s}^{\phi }. \end{array}$$ (A.55)

Hence


$$\begin{array}{c} -\gamma X_{\tau }\leq C_{0}+\gamma C_{X}\int_{t}^{T}(1+{\hat{Z}}_{s}^{2})\;ds-\gamma M_{\tau }^{X}, \end{array}$$ (A.56)

where *C*_0_ depends on $x,T,\gamma ,C_{X}$. Moreover,


$$\begin{array}{c} \langle M^{X}\rangle_{\tau }=\int_{t}^{\tau }\|\sigma_{X}(s,{\hat{Z}}_{s}){\|}^{2}\;ds\leq 2C_{\Sigma }^{2}\int_{t}^{T}(1+{\hat{Z}}_{s}^{2})\;ds. \end{array}$$ (A.57)

After localization,


$$\begin{array}{c} \exp\left(-2\gamma M_{\tau }^{X}-2\gamma^{2}\langle M^{X}\rangle_{\tau }\right) \end{array}$$ (A.58)

has expectation at most one. Therefore, by Cauchy–Schwarz and (A.57), for any η>0,


$$\begin{array}{c} {𝔼}^{{\mathbb{Q}}^{\phi }}\exp\left(-\gamma M_{\tau }^{X}+\eta \int_{t}^{T}(1+{\hat{Z}}_{s}^{2})\;ds\right)\leq {\left[{𝔼}^{{\mathbb{Q}}^{\phi }}\exp\left((2\eta +4\gamma^{2}C_{\Sigma }^{2})\int_{t}^{T}(1+{\hat{Z}}_{s}^{2})\;ds\right)\right]}^{1/2}. \end{array}$$ (A.59)

Combining (A.56) and (A.59) shows that the family $\{\exp(-\gamma X_{\tau_{n}}){\}}_{n}$ is controlled in expectation by


$$\begin{array}{c} K_{1}{𝔼}^{{\mathbb{Q}}^{\phi }}\exp\left(\theta_{1}\int_{t}^{T}(1+{\hat{Z}}_{s}^{2})\;ds\right) \end{array}$$

for finite constants $K_{1},\theta_{1}$ determined by $C_{X},C_{\Sigma },\gamma ,T,x$.

Since *F*(*t*,*z*) has quadratic growth,


$$\begin{array}{c} \left|V(\tau ,X_{\tau },{\hat{Z}}_{\tau })|=\exp[-\gamma X_{\tau }+F(\tau ,{\hat{Z}}_{\tau })\right] \end{array}$$ (A.60)

is controlled, again by Hölder’s inequality, by an exponential moment of ${\hat{Z}}_{\tau }^{2}+\int_{t}^{T}{\hat{Z}}_{s}^{2}\;ds$. The running penalty satisfies


$$\begin{array}{c} \left|V(s,X_{s},{\hat{Z}}_{s})\phi (s,{\hat{Z}}_{s}{)}^{⊤}\Gamma \phi (s,{\hat{Z}}_{s})\right|\leq \|\Gamma \|\;|V(s,X_{s},{\hat{Z}}_{s})|\;C_{\phi }^{2}(1+{\hat{Z}}_{s}^{2}), \end{array}$$ (A.61)

and the polynomial factor is absorbed into a slightly larger quadratic exponential because


$$\begin{array}{c} 1+z^{2}\leq C_{\varepsilon }e^{\varepsilon z^{2}},\;\varepsilon >0. \end{array}$$

Finally,


$$\begin{array}{c} V_{x}=-\gamma V,\;V_{z}=VF_{z},\;|F_{z}(t,z)|\leq C(1+|z|), \end{array}$$ (A.62)

so the quadratic variations of the stochastic integrals in Ito’s formula, whose integrands contain $V_{x}a(u)$ and $V_{z}\ell$, are bounded by |*V*|^2^ times a polynomial in $1+|{\hat{Z}}_{s}|$. This polynomial is absorbed by the same exponential enlargement. Consequently ${\bar{\theta }}_{\phi }$ can be chosen large enough, depending only on


$$\begin{array}{c} \gamma ,\Gamma ,T,C_{X},C_{\Sigma },C_{F},C_{\phi },L_{\ell } \end{array}$$

and the affine feedback and distortion bounds, to control simultaneously the stopped terminal value, the running penalty, the stochastic-integral quadratic variations, and the stochastic exponential generated by *X*. This gives the domination form (5.34); it is not an additional abstract assumption beyond these explicit bounds and the inequality (5.32).

Second, work under the already-defined measure ${\mathbb{Q}}^{\phi }$. The belief process has affine dynamics


$$\begin{array}{c} \;d{\hat{Z}}_{s}=\left[\alpha_{Z}\bar{Z}-\ell_{s}^{⊤}p(s)+(-\alpha_{Z}-\ell_{s}^{⊤}R(s)){\hat{Z}}_{s}\right]\;ds+\ell_{s}^{⊤}\;d{\tilde{W}}_{s}^{\phi }. \end{array}$$ (A.63)

Its mean is bounded on [*t*,*T*] by Gronwall’s inequality because the affine drift coefficients are bounded. Its variance $v_{s}^{\phi }$ satisfies


$$\begin{array}{c} \frac{\;dv_{s}^{\phi }}{\;ds}=2[-\alpha_{Z}-\ell_{s}^{⊤}R(s)]v_{s}^{\phi }+\|\ell_{s}{\|}^{2}\leq 2L_{b}^{\phi }v_{s}^{\phi }+L_{\ell }^{2},\;v_{t}^{\phi }=0, \end{array}$$ (A.64)

so $\sup_{t\leq s\leq T}v_{s}^{\phi }\leq v_{T}^{\phi }$. Apply Jensen’s inequality to the probability measure on {*}∪[t,T] that assigns mass 1/(*T* + 1) to the atom * and Lebesgue density 1/(*T* + 1) on [*t*,*T*], with ${\hat{Z}}_{*}={\hat{Z}}_{\tau }$. The Gaussian-square estimate then gives


$$\begin{array}{c} {𝔼}^{{\mathbb{Q}}^{\phi }}\exp\left(\theta \left({\hat{Z}}_{\tau }^{2}+\int_{t}^{T}{\hat{Z}}_{s}^{2}\;ds\right)\right)<\infty \;\text{for every }\tau \leq T\text{ whenever}\;2\theta (T+1)v_{T}^{\phi }<1. \end{array}$$ (A.65)

Condition (5.32) and the domination (5.34) then imply integrability of the terminal payoff, running penalty, and stochastic-integral quadratic variations. The localized stochastic integrals are therefore uniformly integrable, so their expectations vanish after localization and passage to the limit.

#### A.11 Conditional verification theorem.

*Proof.* Fix $u\in A^{V}(t,x,z)$. By definition, the induced pointwise minimizer $\phi^{u}$ belongs to $\Theta^{V}(t,x,z;u)$. Let $\tau_{n}$ be a localization sequence increasing to *T* such that the stochastic integrals obtained from Ito’s formula applied to $V(s,X_{s},{\hat{Z}}_{s})$ are true martingales on $\left[t,\tau_{n}\right]$. Under ${\mathbb{Q}}^{\phi^{u}}$,


$$\begin{array}{cc} & {𝔼}_{t,x,z}^{{\mathbb{Q}}^{\phi^{u}}}[V(\tau_{n},X_{\tau_{n}},{\hat{Z}}_{\tau_{n}})+\int_{t}^{\tau_{n}}\left(-\frac{1}{2}V\{\phi_{s}^{u}{\}}^{⊤}\Gamma \phi_{s}^{u}\right)\;ds]\\ & \;=V(t,x,z)+{𝔼}_{t,x,z}^{{\mathbb{Q}}^{\phi^{u}}}\int_{t}^{\tau_{n}}H(s,X_{s},{\hat{Z}}_{s},u_{s},\phi_{s}^{u})\;ds. \end{array}$$ (A.66)

The martingale term has zero expectation by localization. Using (5.36), letting n→∞ by Assumption 3.4, and applying the terminal condition *V*(*T*,*x*,*z*) = *U*(*x*) gives


$$\begin{array}{c} J(t,x,z;u,\phi^{u})\leq V(t,x,z). \end{array}$$ (A.67)

Thus, for every $u\in A^{V}(t,x,z)$,


$$\begin{array}{c} \inf_{\phi \in \Theta^{V}(t,x,z;u)}J(t,x,z;u,\phi )\leq J(t,x,z;u,\phi^{u})\leq V(t,x,z), \end{array}$$ (A.68)

and hence


$$\begin{array}{c} \sup_{u\in A^{V}(t,x,z)}\inf_{\phi \in \Theta^{V}(t,x,z;u)}J(t,x,z;u,\phi )\leq V(t,x,z). \end{array}$$ (A.69)

For the candidate $u^{*}$, fix any $\phi \in \Theta^{V}(t,x,z;u^{*})$. Applying Ito’s formula under ${\mathbb{Q}}^{\phi }$ to the stopped process and using (5.37) gives


$$\begin{array}{c} {𝔼}_{t,x,z}^{{\mathbb{Q}}^{\phi }}[V(\tau_{n},X_{\tau_{n}},{\hat{Z}}_{\tau_{n}})+\int_{t}^{\tau_{n}}\left(-\frac{1}{2}V\phi_{s}^{⊤}\Gamma \phi_{s}\right)\;ds]\geq V(t,x,z). \end{array}$$ (A.70)

Assumption 3.4 again gives uniform integrability of the stopped terminal values, the running penalty, and the localized stochastic integrals. Passing n→∞ and using *V*(*T*,*x*,*z*) = *U*(*x*) yields $J(t,x,z;u^{*},\phi )\geq V(t,x,z)$. Therefore


$$\begin{array}{c} \inf_{\phi \in \Theta^{V}(t,x,z;u^{*})}J(t,x,z;u^{*},\phi )\geq V(t,x,z). \end{array}$$ (A.71)

Since $u^{*}\in A^{V}(t,x,z)$, (A.69) and (A.71) imply the claimed equality. Taking $\phi =\phi^{*}$ in the lower inequality and using (5.35) gives the saddle-point conclusion.

#### A.12 Checkable sufficient admissibility conditions.

*Proof.* The stock component of $u^{*}$ is affine in *z* because $m_{\pi }(z)$ and $F_{z}(t,z)=2A_{2}(t)z+B_{1}(t)$ are affine in *z*, while $M_{\pi }^{-1}$ and $\Lambda_{\pi ,t}$ are deterministic. The retention component is the constant projection $q^{*}=\Pi_{\left[0,1\right]}(q^{unc})$, so $q^{*}\in [0,1]$. Hence $u^{*}$ has at most linear growth in the filtered state.

Substituting this affine feedback into (4.4) shows that $\phi^{*}(t,z)$ is affine in *z*. Condition (I) in Lemma 5.7 gives the Novikov-side exponential moment, finite distortion cost, and class-D domination needed in Assumption 3.4. Therefore the Girsanov density is a true martingale, the separated state system is well posed, and the stopped terminal value, running penalty, and localized stochastic integrals satisfy the required uniform-integrability conditions. Thus $\phi^{*}\in \Theta^{V}(t,x,z;u^{*})$, and by the definition (3.42), $u^{*}\in A^{V}(t,x,z)$.

#### A.13 Model reductions and limiting cases.

*Proof.* Each case follows by applying the stated restriction to the filter, the reduced PDE, and the feedback rule.

*No stock-return* learning. If $\beta_{g}=0$ and $\beta_{b}=0$, then $\beta_{⟂}=0$, stock returns provide no information about $Z_{t}$, and


$$\begin{array}{c} \chi =\frac{1}{\sigma_{Y}^{2}},\;\ell_{t}=(0,0,0,P_{t}/\sigma_{Y}{)}^{⊤}. \end{array}$$ (A.72)

Since $\Sigma^{⊤}D_{\kappa }\ell_{t}=0$, the filtering hedging demand disappears and the structure reduces to the earlier rating-only learning model.

*No ESG rating information:* If $\sigma_{Y}\to \infty$, then the rating channel contributes neither to χ nor to $\ell_{t}$. Filtering remains possible whenever $\beta_{g}$ or $\beta_{⟂}$ is nonzero, because stock returns are still noisy observations of the hidden green quality.

*No rating-innovation ambiguity.* If $\kappa_{4}\to \infty$, then $\phi_{4}^{*}\to 0$. Rating uncertainty remains through $\sigma_{Y}$ and the filter term $P_{t}/\sigma_{Y}$; only adversarial drift distortion of the ESG rating innovation is removed.

*Full-information benchmark.* Full information should be treated as a separate benchmark in which $Z_{t}$ is observed directly by the insurer. It is not merely the informal statement $\sigma_{Y}\to 0$, because that is a singular observation limit. In the full-information benchmark one sets ${\hat{Z}}_{t}=Z_{t}$, removes filtering error $P_{t}$, and writes the HJBI with the observed state dynamics (3.2).

*No brown stock.* If $\pi_{b}=0$, the insurer invests only in the green stock and chooses reinsurance. If the brown stock price remains publicly observable, ${\bar{R}}^{⟂}$ may still enter the observation system and improve learning even though the brown stock is not traded. If the brown stock is neither traded nor observable, then ${\bar{R}}^{⟂}$ must be removed from Y, and the information intensity becomes $\beta_{g}^{2}/\sigma_{g}^{2}+1/\sigma_{Y}^{2}$.

*No reinsurance or no underwriting ambiguity.* If reinsurance is not allowed, set *q* = 1 and optimize only $\left(\pi_{g},\pi_{b}\right)$. If $\kappa_{3}\to \infty$, then $d_{3}\to 1$, and the unconstrained retention becomes $c\vartheta /(\gamma b^{2})$. With the practical constraint q∈[0,1], the projected rule (5.26) applies.

### Supporting information

S1 FileS1 Fig. Interior-retention warning-probability surface.Two-dimensional diagnostic surface over underwriting ambiguity $\kappa_{3}$ and rating-innovation ambiguity $\kappa_{4}$. **S2 Fig. Learning comparison on original scales.** CE, warning probability, VaR_5_, ES_5_, and filter MSE without axis zooming. **S3 Fig. Systematic rating-bias comparison on original scales.** Strategy metrics over the $g_{true}$ grid without axis zooming. **S4 Fig. Global parameter-uncertainty analysis.** Paired finite-$\kappa_{4}$ minus weak-ambiguity changes over the 40-point Latin hypercube. **S1 Table. Weak-rating replication with uncertainty intervals.** Bootstrap intervals are reported for CE, VaR_5_, and ES_5_, and Wilson intervals for warning probability. **S2 Table. Operational-benchmark comparison.** Dynamic robust, static-initial-policy, and insurance-only results with uncertainty intervals. **S3 Table. Global parameter uncertainty.** Across-design ranges and adverse fractions for CE, warning probability, VaR_5_, and ES_5_. **S1 Code. Reproducibility package.** MATLAB and Python source, parameter definitions, random seeds, generated CSV files, machine-readable manifest, and README documentation.(ZIP)
