# Supplementary material for: Robust optimal reinsurance and ESG investment under rating-innovation ambiguity and ESG rating uncertainty
Source: PLoS One. 2026 Nov 2;21(11):e0354616. doi: 10.1371/journal.pone.0354616 (PMC13726995; doi:10.1371/journal.pone.0354616)
Supplement: Supplementary file 2 [file pone.0354616.s003.pdf]

Response to the Academic Editor and Reviewers

Manuscript ID: PONE-D-26-34087

Revised title: Robust optimal reinsurance and ESG investment under rating-innovation ambiguity and ESG rating uncertainty

Dear Dr. Mazzoccoli and Reviewers,

Thank you for the careful and constructive assessment. We have substantially revised the manuscript. The central conceptual change is that the manuscript no longer uses "greenwashing ambiguity" as a common label for different mathematical objects. It now distinguishes rating noise σ_Y, endogenous rating-innovation ambiguity ϕ_4, and the out-of-model systematic rating-bias stress g_{true}. We also added a preliminaries and contribution-map section, moved detailed proofs to Appendix A, formally defined the materiality frontier, expanded the numerical uncertainty analysis, added transparent benchmarks and original-scale plots, and independently implemented the new numerical checks in Python.

The revision package contains an unmarked manuscript, a marked manuscript in which additions and modifications are red, the MATLAB and Python reproducibility code, generated CSV outputs, and Supporting Figs S1–S4 and Tables S1–S3. The responses below follow the numbering of the decision letter.

1 Journal requirements

E1. PLOS ONE style and file naming

> ***Comment.*** *Please ensure that the manuscript meets PLOS ONE's style requirements, including those for file naming.*

Response. The revised manuscript has been reformatted using the current PLOS LaTeX structure. It has a PLOS-style title/affiliation block, line numbering, PLOS figure naming ("Fig"), a complete Data and Code Availability statement, a Competing Interests statement, numbered tables, and a Supporting Information inventory. The clean and marked files use descriptive upload names, and figure files are retained as separate PDF/PNG artifacts. Table source has been inlined so that the clean manuscript is a single cohesive TeX file.

Location in revised manuscript. Title page, throughout the manuscript, and the final Supporting Information and Data and Code Availability sections.

E2. Code sharing

> ***Comment.*** *Author-generated code underlying the findings should be available without restriction and documented for reproducibility and reuse.*

Response. We agree. The complete MATLAB workflow is provided in `numerics_matlab`, with `run_all.m` as its entry point. For this revision, we added an independently written Python 3.12

workflow in `numerics_python`, with `run_revision_analysis.py` as its entry point. The Python package includes a requirements file, documented random seeds, simulation sizes, bootstrap settings, the Latin-hypercube design, CSV outputs, and a machine-readable manifest. The accompanying reproducibility README explains both workflows. These author-generated files are designated as S1 Code and will be available without restriction with the article.

Location in revised manuscript. Numerical Analysis: "Numerical implementation and benchmark design"; Supporting Information; Data and Code Availability; S1 Code package.

E3. PLOS LaTeX template

> ***Comment.*** *Please update the submission to use the PLOS LaTeX template.*

Response. The manuscript source has been rebuilt on the current PLOS LaTeX template structure (version 3.8, April 2026). We retained the official template's page geometry, line-numbering, hyperlink, microtype, caption, and footer conventions. The manuscript tables are contained in the main TeX source, and publication figure files are supplied separately.

Location in revised manuscript. Revised manuscript preamble and title page.

E4. Funding text in the manuscript and online Funding Statement

> ***Comment.*** *Please remove funding-related text from the manuscript and provide the amended Funding Statement in the cover letter.*

Response. All funding-related text has been removed from the revised manuscript. For the cover letter and online form, the authors confirm the following Funding Statement:

> "Jing Fu gratefully acknowledges the support from Yunnan Provincial Department of Education Scientific Research Fund Project (Grant No.: 2026J1134), Guoyong Zhou gratefully acknowledges the support from Leshan Philosophy and Social Science Planning Project (Grant No. SKL2025D31), and Leshan Normal University Scientific Star-up Project for Introducing High-level Talents (Grants No. RC2025012)."

Location in revised manuscript. Revised title page. The former manuscript Funding section has been deleted; the statement above is for the cover letter/online form only.

E5. Role of the funders

> ***Comment.*** *Please state the funders' role. If the funders had no role, use the sentence provided in the decision letter.*

Response. The proposed cover-letter statement is:

> "The funders had no role in study design, data collection and analysis, decision to publish, or preparation of the manuscript."

E6. Salary support and online author-contribution roles

> ***Comment.*** *Because an author may be affiliated with a funding organization, review the online author-contribution roles and confirm the journal's salary-support statement with the relevant initials.*

Response. The author-contribution roles will be reviewed and updated in the online form so that each author's specific work is accurately recorded. We have not inserted author initials into the journal's salary-support sentence because the supplied information does not establish whether any grant provided salary support.

E7. Data Availability Statement

> ***Comment.*** *Please provide a complete Data Availability Statement with access information; if only submission files are used, state that all data are in the manuscript and/or Supporting Information.*

Response. We have added the requested unrestricted-availability statement and clarified the provenance of every numerical result. All observations are simulated; no human, animal, proprietary, or restricted third-party data are used. The online form should state: "All data are in the manuscript and/or supporting information files." The manuscript further identifies the MATLAB and Python code paths, generated CSV files, seeds, and manifest.

Location in revised manuscript. Data and Code Availability; Supporting Information; reproducibility README.

E8. Citation of Table 4

> ***Comment.*** *Please ensure that Table 4 is referred to in the manuscript text.*

Response. Corrected. The text now explicitly identifies Table 4 as the materiality summary and cites it immediately after the formal materiality definition.

Location in revised manuscript. Numerical Analysis: "ESS-screened rating-innovation ambiguity materiality frontier," following Eq. (materiality-definition).

E9. Reviewer-suggested citations

> ***Comment.*** *Evaluate any recommended publications for relevance; citation is not mandatory unless directed by the editor.*

Response. No reviewer supplied a list of mandatory references. We reviewed the existing citations while revising the introduction and retained sources directly relevant to ESG-rating disagreement, greenwashing motivation, partial-information control, robustness, and insurance applications. We did not add citations solely to satisfy a non-specific request.

2 Reviewer 1

R1.1. Source and motivation of numerical parameters; real-data description

> ***Comment.*** *The origin of the numerical parameter values was unclear. Please motivate the choices, and add a data section if real data were used.*

Response. We agree that the original wording was insufficient. The revised numerical-method subsection now explains the design logic for the one-year horizon, 252-step grid, 50,000-path MATLAB experiments, moderate annualized asset inputs, hidden-state and signal parameters, insurance parameters, and ambiguity ranges. It explicitly states that the choices are intended to produce nondegenerate learning, an informative constrained-retention region, and ESS-screened ambiguity scenarios. They are illustrative scenario inputs, not fitted estimates.

No real-world data were used. Every observation was simulated from the model. We therefore did not create an empirical data section that could incorrectly imply calibration. Instead, we added an explicit simulated-data paragraph and a complete Data and Code Availability statement. The Python sensitivity analysis also varies eight inputs over documented ranges so the conclusions are not tied to a single parameter vector.

Location in revised manuscript. Numerical Analysis: "Numerical implementation and benchmark design"; "Independent uncertainty and operational-benchmark checks"; Data and Code Availability.

3 Reviewer 2

R2.1. Preliminaries for unfamiliar readers

> ***Comment.*** *Please add a preliminaries section for readers who are not familiar with the topics.*

Response. We added a new section entitled "Preliminaries and contribution map." It introduces the Kalman–Bucy filter, Girsanov drift distortions, and the HJBI equation in plain terms; distinguishes standard methodology from the model-specific contributions; and includes Box 1 mapping $\phi_1, \dots, \phi_4$ to their innovation, wealth, and belief effects. We also moved detailed proofs to Appendix A so that readers can first follow the economic and mathematical structure.

Location in revised manuscript. Section 2, "Preliminaries and contribution map"; Appendix A.

4 Reviewer 3: major comments

R3.1. Distinguish model ambiguity from systematic ESG-rating bias

> ***Comment.*** *The manuscript used "greenwashing" for both the adversarial robust-control distortion ϕ_4 and the actual misspecification g^{true}, although they represent different mechanisms.*

Response. We agree and made this distinction a central organizing principle of the revision. We now reserve:

- **rating noise** for σ_Y, the diffusion-scale imprecision of the observed rating;
- **rating-innovation ambiguity** for ϕ_4 (governed by κ_4), the endogenous worst-case drift distortion inside the robust control problem; and
- **systematic rating-bias misspecification** for g_{true}, the fixed out-of-model drift offset used only in a numerical stress test.

The title, abstract, introduction, model interpretation, numerical subsection names, figure captions, numerical summary, and conclusion have been revised consistently. We explicitly state that ϕ_4 is motivated by credibility concerns but is not an observed or estimated act of corporate greenwashing, and that the g_{true} experiment is descriptive stress testing rather than empirical identification.

Location in revised manuscript. Title and Abstract; Introduction; Preliminaries; Model Formulation; Numerical Analysis: "Rating-innovation ambiguity channel decomposition" and "systematic rating-bias misspecification"; Conclusion.

R3.2. Financial risk versus informational ambiguity

> ***Comment.*** *The economic interpretation of the four ambiguity components is not transparent. The first two distortions affect both returns and beliefs, so the framework does not fully disentangle market and information-quality uncertainty.*

Response. We agree with the reviewer's characterization and no longer imply a full disentanglement. The new contribution-map display (Box 1) states: ϕ_1 and ϕ_2 directly affect both wealth and belief updating because stock-return innovations are simultaneously payoff and information shocks; ϕ_3 affects wealth through underwriting risk but not beliefs; and ϕ_4 affects beliefs through the rating innovation but not wealth diffusion directly. The text explicitly calls the double role of ϕ_1 and ϕ_2 an intentional consequence of joint learning and a limitation on economic separation.

Location in revised manuscript. Section 2, Box 1, "Economic interpretation of the four robust-control distortions"; Model Formulation: ambiguity specification; Analytical Discussion.

R3.3. Numerical evidence, uncertainty, benchmarks, and economic claims

> ***Comment.*** *The numerical work mainly validates a mechanism. Effects are often small, confidence intervals are missing, sensitivity is local, comparisons are mainly internal, and explanatory interpretation should be separated from quantitative economic relevance.*

Response. We substantially expanded and reframed the numerical analysis.

1. **Uncertainty intervals.** An independent Python replication uses 12,000 paths and 300 bootstrap resamples for CE, VaR_5, and ES_5, with Wilson intervals for warning probability. For

example, joint-learning CE is 10.204 [10.192, 10.217], rating-only CE is 10.205 [10.192, 10.217], and stock-only CE is 10.203 [10.192, 10.215]. The overlap is now discussed directly and rules out a claim of clear welfare dominance.

2. **Scope of interval reporting.** S1 and S2 Tables contain intervals for the principal cross-strategy comparisons. We now state explicitly that main-text Tables 3–7 are mechanism, frontier, sensitivity, or numerical-diagnostic summaries rather than a complete collection of inferential tables.

3. **Broader parameter uncertainty.** A reproducible 40-point Latin hypercube varies eight inputs: σ_Y, α_Z, P_0, σ_Z, β_g, $\beta_\perp$, κ_4, and γ, using 2,500 paired paths per design. Median CE and warning changes are essentially zero. ES_5 is adverse in 97.5% of the designs, but its absolute range is small, approximately $[-7.31 \times 10^{-4}, -1.9 \times 10^{-7}]$.

4. **Transparent operational benchmarks.** We added a fixed initial robust allocation and an insurance-only policy. Dynamic robust and static CE are nearly identical (both 10.161 to three decimals). Insurance-only has lower CE (10.097) but better VaR_5 and ES_5, demonstrating that no strategy uniformly dominates across reward and downside metrics. Because no real dataset is used, we do not label these as empirical external benchmarks; doing so would overstate the evidence.

5. **Balanced plots.** Original-scale learning and rating-bias plots are now S2 and S3 Figs, alongside the zoomed diagnostic panels.

6. **Cautious interpretation.** The abstract, numerical summary, and conclusion now distinguish mechanism existence from calibrated economic relevance. We state that the tested effects are small, scenario-dependent, and not population estimates.

Location in revised manuscript. Numerical Analysis: implementation/design, value of joint learning, independent Python checks, numerical summary; S1–S3 Tables and S2–S4 Figs; Abstract and Conclusion.

R3.4. Formal definition of the materiality frontier

> ***Comment.*** *Please define materiality, justify the criterion, and discuss alternative definitions.*

Response. We added a formal, operational definition. A finite-κ_4 cell is material only if $\mathrm{ESS}/n \geq 0.05$ and at least one transparent condition holds:

$$\Delta p_{\mathrm{warn}} > 0.001 \text{ with } |t_{\mathrm{warn}}| \geq 2, \quad \Delta\mathrm{VaR}_5 < -0.002, \quad \Delta\mathrm{ES}_5 < -0.002,$$

or $\Delta\mathrm{CE}^{dd}_{\mathrm{wc}} < -0.001$ with $t_{\mathrm{CE}} \leq -2$. We explain that these thresholds are display rules on the illustrative wealth scale, not empirical welfare cutoffs. We also clarify the apparent asymmetry: paired-path t-screens are available for warning probability and CE in the grid experiment, whereas the VaR_5 and ES_5 rules are economic-magnitude cutoffs rather than hypothesis tests; bootstrap uncertainty for the principal quantile and tail comparisons is reported separately in S1 and S2 Tables. The manuscript expressly cautions that the frontier rule does not establish statistical

distinguishability for every tail-risk cell. We also discuss warning-only, tail-only, CE-only, relative-change, and stricter-ESS alternatives and provide the component metrics so readers can apply other criteria. Table 4 is now explicitly cited.

Location in revised manuscript. Numerical Analysis: “ESS-screened rating-innovation ambiguity materiality frontier,” Eq. (materiality-definition) and Table 4.

R3.5. Standard results versus original contributions

> ***Comment.*** *Move proofs of standard filtering, separation, Girsanov, and pointwise Hamiltonian results to an appendix, and make methodological novelty clearer.*

Response. We restructured the theory accordingly. The theorem and proposition statements remain in the main text, but detailed derivations are collected in Appendix A. The new preliminaries section and the introduction explicitly identify the Kalman–Bucy formula, Girsanov transformation, separation argument, and quadratic Hamiltonian completion as standard adaptations. The manuscript-specific content is identified as the joint wealth-belief innovation structure, the mixed derivative and filtering-hedging demand, the interval-constrained retention rule within the robust HJBI, the coefficient system, and the conditional verification for this model.

Location in revised manuscript. Introduction; Section 2, “Preliminaries and contribution map”; Appendix A, “Technical proofs.”

5 Reviewer 3: minor comments

R3.m1. Interpretation of $\mathbb{P}$

> ***Comment.*** *Readers may interpret $\mathbb{P}$ as risk neutral; state explicitly that it is physical/reference.*

Response. Corrected. The model now states at first use that $\mathbb{P}$ is the physical reference probability measure, not a risk-neutral pricing measure.

Location in revised manuscript. Model Formulation: opening paragraph.

R3.m2. Roles of the Brownian motions

> ***Comment.*** *Introduce W_1, W_2, W_3, W_4 more explicitly and describe their roles.*

Response. Corrected. The manuscript now introduces $W_0, \ldots, W_4$ together and assigns fixed roles: latent factor, green-return innovation, orthogonalized brown-return innovation, underwriting risk, and ESG-rating innovation.

Location in revised manuscript. Model Formulation: hidden green quality and joint observations.

R3.m3. Uniform Brownian-motion notation

> ***Comment.*** *The manuscript mixes B^Z with W_i; adopt one notation.*

Response. Corrected. The latent-state Brownian motion B^Z has been renamed W_0, so the manuscript consistently uses $W_0, \dots, W_4$.

Location in revised manuscript. Throughout the model and Appendix A.

R3.m4. Repeated discussion of the reference filter

> ***Comment.*** *Consolidate repeated explanations that filtering is not recomputed under the distorted measure.*

Response. We consolidated this explanation in the ambiguity/separation discussion and removed repeated interpretations. The revised text states once that the reference filter is the observable state used in the separated robust problem and explains the role of the distortions without suggesting repeated nonlinear refiltering under each candidate measure.

Location in revised manuscript. Model Formulation: ambiguity specification and separation; standard proof in Appendix A.

R3.m5. Original-scale plots

> ***Comment.*** *Complement highly zoomed axes with plots on the original scale.*

Response. Added. S2 Fig reports the learning comparison on original scales, and S3 Fig reports the systematic rating-bias comparison on original scales. The main-text captions now direct the reader to these figures and state that zooming is used only to show ordering, not magnitude.

Location in revised manuscript. Value of joint learning; systematic rating-bias misspecification; S2 and S3 Figs.

R3.m6. Monte Carlo uncertainty

> ***Comment.*** *Report confidence intervals or Monte Carlo standard errors more systematically.*

Response. Added. The existing MATLAB paired standard errors are retained for common-random-number comparisons. The independent Python analysis adds 95% bootstrap intervals for CE, VaR_5, and ES_5, plus Wilson intervals for warning probability. S1 and S2 Tables report these intervals, while the methods state the path and bootstrap counts. We now state explicitly that main-text Tables 3–7 are mechanism, frontier, sensitivity, or numerical-diagnostic summaries rather than a complete set of inferential tables. We also clarify that the across-design range in S3 Table is a parameter-sensitivity range, not a population confidence interval.

Location in revised manuscript. Numerical Analysis: implementation/design and independent Python checks; S1–S3 Tables.

Closing

We thank the editor and reviewers again. The comments led us to sharpen the manuscript's terminology and contribution, improve its accessibility, add an independent Python numerical audit, quantify uncertainty, and state the evidential limits more clearly. We hope the revised manuscript now addresses the conceptual, numerical, presentation, and reproducibility concerns in full.

Sincerely,
The Authors
