# Supplementary material for: Robust optimal reinsurance and ESG investment under rating-innovation ambiguity and ESG rating uncertainty
Source: PLoS One. 2026 Nov 2;21(11):e0354616. doi: 10.1371/journal.pone.0354616 (PMC13726995; doi:10.1371/journal.pone.0354616)
Supplement: Supplementary file 3 [file pone.0354616.s002.pdf]

Referee Report: Robust Optimal Reinsurance and ESG Investment under Greenwashing Ambiguity and ESG Rating Uncertainty

Summary

The paper studies a continuous-time reinsurance and investment problem, where an insurer has to choose in a green stock and a brown stock when the true green quality is hidden. The insurer's decision is then affected by the hidden state, which is jointly learned from the returns of the green and orthogonalized brown stock, and an ESG rating signal; in particular, the rating signal is informative but noisy, and it may be also affected by greenwashing ambiguity, that is represented by the worst-case drift distortion of the rating innovation. The analysis enable to separate ordinary ESG rating uncertainty from greenwashing ambiguity, reformulating then a partial-information problem as a separated robust control problem. The authors show that (*i*) stock-return innovations enter both wealth and beliefs, meaning that the separated HJBI equation contains a mixed derivative term; in particular, this implies the optimal stock rule decomposes into myopic and filtering-hedging components. Furthermore, they show that: (*ii*) under CARA utility and a value-dependent ambiguity penalty, the practical reinsurance constraint can be incorporated directly into the main HJBI, and (*iii*) a conditional verification result can be provided under explicit admissibility and integrability conditions, with affine Markov feedbacks as a checkable sufficient subclass. Finally, it is provided and discussed an economic materiality analysis by illustrating interesting numerical simulations of the presented results.

Evaluation

The model presented in the paper is interesting, and the results are sufficiently novel to grant a publication in PLOS One. In general, the paper is well written and mathematically rigorous, as well as the proofs; however, for an audience less familiar with the topic, I suggest the addition of a brief section of preliminary notions. To conclude, I suggest this paper for publication.
