# Supplementary material for: Robust optimal reinsurance and ESG investment under rating-innovation ambiguity and ESG rating uncertainty
Source: PLoS One. 2026 Nov 2;21(11):e0354616. doi: 10.1371/journal.pone.0354616 (PMC13726995; doi:10.1371/journal.pone.0354616)
Supplement: Supplementary file 3 [file pone.0354616.s001.zip › 04_Supporting_Information_Individual_Upload/S1_Code/numerics_matlab/output/figures/fig1_filter_riccati.pdf]

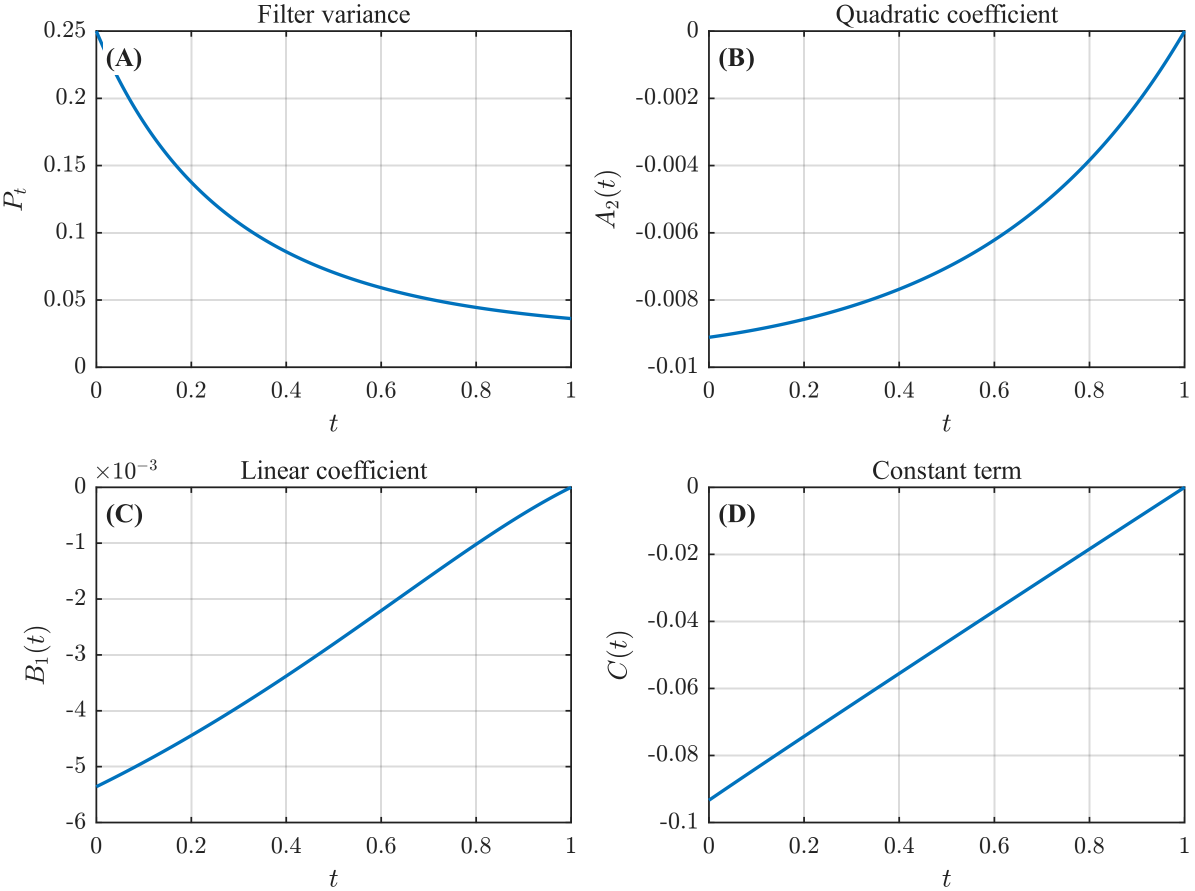
Filter variance
(A)
P_t
t
Quadratic coefficient
(B)
$A_2(t)$
t
Linear coefficient
$\times 10^{-3}$
(C)
$B_1(t)$
t
Constant term
(D)
$C(t)$
t
