# Supplementary material for: Robust optimal reinsurance and ESG investment under rating-innovation ambiguity and ESG rating uncertainty
Source: PLoS One. 2026 Nov 2;21(11):e0354616. doi: 10.1371/journal.pone.0354616 (PMC13726995; doi:10.1371/journal.pone.0354616)
Supplement: Supplementary file 3 [file pone.0354616.s001.zip › 04_Supporting_Information_Individual_Upload/S1_Code/numerics_matlab/output/figures/fig2_hedging_decomposition.pdf]

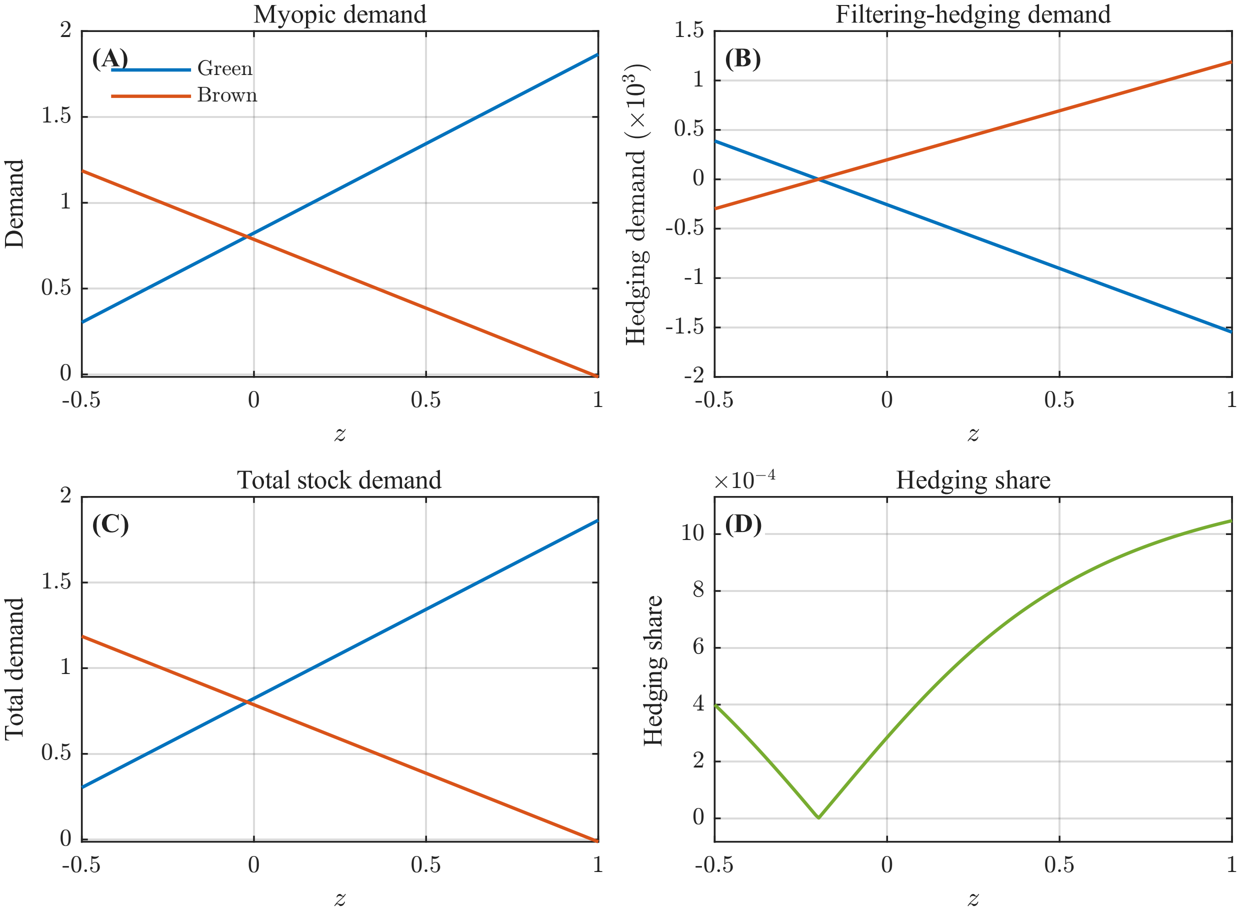

Myopic demand
(A)
Green
Brown
Demand
z
Filtering-hedging demand
(B)
Hedging demand $(\times 10^3)$
z
Total stock demand
(C)
Total demand
z
Hedging share
$\times 10^{-4}$
(D)
Hedging share
z
