# Supplementary material for: Robust optimal reinsurance and ESG investment under rating-innovation ambiguity and ESG rating uncertainty
Source: PLoS One. 2026 Nov 2;21(11):e0354616. doi: 10.1371/journal.pone.0354616 (PMC13726995; doi:10.1371/journal.pone.0354616)
Supplement: Supplementary file 3 [file pone.0354616.s001.zip › 04_Supporting_Information_Individual_Upload/S1_Code/numerics_matlab/output/figures/fig3_learning_comparison.pdf]

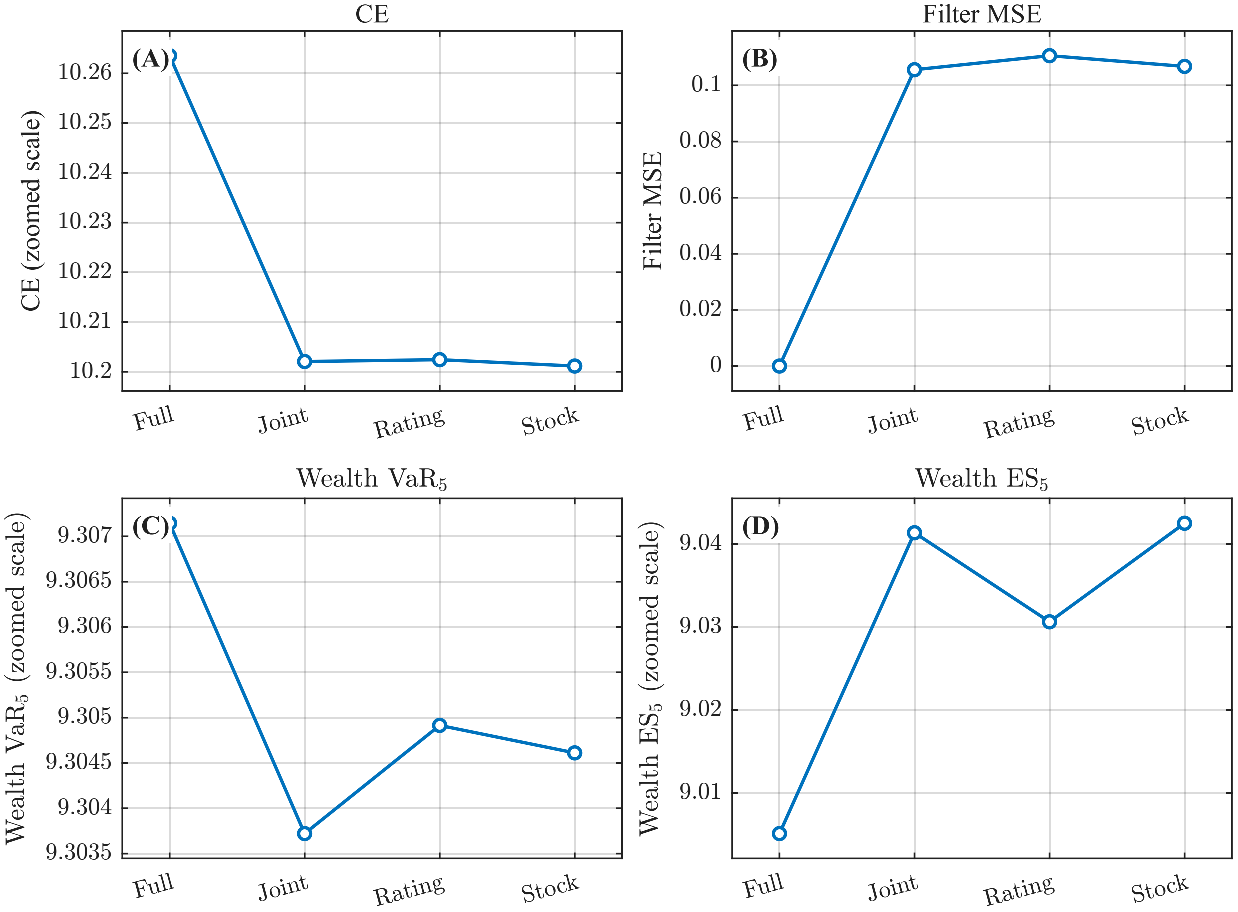
CE
(A)
CE (zoomed scale)
10.26
10.25
10.24
10.23
10.22
10.21
10.2
Full
Joint
Rating
Stock
Filter MSE
(B)
Filter MSE
0.1
0.08
0.06
0.04
0.02
0
Full
Joint
Rating
Stock
Wealth VaR_5
(C)
Wealth VaR_5 (zoomed scale)
9.307
9.3065
9.306
9.3055
9.305
9.3045
9.304
9.3035
Full
Joint
Rating
Stock
Wealth ES_5
(D)
Wealth ES_5 (zoomed scale)
9.04
9.03
9.02
9.01
Full
Joint
Rating
Stock
