# Supplementary material for: Robust optimal reinsurance and ESG investment under rating-innovation ambiguity and ESG rating uncertainty
Source: PLoS One. 2026 Nov 2;21(11):e0354616. doi: 10.1371/journal.pone.0354616 (PMC13726995; doi:10.1371/journal.pone.0354616)
Supplement: Supplementary file 3 [file pone.0354616.s001.zip › 04_Supporting_Information_Individual_Upload/S1_Code/numerics_matlab/output/figures/fig4_channel_decomposition.pdf]

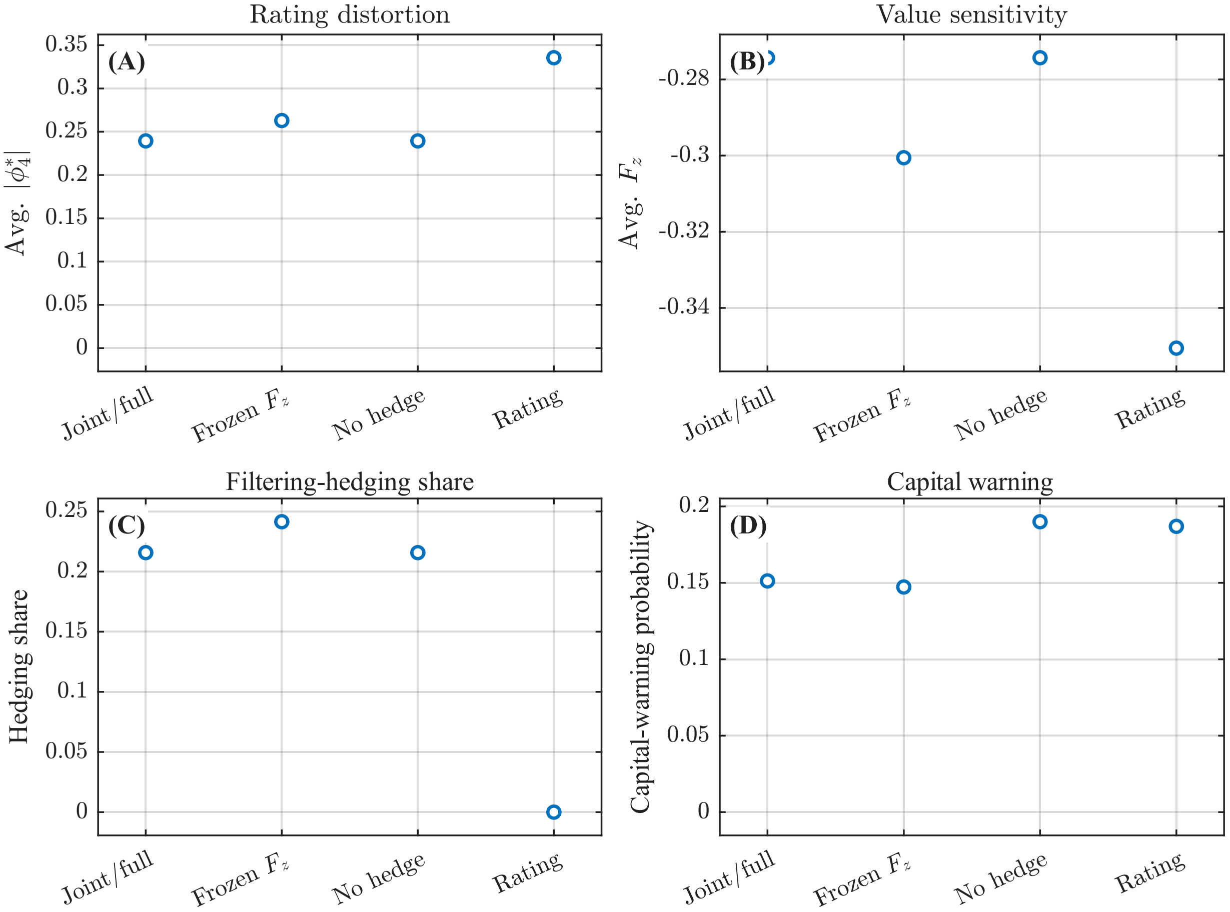

Rating distortion
(A)
Avg. $|\phi_4^*|$
0.35
0.3
0.25
0.2
0.15
0.1
0.05
0
Joint/full
Frozen F_z
No hedge
Rating
Value sensitivity
(B)
Avg. F_z
-0.28
-0.3
-0.32
-0.34
Joint/full
Frozen F_z
No hedge
Rating
Filtering-hedging share
(C)
Hedging share
0.25
0.2
0.15
0.1
0.05
0
Joint/full
Frozen F_z
No hedge
Rating
Capital warning
(D)
Capital-warning probability
0.2
0.15
0.1
0.05
0
Joint/full
Frozen F_z
No hedge
Rating
