# Supplementary material for: Robust optimal reinsurance and ESG investment under rating-innovation ambiguity and ESG rating uncertainty
Source: PLoS One. 2026 Nov 2;21(11):e0354616. doi: 10.1371/journal.pone.0354616 (PMC13726995; doi:10.1371/journal.pone.0354616)
Supplement: Supplementary file 3 [file pone.0354616.s001.zip › 04_Supporting_Information_Individual_Upload/S1_Code/numerics_matlab/output/figures/fig5_materiality_frontier_sigmaY.pdf]

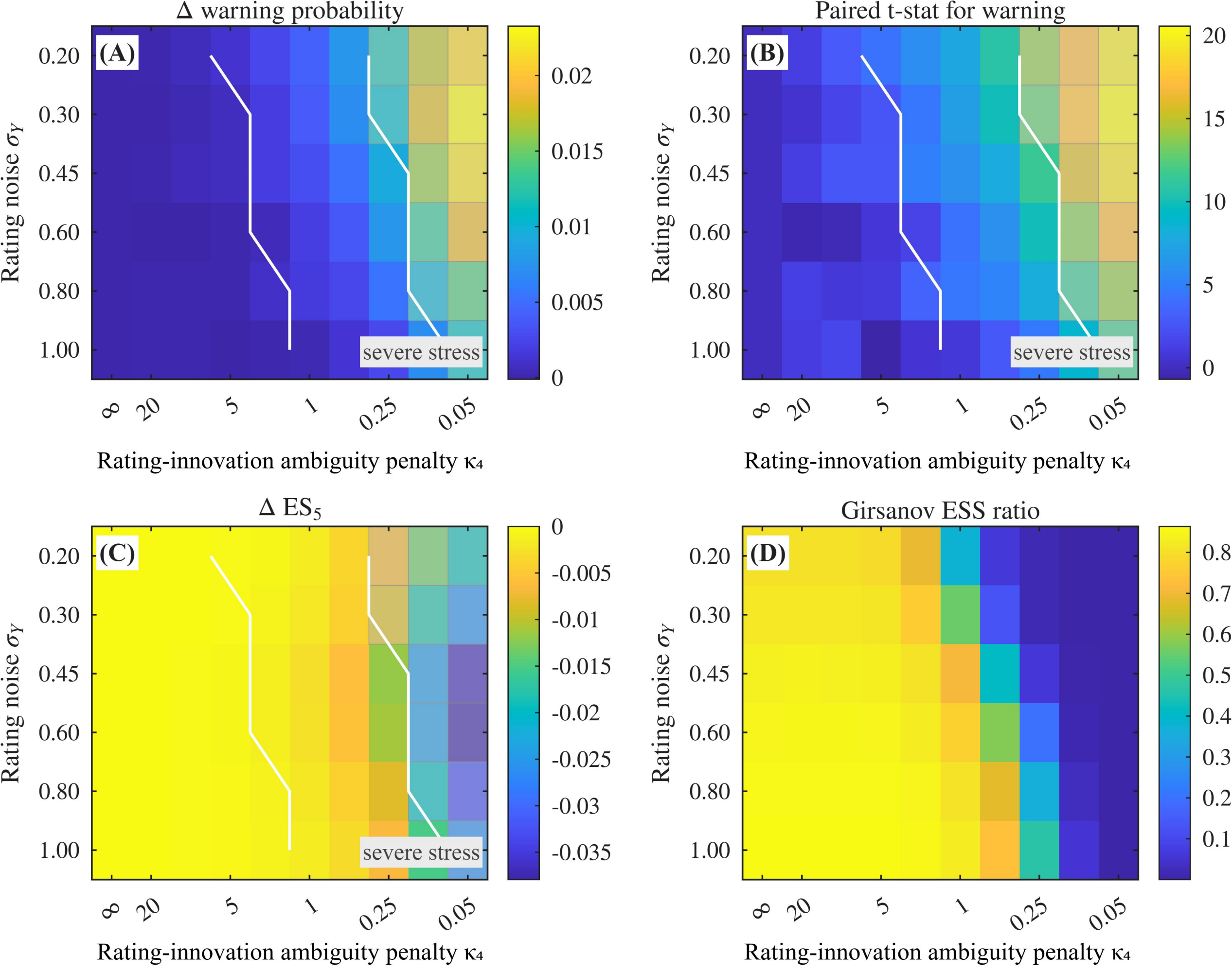

Δ warning probability
Paired t-stat for warning
Δ ES5
Girsanov ESS ratio
(A)
(B)
(C)
(D)
severe stress
Rating noise σY
Rating-innovation ambiguity penalty κ4
0.20
0.30
0.45
0.60
0.80
1.00
∞
20
5
1
0.25
0.05
