# Supplementary material for: Robust optimal reinsurance and ESG investment under rating-innovation ambiguity and ESG rating uncertainty
Source: PLoS One. 2026 Nov 2;21(11):e0354616. doi: 10.1371/journal.pone.0354616 (PMC13726995; doi:10.1371/journal.pone.0354616)
Supplement: Supplementary file 3 [file pone.0354616.s001.zip › 04_Supporting_Information_Individual_Upload/S1_Code/numerics_matlab/output/figures/fig5b_materiality_frontier_alphaZ.pdf]

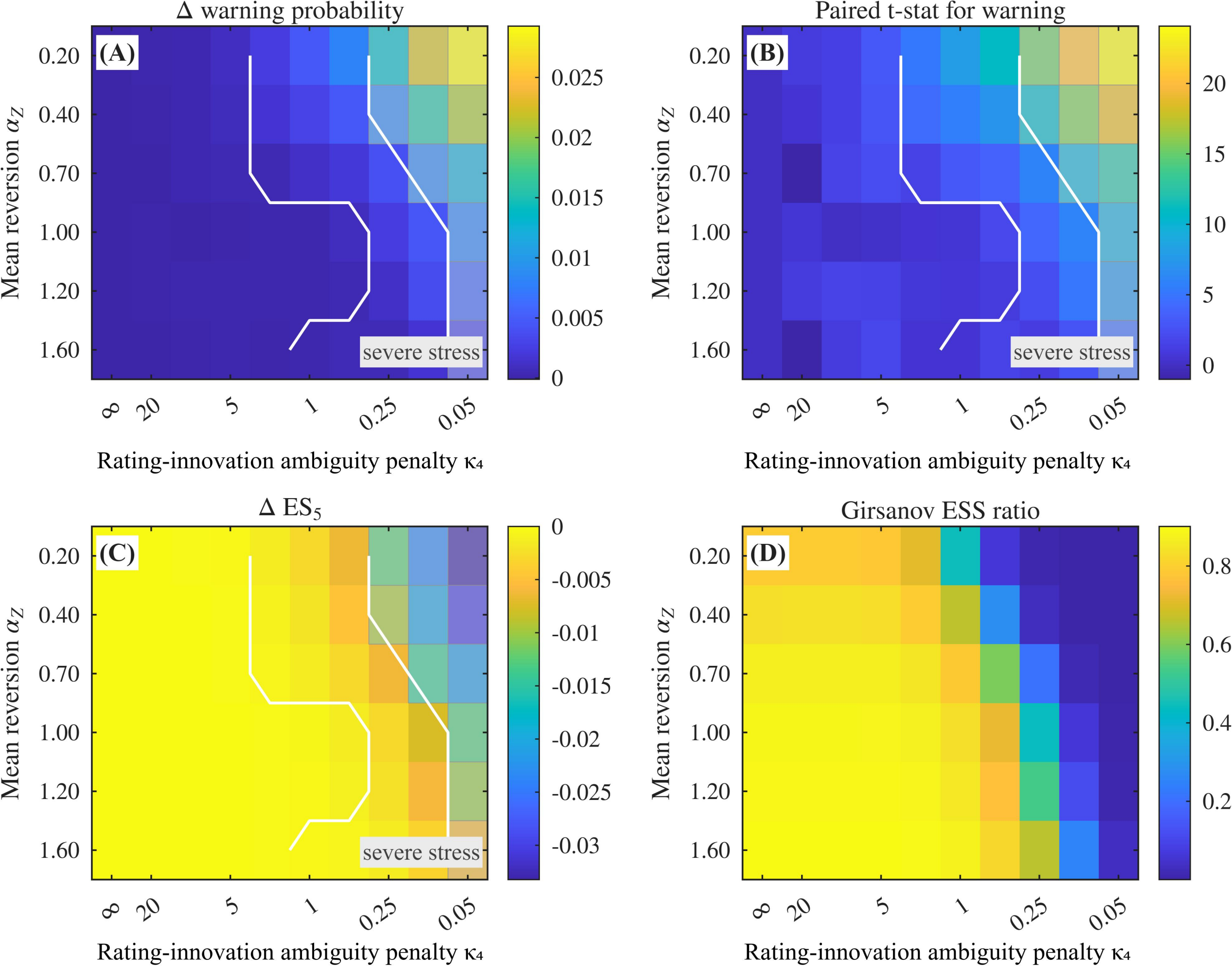

Δ warning probability
(A)
severe stress
Mean reversion α_Z
0.20
0.40
0.70
1.00
1.20
1.60
∞
20
5
1
0.25
0.05
Rating-innovation ambiguity penalty κ4
0.025
0.02
0.015
0.01
0.005
0
Paired t-stat for warning
(B)
20
15
10
5
0
Δ ES_5
(C)
0
-0.005
-0.01
-0.015
-0.02
-0.025
-0.03
Girsanov ESS ratio
(D)
0.8
0.6
0.4
0.2
