# Supplementary material for: Robust optimal reinsurance and ESG investment under rating-innovation ambiguity and ESG rating uncertainty
Source: PLoS One. 2026 Nov 2;21(11):e0354616. doi: 10.1371/journal.pone.0354616 (PMC13726995; doi:10.1371/journal.pone.0354616)
Supplement: Supplementary file 3 [file pone.0354616.s001.zip › 04_Supporting_Information_Individual_Upload/S1_Code/numerics_matlab/output/figures/fig6_interior_reinsurance.pdf]

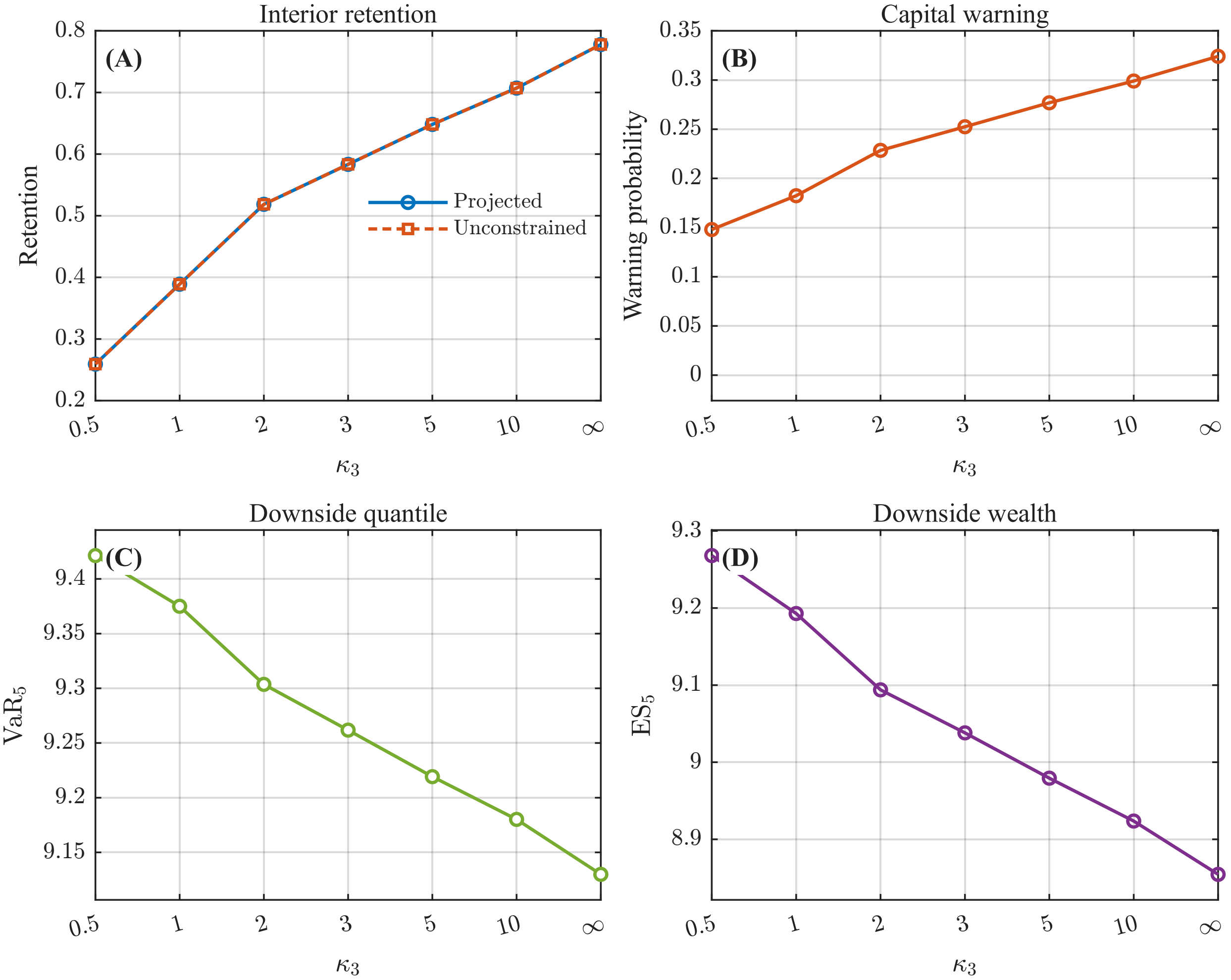

Interior retention
(A)
Retention
Projected
Unconstrained
κ_3
Capital warning
(B)
Warning probability
κ_3
Downside quantile
(C)
VaR_5
κ_3
Downside wealth
(D)
ES_5
κ_3
