# Supplementary material for: Robust optimal reinsurance and ESG investment under rating-innovation ambiguity and ESG rating uncertainty
Source: PLoS One. 2026 Nov 2;21(11):e0354616. doi: 10.1371/journal.pone.0354616 (PMC13726995; doi:10.1371/journal.pone.0354616)
Supplement: Supplementary file 3 [file pone.0354616.s001.zip › 04_Supporting_Information_Individual_Upload/S1_Code/numerics_matlab/output/figures/fig7_misspecification.pdf]

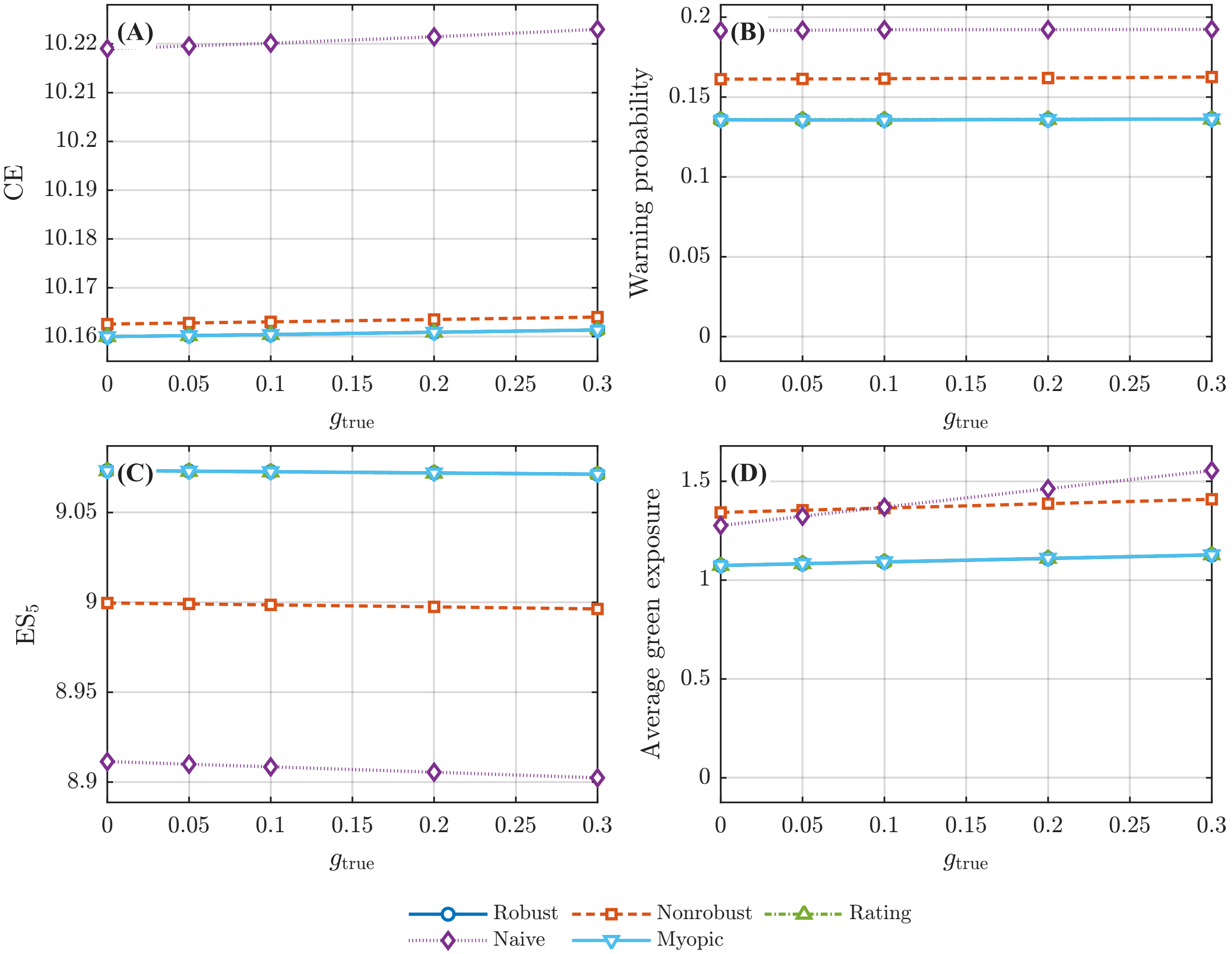

(A)
CE
10.22
10.21
10.2
10.19
10.18
10.17
10.16
0
0.05
0.1
0.15
0.2
0.25
0.3
$g_{\text{true}}$
(B)
Warning probability
0.2
0.15
0.1
0.05
0
(C)
$ES_5$
9.05
9
8.95
8.9
(D)
Average green exposure
1.5
1
0.5
0
Robust
Nonrobust
Rating
Naive
Myopic
