# Supplementary material for: Robust optimal reinsurance and ESG investment under rating-innovation ambiguity and ESG rating uncertainty
Source: PLoS One. 2026 Nov 2;21(11):e0354616. doi: 10.1371/journal.pone.0354616 (PMC13726995; doi:10.1371/journal.pone.0354616)
Supplement: Supplementary file 3 [file pone.0354616.s001.zip › 04_Supporting_Information_Individual_Upload/S1_Code/numerics_python/output/figures/figS2_learning_original_scale.pdf]

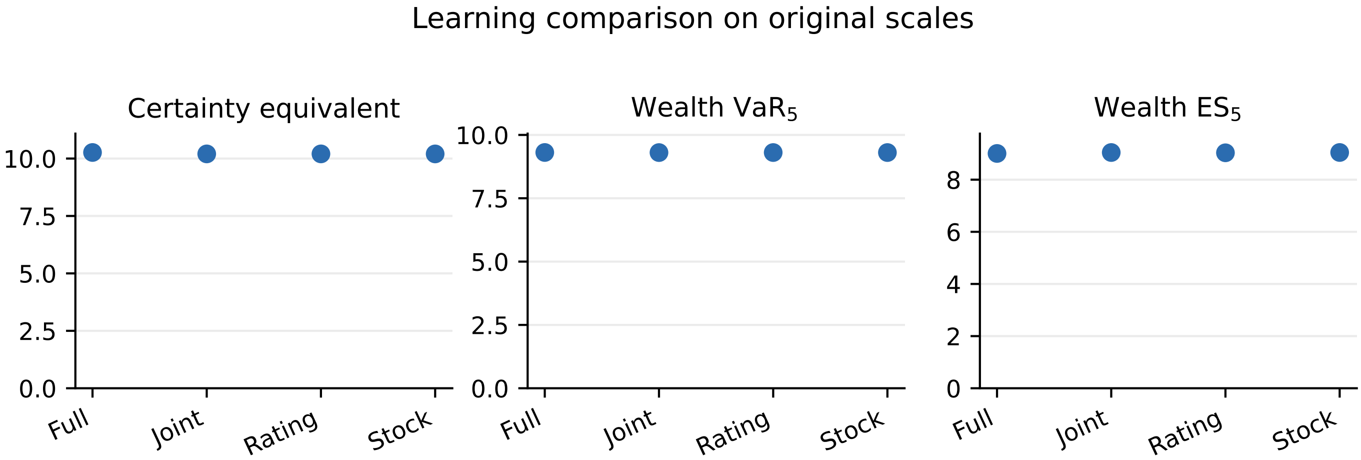

Learning comparison on original scales
Certainty equivalent
Wealth VaR5
Wealth ES5
0.0
2.5
5.0
7.5
10.0
0
2
4
6
8
Full
Joint
Rating
Stock
