# Supplementary material for: Robust optimal reinsurance and ESG investment under rating-innovation ambiguity and ESG rating uncertainty
Source: PLoS One. 2026 Nov 2;21(11):e0354616. doi: 10.1371/journal.pone.0354616 (PMC13726995; doi:10.1371/journal.pone.0354616)
Supplement: Supplementary file 3 [file pone.0354616.s001.zip › 04_Supporting_Information_Individual_Upload/S1_Code/numerics_python/output/figures/figS3_misspecification_original_scale.pdf]

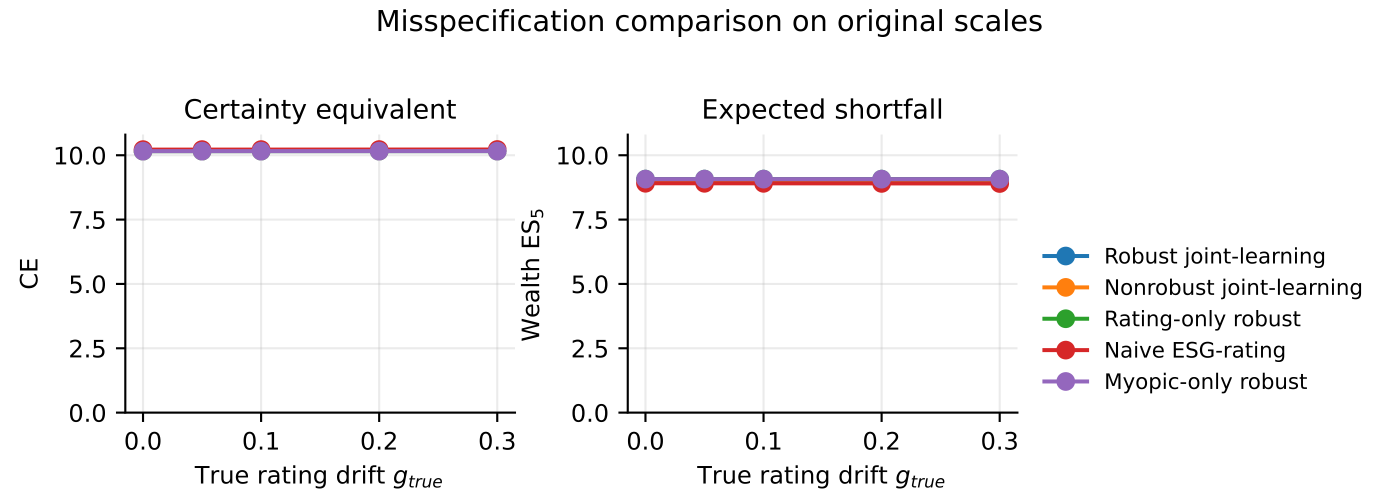
Misspecification comparison on original scales
Certainty equivalent
CE
10.0
7.5
5.0
2.5
0.0
0.0
0.1
0.2
0.3
True rating drift g_{true}
Expected shortfall
Wealth ES_5
10.0
7.5
5.0
2.5
0.0
0.0
0.1
0.2
0.3
True rating drift g_{true}
Robust joint-learning
Nonrobust joint-learning
Rating-only robust
Naive ESG-rating
Myopic-only robust
