# Supplementary material for: Robust optimal reinsurance and ESG investment under rating-innovation ambiguity and ESG rating uncertainty
Source: PLoS One. 2026 Nov 2;21(11):e0354616. doi: 10.1371/journal.pone.0354616 (PMC13726995; doi:10.1371/journal.pone.0354616)
Supplement: Supplementary file 3 [file pone.0354616.s001.zip › 04_Supporting_Information_Individual_Upload/S1_Code/numerics_python/output/figures/figS4_python_parameter_uncertainty.pdf]

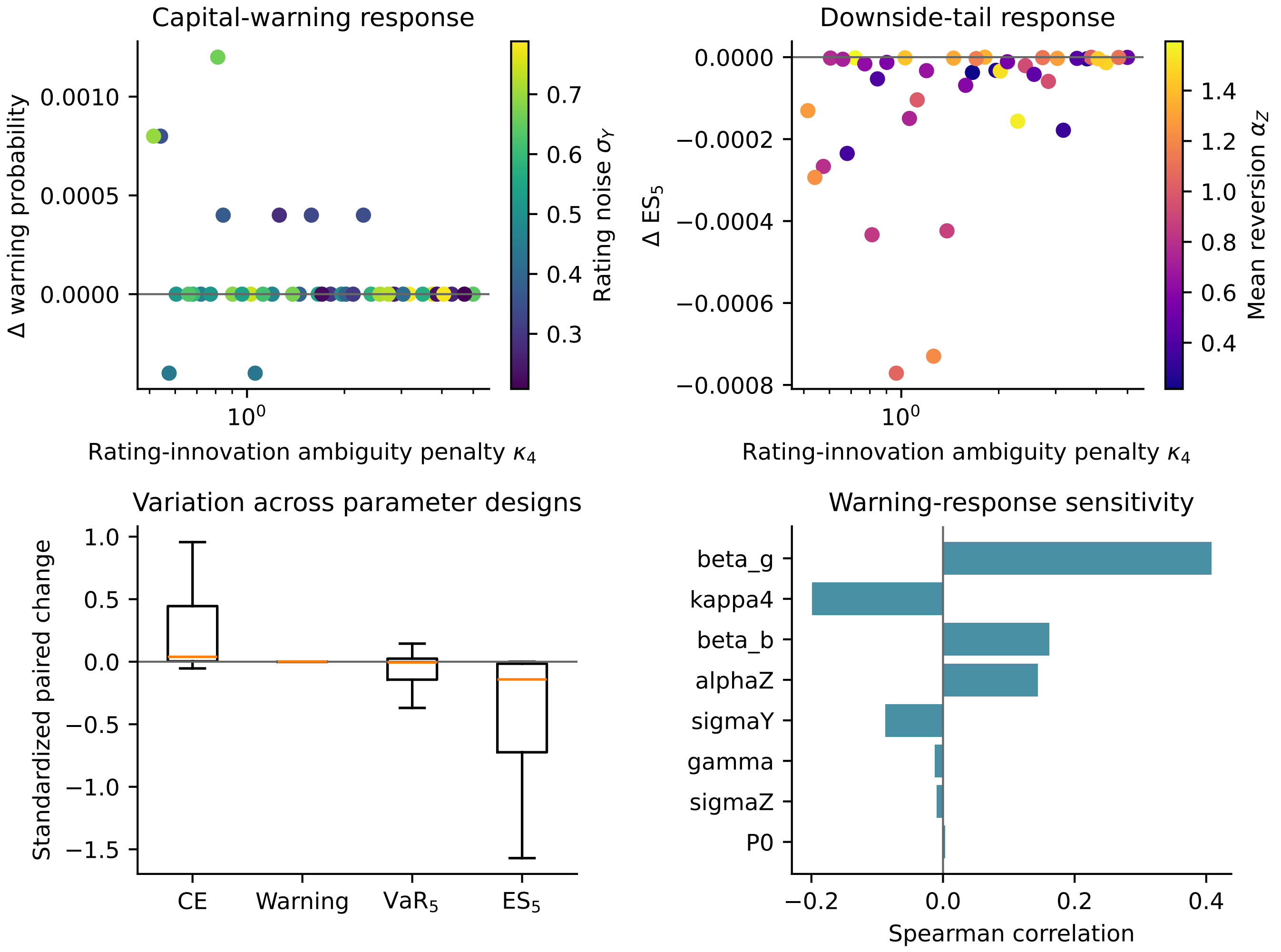

Capital-warning response
Δ warning probability
0.0010
0.0005
0.0000
10^0
Rating-innovation ambiguity penalty κ_4
Rating noise σ_Y
0.7
0.6
0.5
0.4
0.3
Downside-tail response
Δ ES_5
0.0000
−0.0002
−0.0004
−0.0006
−0.0008
10^0
Rating-innovation ambiguity penalty κ_4
Mean reversion α_Z
1.4
1.2
1.0
0.8
0.6
0.4
Variation across parameter designs
Standardized paired change
1.0
0.5
0.0
−0.5
−1.0
−1.5
CE
Warning
VaR_5
ES_5
Warning-response sensitivity
beta_g
kappa4
beta_b
alphaZ
sigmaY
gamma
sigmaZ
P0
−0.2
0.0
0.2
0.4
Spearman correlation
