# Supplementary material for: Robust optimal reinsurance and ESG investment under rating-innovation ambiguity and ESG rating uncertainty
Source: PLoS One. 2026 Nov 2;21(11):e0354616. doi: 10.1371/journal.pone.0354616 (PMC13726995; doi:10.1371/journal.pone.0354616)
Supplement: Supplementary file 3 [file pone.0354616.s001.zip › 04_Supporting_Information_Individual_Upload/S1_Fig.pdf]

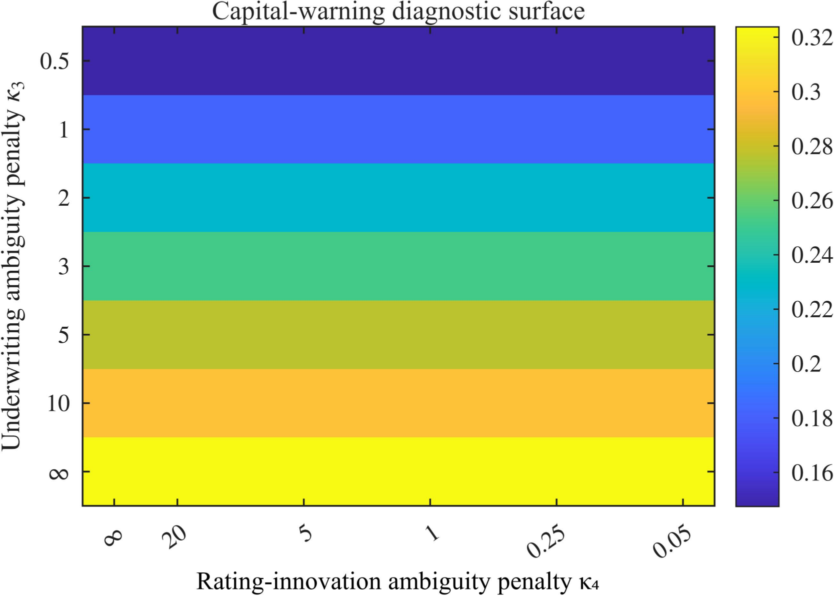
Capital-warning diagnostic surface
Underwriting ambiguity penalty κ3
0.5
1
2
3
5
10
∞
Rating-innovation ambiguity penalty κ4
∞
20
5
1
0.25
0.05
0.32
0.3
0.28
0.26
0.24
0.22
0.2
0.18
0.16
