# Supplementary material for: Robust optimal reinsurance and ESG investment under rating-innovation ambiguity and ESG rating uncertainty
Source: PLoS One. 2026 Nov 2;21(11):e0354616. doi: 10.1371/journal.pone.0354616 (PMC13726995; doi:10.1371/journal.pone.0354616)
Supplement: Peer review history

# Peer review: Decision Letter 0

**DOI:** 10.1371/journal.pone.0354616.r001

**Authors:** Alessandro Mazzoccoli

29 Jul 2026

Dear Dr. Zhou,

Thank you for submitting your manuscript to PLOS ONE. After careful consideration, we feel that it has merit but does not fully meet PLOS ONE’s publication criteria as it currently stands. Therefore, we invite you to submit a revised version of the manuscript that addresses the points raised during the review process.

After carefully considering the reviewers’ reports, I have decided to recommend a major revision of the manuscript. I strongly encourage the authors to address all the comments and suggestions raised by the reviewers with due attention, as these revisions may substantially improve the quality, clarity, and overall contribution of the article.

Please submit your revised manuscript by Sep 27 2026 11:59PM. If you will need more time than this to complete your revisions, please reply to this message or contact the journal office at plosone@plos.org. When you're ready to submit your revision, log on to https://www.editorialmanager.com/pone/ and select the 'Submissions Needing Revision' folder to locate your manuscript file.

- A letter that responds to each point raised by the academic editor and reviewer(s). You should upload this letter as a separate file labeled 'Response to Reviewers'.
- A marked-up copy of your manuscript that highlights changes made to the original version. You should upload this as a separate file labeled 'Revised Manuscript with Track Changes'.
- An unmarked version of your revised paper without tracked changes. You should upload this as a separate file labeled 'Manuscript'.

If you would like to make changes to your financial disclosure, please include your updated statement in your cover letter. Guidelines for resubmitting your figure files are available below the reviewer comments at the end of this letter.

If applicable, we recommend that you deposit your laboratory protocols in protocols.io to enhance the reproducibility of your results. Protocols.io assigns your protocol its own identifier (DOI) so that it can be cited independently in the future. For instructions see: https://journals.plos.org/plosone/s/submission-guidelines#loc-laboratory-protocols. Additionally, PLOS ONE offers an option for publishing peer-reviewed Lab Protocol articles, which describe protocols hosted on protocols.io. Read more information on sharing protocols at https://plos.org/protocols?utm_medium=editorial-email&utm_source=authorletters&utm_campaign=protocols.

As the corresponding author, your ORCID iD is verified in the submission system and will appear in the published article. PLOS supports the use of ORCID, and we encourage all coauthors to register for an ORCID iD and use it as well. Please encourage your coauthors to verify their ORCID iD within the submission system before final acceptance, as unverified ORCID iDs will not appear in the published article. *Only*  the individual author can complete the verification step; PLOS staff *cannot*  verify ORCID iDs on behalf of authors.

We look forward to receiving your revised manuscript.

Kind regards,

Alessandro Mazzoccoli, Ph.D.

Academic Editor

PLOS One

Journal Requirements:

When submitting your revision, we need you to address these additional requirements.

1. Please ensure that your manuscript meets PLOS ONE's style requirements, including those for file naming. The PLOS ONE style templates can be found at

https://journals.plos.org/plosone/s/file?id=wjVg/PLOSOne_formatting_sample_main_body.pdf and

https://journals.plos.org/plosone/s/file?id=ba62/PLOSOne_formatting_sample_title_authors_affiliations.pdf

2. Please note that PLOS One has specific guidelines on code sharing for submissions in which author-generated code underpins the findings in the manuscript. In these cases, we expect all author-generated code to be made available without restrictions upon publication of the work. Please review our guidelines at https://journals.plos.org/plosone/s/materials-and-software-sharing#loc-sharing-code and ensure that your code is shared in a way that follows best practice and facilitates reproducibility and reuse.

3. Please update your submission to use the PLOS LaTeX template. The template and more information on our requirements for LaTeX submissions can be found at http://journals.plos.org/plosone/s/latex.

4. Thank you for stating the following in the Acknowledgments Section of your manuscript:

“This work has received financial support by Yunnan Provincial Department of Education Scientific Research Fund Project (Grant No.: 2026J1134), Leshan Philosophy and Social Science Planning Project (Grant No. SKL2025D31), Leshan Normal University Scientific Star-up Project for Introducing High-level Talents (Grants No. RC2025012).“

We note that you have provided funding information that is not currently declared in your Funding Statement. However, funding information should not appear in the Acknowledgments section or other areas of your manuscript. We will only publish funding information present in the Funding Statement section of the online submission form.

Please remove any funding-related text from the manuscript and let us know how you would like to update your Funding Statement. Currently, your Funding Statement reads as follows:

“Jing Fu gratefully acknowledges the support from Yunnan Provincial Department of Education Scientific Research Fund Project (Grant No.: 2026J1134), Guoyong Zhou gratefully acknowledges the support from Leshan Philosophy and Social Science Planning Project (Grant No. SKL2025D31), and Leshan Normal University Scientific Star-up Project for Introducing High-level Talents (Grants No. RC2025012).”

Please include your amended statements within your cover letter; we will change the online submission form on your behalf.

5. Thank you for stating the following financial disclosure:

“Jing Fu gratefully acknowledges the support from Yunnan Provincial Department of Education Scientific Research Fund Project (Grant No.: 2026J1134), Guoyong Zhou gratefully acknowledges the support from Leshan Philosophy and Social Science Planning Project (Grant No. SKL2025D31), and Leshan Normal University Scientific Star-up Project for Introducing High-level Talents (Grants No. RC2025012).”

Please state what role the funders took in the study.  If the funders had no role, please state: "The funders had no role in study design, data collection and analysis, decision to publish, or preparation of the manuscript."

If this statement is not correct you must amend it as needed.

Please include this amended Role of Funder statement in your cover letter; we will change the online submission form on your behalf.

6. Thank you for stating the following financial disclosure:

“Jing Fu gratefully acknowledges the support from Yunnan Provincial Department of Education Scientific Research Fund Project (Grant No.: 2026J1134), Guoyong Zhou gratefully acknowledges the support from Leshan Philosophy and Social Science Planning Project (Grant No. SKL2025D31), and Leshan Normal University Scientific Star-up Project for Introducing High-level Talents (Grants No. RC2025012).”

We note that one or more of the authors is affiliated with the funding organization, indicating the funder may have had some role in the design, data collection, analysis or preparation of your manuscript for publication; in other words, the funder played an indirect role through the participation of the co-authors. If the funding organization did not play a role in the study design, data collection and analysis, decision to publish, or preparation of the manuscript and only provided financial support in the form of authors' salaries and/or research materials, please do the following:

1. Review your statements relating to the author contributions, and ensure you have specifically and accurately indicated the role(s) that these authors had in your study. These amendments should be made in the online form.

2. Confirm in your cover letter that you agree with the following statement, and we will change the online submission form on your behalf:

“The funder provided support in the form of salaries for authors [insert relevant initials], but did not have any additional role in the study design, data collection and analysis, decision to publish, or preparation of the manuscript. The specific roles of these authors are articulated in the ‘author contributions’ section.

7. Please provide a complete Data Availability Statement in the submission form, ensuring you include all necessary access information or a reason for why you are unable to make your data freely accessible. If your research concerns only data provided within your submission, please write "All data are in the manuscript and/or supporting information files" as your Data Availability Statement.

8. We note you have included a table to which you do not refer in the text of your manuscript. Please ensure that you refer to Table 4 in your text; if accepted, production will need this reference to link the reader to the Table.

9. If the reviewer comments include a recommendation to cite specific previously published works, please review and evaluate these publications to determine whether they are relevant and should be cited. There is no requirement to cite these works unless the editor has indicated otherwise.

[Note: HTML markup is below. Please do not edit.]

Reviewers' comments:

Reviewer's Responses to Questions

**Comments to the Author**

1. Is the manuscript technically sound, and do the data support the conclusions?

Reviewer #1: Yes

Reviewer #2: Yes

Reviewer #3: Partly

**********

2. Has the statistical analysis been performed appropriately and rigorously?

Reviewer #1: Yes

Reviewer #2: I Don't Know

Reviewer #3: Yes

**********

3. Have the authors made all data underlying the findings in their manuscript fully available?

The PLOS Data policy

Reviewer #1: Yes

Reviewer #2: Yes

Reviewer #3: Yes

**********

4. Is the manuscript presented in an intelligible fashion and written in standard English?

Reviewer #1: Yes

Reviewer #2: Yes

Reviewer #3: Yes

**********

Reviewer #1: In the numerical experiment (section 5) the authors use some parameters, but I didn't understand from where those values came from, so I would suggest some comments on them just to help the reader. If some real data where used I suggest to add a section where the data are described.

Reviewer #2: The paper studies a continuous-time reinsurance and investment problem, where an in

surer has to choose in a green stock and a brown stock when the true green quality

is hidden. The insurer’s decision is then affected by the hidden state, which is jointly

learned from the returns of the green and orthogonalized brown stock, and an ESG rating

signal; in particular, the rating signal is informative but noisy, and it may be also af

fected by greenwashing ambiguity, that is represented by the worst-case drift distortion of

the rating innovation. The analysis enable to separate ordinary ESG rating uncertainty

from greenwashing ambiguity, reformulating then a partial-information problem as a sep

arated robust control problem. The authors show that (i) stock-return innovations enter

both wealth and beliefs, meaning that the separated HJBI equation contains a mixed

derivative term; in particular, this implies the optimal stock rule decomposes into my

opic and filtering-hedging components. Furthermore, they show that: (ii) under CARA

utility and a value-dependent ambiguity penalty, the practical reinsurance constraint

can be incorporated directly into the main HJBI, and (iii) a conditional verification re

sult can be provided under explicit admissibility and integrability conditions, with affine

Markov feedbacks as a checkable sufficient subclass. Finally, it is provided and discussed

an economic materiality analysis by illustrating interesting numerical simulations of the

presented results. I reccommend this paper for publication, I just suggest the addition of

a preliminaries section for readers not familiar with the topics

Reviewer #3: The author(s) propose a continuous-time robust portfolio and reinsurance optimisation model under partial information, where the insurer learns a latent green-quality factor through ESG ratings and stock returns. They propose a Kalman–Bucy filtering framework and solved using a robust HJBI approach with exponential utility. The paper combines filtering, robust control and insurance decision-making in a unified framework and is mathematically rigorous and well developed.

I believe that several conceptual and presentation issues should be addressed before the paper can be considered for publication. In particular:

1. Distinction between greenwashing and model ambiguity. The most important issue concerns the interpretation of the fourth ambiguity component. Throughout the paper,the ESG-rating innovation bias is interpreted as greenwashing ambiguity. However, mathematically, the process (\phi_4) is an adversarial drift distortion introduced through robust control, whereas in the numerical section the authors also introduce a genuine misspecification of the ESG-rating process through an additional drift term (g^{\mathrm{true}}). These represent two conceptually different mechanisms: (i) model ambiguity regarding the observation process; (ii) actual misspecification, i.e. systematic bias, in ESG ratings. The author(s) often refer to both mechanisms simply as ``greenwashing'', making the economic interpretation difficult to read. It would be necessary to distinguish the two elements more clearly, with a precise explanation of the role played by each mechanism.

2. Separation between financial risk and informational ambiguity: the method introduces four ambiguity components corresponding to financial returns, underwriting risk and ESG information. However, the interpretation of these components is not entirely transparent. In particular, the first two distortions simultaneously affect both investment returns and belief updating, whereas the fourth component is interpreted as ESG-rating ambiguity. Consequently, the proposed framework does not completely disentangle market uncertainty from information-quality uncertainty. A more explicit discussion of the economic interpretation of each ambiguity component would considerably improve the paper.

3. Numerical evidence mainly validates the mechanism rather than its economic relevance: the numerical analysis is entirely based on illustrative parameters. While this is acceptable for validating the mathematical mechanism, the economic conclusions should be presented more cautiously, as they cannot be interpreted quantitatively without calibration or empirical motivation. Moreover, the reported differences in CE, VaR, and ES are often quantitatively small, and the manuscript does not discuss their economic significance or provide confidence intervals for the main estimates. The sensitivity analysis is mainly local, while a broader assessment of parameter uncertainty would strengthen the robustness of the findings. In addition, the numerical comparisons rely mostly on internal model variants, without external benchmarks. Finally, the discussion is often descriptive rather than explanatory, and the manuscript should better distinguish between demonstrating the existence of the proposed mechanisms and establishing their quantitative economic relevance.

4. Materiality frontier: the concept of ``materiality frontier'' is one of the main numerical contributions but is not formally defined. The paper would benefit from a clearer explanation of: what constitutes materiality; why the proposed criterion is appropriate; and whether alternative definitions could be considered.

5. Standard theoretical results versus original contributions: A substantial portion of the manuscript is devoted to proving results that are largely standard applications of existing theory, including: the Kalman–Bucy filter; the separation principle; the Girsanov transformation; the pointwise optimization of the Hamiltonian. While these results are correct and necessary, much of the exposition reproduces well-established arguments. I recommend moving the proofs of standard results to an Appendix and retaining in the main text only those derivations that are specific to the proposed model. This would considerably improve readability and help emphasise the genuinely original contributions. Furthermore, the manuscript would benefit from a clearer discussion distinguishing standard methodology from methodological novelty.

Minor comments:

The notation could be made more uniform throughout the manuscript. In particular, the probability measure P is introduced as the reference measure, but readers from mathematical finance may initially interpret it as a risk-neutral measure. Explicitly stating that P denotes the physical/reference probability measure would improve clarity.

The Brownian motions W1, W2, W3, and W4 could be introduced more explicitly, with a brief description of the role of each process. In particular, W_3 first appears only in the underwriting equation, making the model harder to follow during the initial reading.

The manuscript simultaneously uses B^Z and W_i to denote Brownian motions. Adopting a single notation throughout would improve consistency.

Some explanations regarding the reference filter and the fact that filtering is not recomputed under the distorted measure are repeated several times across Sections 2 and 3. These discussions could be consolidated to reduce redundancy.

Several figures rely on highly zoomed axes to reveal relatively small differences. Although this is acknowledged by the authors, complementary plots on the original scale could provide a more balanced visual interpretation.

Given the extensive Monte Carlo simulations, reporting confidence intervals or Monte Carlo standard errors more systematically would strengthen the numerical analysis.

**********

what does this mean?). If published, this will include your full peer review and any attached files.

If you choose “no”, your identity will remain anonymous but your review may still be made public.

**Do you want your identity to be public for this peer review?**  For information about this choice, including consent withdrawal, please see our Privacy Policy

Reviewer #1: No

Reviewer #2: No

Reviewer #3: No

**********

[NOTE: If reviewer comments were submitted as an attachment file, they will be attached to this email and accessible via the submission site. Please log into your account, locate the manuscript record, and check for the action link "View Attachments". If this link does not appear, there are no attachment files.]

To ensure your figures meet our technical requirements, please review our figure guidelines: https://journals.plos.org/plosone/s/figures

You may also use PLOS’s free figure tool, NAAS, to help you prepare publication quality figures: https://journals.plos.org/plosone/s/figures#loc-tools-for-figure-preparation.

NAAS will assess whether your figures meet our technical requirements by comparing each figure against our figure specifications.

Attachment Submitted filename: PLOSOne_report.pdf

# Author comment: Author response to Decision Letter 1

**DOI:** 10.1371/journal.pone.0354616.r002

15 Sep 2026

All comments raised by the reviewers and the editor have been carefully addressed, and corresponding revisions have been made to the manuscript. Please see the "Response to Reviewers" document for a point-by-point response to each comment.

Attachment Submitted filename: response_to_reviewers.pdf

# Peer review: Decision Letter 1

**DOI:** 10.1371/journal.pone.0354616.r003

**Authors:** Alessandro Mazzoccoli

4 Oct 2026

Robust optimal reinsurance and ESG investment under rating-innovation ambiguity and ESG rating uncertainty

PONE-D-26-34087R1

Dear Dr. Zhou,

We’re pleased to inform you that your manuscript has been judged scientifically suitable for publication and will be formally accepted for publication once it meets all outstanding technical requirements.

Within one week, you’ll receive an e-mail detailing the required amendments. When these have been addressed, you’ll receive a formal acceptance letter and your manuscript will be scheduled for publication.

An invoice will be generated when your article is formally accepted. Please note, if your institution has a publishing partnership with PLOS and your article meets the relevant criteria, all or part of your publication costs will be covered. Please make sure your user information is up-to-date by logging into Editorial Manager at Editorial Manager® and clicking the ‘Update My Information' link at the top of the page. For questions related to billing, please contact billing support.

If your institution or institutions have a press office, please notify them about your upcoming paper to help maximize its impact. If they’ll be preparing press materials, please inform our press team as soon as possible -- no later than 48 hours after receiving the formal acceptance. Your manuscript will remain under strict press embargo until 2 pm Eastern Time on the date of publication. For more information, please contact onepress@plos.org.

Kind regards,

Alessandro Mazzoccoli, Ph.D.

Academic Editor

PLOS One

Additional Editor Comments (optional):

Reviewers' comments:

Reviewer's Responses to Questions

**Comments to the Author**

Reviewer #1: All comments have been addressed

Reviewer #2: All comments have been addressed

Reviewer #3: All comments have been addressed

**********

2. Is the manuscript technically sound, and do the data support the conclusions?

Reviewer #1: Yes

Reviewer #2: Yes

Reviewer #3: Yes

**********

3. Has the statistical analysis been performed appropriately and rigorously?

Reviewer #1: Yes

Reviewer #2: N/A

Reviewer #3: Yes

**********

4. Have the authors made all data underlying the findings in their manuscript fully available?

The PLOS Data policy

Reviewer #1: Yes

Reviewer #2: Yes

Reviewer #3: Yes

**********

5. Is the manuscript presented in an intelligible fashion and written in standard English?

Reviewer #1: Yes

Reviewer #2: Yes

Reviewer #3: Yes

**********

Reviewer #1: Nothing to report. As for me the paper can be publishd. I think the authors have met al the recommendations and the paper could be published.

Reviewer #2: (No Response)

Reviewer #3: I thank the authors for improving the manuscript based on my suggestions. I consider the paper suitable for publication in its current form.

**********

what does this mean?). If published, this will include your full peer review and any attached files.

If you choose “no”, your identity will remain anonymous but your review may still be made public.

**Do you want your identity to be public for this peer review?**  For information about this choice, including consent withdrawal, please see our Privacy Policy

Reviewer #1: No

Reviewer #2: No

Reviewer #3: No

**********

# Editor report: Acceptance letter

**DOI:** 10.1371/journal.pone.0354616.r004

**Authors:** Alessandro Mazzoccoli

PONE-D-26-34087R1

PLOS One

Dear Dr. Zhou,

I'm pleased to inform you that your manuscript has been deemed suitable for publication in PLOS One. Congratulations! Your manuscript is now being handed over to our production team.

At this stage, our production department will prepare your paper for publication. This includes ensuring the following:

* All references, tables, and figures are properly cited

* All relevant supporting information is included in the manuscript submission,

* There are no issues that prevent the paper from being properly typeset

You will receive further instructions from the production team, including instructions on how to review your proof when it is ready. Please keep in mind that we are working through a large volume of accepted articles, so please give us a few days to review your paper and let you know the next and final steps.

Lastly, if your institution or institutions have a press office, please let them know about your upcoming paper now to help maximize its impact. If they'll be preparing press materials, please inform our press team within the next 48 hours. Your manuscript will remain under strict press embargo until 2 pm Eastern Time on the date of publication. For more information, please contact onepress@plos.org.

You will receive an invoice from PLOS for your publication fee after your manuscript has reached the completed accept phase. If you receive an email requesting payment before acceptance or for any other service, this may be a phishing scheme. Learn how to identify phishing emails and protect your accounts at https://explore.plos.org/phishing.

If we can help with anything else, please email us at customercare@plos.org.

Thank you for submitting your work to PLOS One and supporting open access.

Kind regards,

PLOS One Editorial Office Staff

on behalf of

Dr. Alessandro Mazzoccoli

Academic Editor

PLOS One
